# Longitudinal trajectories of cortical development in 22q11.2 copy number variants and typically developing controls

**DOI:** 10.1038/s41380-022-01681-w

**Published:** 2022-07-27

**Authors:** Maria Jalbrzikowski, Amy Lin, Ariana Vajdi, Vardui Grigoryan, Leila Kushan, Christopher R. K. Ching, Charles Schleifer, Rebecca A. Hayes, Stephanie A. Chu, Catherine A. Sugar, Jennifer K. Forsyth, Carrie E. Bearden

**Affiliations:** 1 Department of Psychiatry and Behavioral Sciences, Boston Children’s Hospital, Boston, MA USA; 2 Department of Psychiatry, Harvard Medical School, Boston, MA USA; 3 Department of Psychiatry and Biobehavioral Sciences, Semel Institute for Neuroscience and Human Behavior, University of California, Los Angeles, CA USA; 4 Neuroscience Interdepartmental Program, University of California, Los Angeles, CA USA; 5 Imaging Genetics Center, Mark and Mary Stevens Neuroimaging and Informatics Institute, Keck School of Medicine, University of Southern California, Marina del Rey, CA USA; 6 Department of Psychiatry, University of Pittsburgh School of Medicine, Pittsburgh, PA USA; 7 Department of Biostatistics, University of California, Los Angeles, CA, USA; 8 Department of Psychology, University of Washington, Seattle, WA, USA; 9 Department of Psychology, University of California, Los Angeles, CA USA

**Keywords:** Genetics, Schizophrenia

## Abstract

Probing naturally-occurring, reciprocal genomic copy number variations (CNVs) may help us understand mechanisms that underlie deviations from typical brain development. Cross-sectional studies have identified prominent reductions in cortical surface area (SA) and increased cortical thickness (CT) in 22q11.2 deletion carriers (22qDel), with the opposite pattern in duplication carriers (22qDup), but the longitudinal trajectories of these anomalies—and their relationship to clinical symptomatology—are unknown. Here, we examined neuroanatomic changes within a longitudinal cohort of 261 22q11.2 CNV carriers and demographically-matched typically developing (TD) controls (84 22qDel, 34 22qDup, and 143 TD; mean age 18.35, ±10.67 years; 50.47% female). A total of 431 magnetic resonance imaging scans (164 22qDel, 59 22qDup, and 208 TD control scans; mean interscan interval = 20.27 months) were examined. Longitudinal FreeSurfer analysis pipelines were used to parcellate the cortex and calculate average CT and SA for each region. First, general additive mixed models (GAMMs) were used to identify regions with between-group differences in developmental trajectories. Secondly, we investigated whether these trajectories were associated with clinical outcomes. Developmental trajectories of CT were more protracted in 22qDel relative to TD and 22qDup. 22qDup failed to show normative age-related SA decreases. 22qDel individuals with psychosis spectrum symptoms showed two distinct periods of altered CT trajectories relative to 22qDel without psychotic symptoms. In contrast, 22q11.2 CNV carriers with autism spectrum diagnoses showed early alterations in SA trajectories. Collectively, these results provide new insights into altered neurodevelopment in 22q11.2 CNV carriers, which may shed light on neural mechanisms underlying distinct clinical outcomes.

## Introduction

Genomic imbalances due to copy number variation (CNVs) confer greatly elevated risk for prevalent neurodevelopmental disorders [1, 2], including schizophrenia and autism spectrum disorder (ASD). CNVs present a powerful genetics-first approach to mapping the neurodevelopmental signatures of psychiatric illness.

Reciprocal chromosomal rearrangements at the 22q11.2 locus are particularly compelling models for investigating gene dosage effects on brain maturation and downstream psychiatric outcomes, as this locus contains genes implicated in synapse development and neuronal migration that converge on biological pathways implicated in idiopathic developmental neuropsychiatric conditions [3]. While gene dosage cannot be experimentally manipulated in humans, as in animal or in vitro models, a comparable framework emerges via naturally occurring deletions and duplications at this locus. This “reverse genetics” approach can unpack how genes may influence downstream neurodevelopmental phenotypes [4].

The 22q11.2 deletion (22qDel), also known as Velocardiofacial syndrome (OMIM #188400, #192430), is caused by a 1.5–2.6 Mb hemizygous deletion on the long arm of chromosome 22 [5] and occurs at a rate of ~1 in 4000 live births [6]. 22qDel is among the largest known genetic risk factors for psychotic illness, conferring a 20 to 25-fold increase in risk compared to population base rates [7–9]. 22qDel carriers also have elevated incidence of intellectual disability (ID) as well as high rates of anxiety, attentional deficits, and ASD [10–12].

Less is known about the reciprocal duplication (22qDup), which has only relatively recently been described as a recurrent CNV of clinical interest [13, 14]. Unlike 22qDel, which tends to occur de novo, the duplication is frequently inherited [15, 16]. Available evidence, primarily from case reports [15, 17], indicates that 22qDup is associated with elevated rates of ID/developmental delay [17] and ASD [18], but with incomplete penetrance and highly variable expressivity [19, 20]. Notably, there is emerging evidence that 22qDup is significantly *less* common in schizophrenia cases than in the general population, suggesting the first putative protective mutation for schizophrenia [21–24].

Cross-sectional brain imaging studies of 22qDel have revealed a consistent pattern of cortical alterations involving a rostro-caudal gradient of volumetric reduction [25–27]. More recent investigations have separately investigated cortical thickness (CT) and surface area (SA), as these indices appear to be driven by different genetic mechanisms [28, 29], evolutionary origins [30, 31], and developmental trajectories [32, 33]. In the largest analysis of brain structural alterations in 22qDel to date, 22q11.2-ENIGMA [34] found a widespread pattern of thicker cortex compared to controls. The exception was for cingulate and temporal regions, where focal thinning was observed. There was also a pervasive pattern of lower SA, with largest effects in parieto-occipital and anterior cingulate regions [35].

We recently conducted the first cross-sectional investigation of brain morphology in reciprocal 22q11.2 CNVs, which revealed global opposing effects of 22qDel versus 22qDup [36]. CT and SA were affected in opposite directions, and controls showed an intermediate pattern. Moreover, effects of gene dosage on CT were localized to frontal and parietal regions, whereas cortical SA effects were widespread throughout the cortical mantle, suggesting early neurodevelopmental origins of these alterations. However, longitudinal studies are needed to clearly delineate the differing neurodevelopmental trajectories of reciprocal 22q11.2 CNVs.

A few longitudinal studies, most with limited sample sizes, offer insights into the neuroanatomic trajectory of the 22q11.2 deletion, although findings are somewhat inconsistent across studies (reviewed in [25]). Volumetric studies report either increases [37] or no significant change [38] in grey matter volume with age in frontal regions, in contrast with differential reductions of occipito-parietal regions in 22qDel relative to controls [37]. Longitudinal studies of surface-based measures of CT have reported slower thinning in left parietal regions [39] and accelerated thinning in fronto-temporal regions [40, 41] in 22qDel carriers compared to controls. To our knowledge, only two studies have investigated SA longitudinally in 22qDel, which found consistent SA reductions in 22qDel versus controls [39, 41]. No studies to date have investigated 22qDup carriers longitudinally. Because many developmental processes follow a non-linear trajectory [42], and non-linear modeling approaches in developmental neurocognitive science have identified distinct periods of continued refinement of brain structure in typically-developing youth [43], the use of time-varying approaches may identify distinct periods of change that are obscured in cross-sectional or linear models [44].

Structural brain changes may also be related to clinical outcomes. In 22qDel, psychotic symptoms have been associated with steeper grey matter decline in frontal, temporal, cingulate, and parietal regions during adolescence, a critical period for the emergence of psychosis [37, 38, 45, 46]. Notably, fronto-temporal regions showing greatest thinning in 22qDel patients with psychotic illness significantly overlapped with brain regions most prominently affected in idiopathic psychosis [35, 47]. No study has yet assessed the relationship of longitudinal measures of brain morphology to psychotic and autism symptomatology across 22q11.2 reciprocal CNV carriers.

Here, in the first longitudinal investigation of reciprocal 22q11.2 CNVs, we sought to elucidate neurodevelopmental mechanisms that underlie genetic vulnerability for neuropsychiatric outcomes. We aimed to investigate: (1) developmental trajectories of CT and SA, and (2) whether neuroanatomic trajectories differ as a function of variable psychiatric phenotypes (i.e., psychosis-spectrum, ASD) in the context of these highly penetrant genetic conditions.

## Materials and methods

### Subjects

**Fig. 1:**
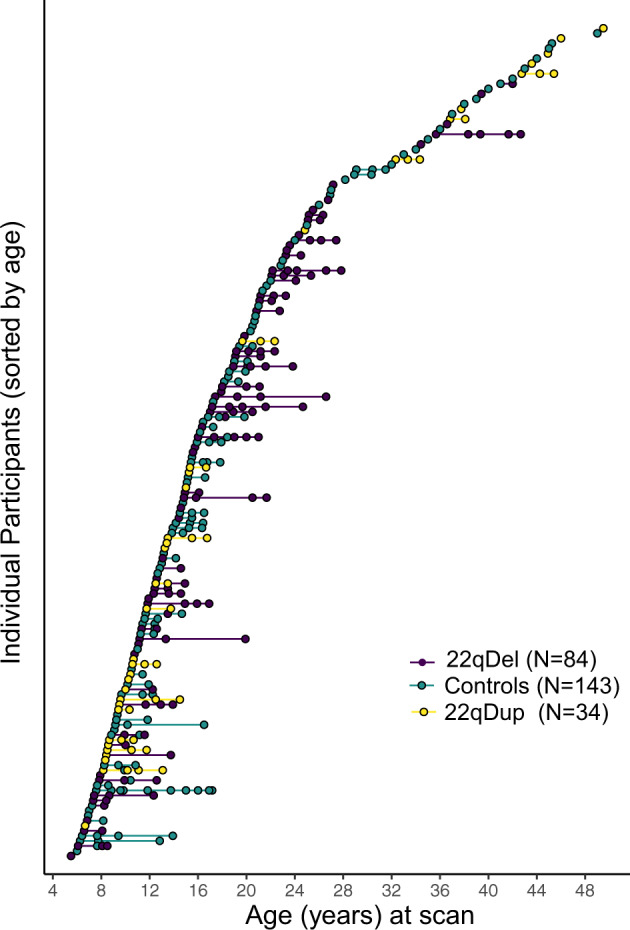
Participant age at each scan. Scan distribution for analyses comparing 22qDel, 22qDup, and TD controls. The sample consisted of 261 participants aged 6–49 years who contributed between 1–6 scans, resulting in a total of 431 scans available for analysis (164 22qDel scans, 59 22qDup scans, and 208 TD control scans).

A total of 261 unique individuals (431 total scans; Fig. 1) were included in the analysis: 84 with molecularly confirmed 22q11.2 deletions, 34 with confirmed 22q11.2 duplications, and 143 demographically-matched typically developing (TD) controls (Table 1). Approximately 54% of the CNV carriers and controls were included in a prior publication on baseline (cross-sectional) data [48]. For participants with multiple scans, the average interscan interval was 20.27(+/−13.16) months. Mean interscan interval did not significantly differ between groups (Table 1). Study participants with multiple timepoints largely did not differ demographically from those with a single timepoint (e-Table 1). Medication information for 22q11.2 CNV carriers is in e-Table 2.

**Table 1:** Participant baseline demographics.

	22q11.2 deletion carriers	Typically-developing controls	22q11.2 duplication carriers
*N*	84	143	34
Age (SD)	17.46 (8.29)	18.96 (10.00)	18.63 (13.71)
Age Range	5.5–42	6–49	6.7–49.5
*N*, females (%)	41 (48.81%)	77 (53.85%)	14 (41.18%)
Race			
*N*, American Indian/Alaska Native (%)	0	7 (4.90%)	0
*N*, Asian (%)	1 (1.19%)	6 (4.20%)	1 (2.94%)
*N*, Black or African American (%)	1 (1.19%)	12 (8.39%)	0
*N*, White (%)	75 (89.29%)	104 (72.73%)	33 (97.06%)
*N*, Multiple Race (%)	7 (8.33%)	14 (9.79%)	0
Parental Education, Years (SD)	15.14 (2.99)	14.31 (3.52)	14.03 (2.47)
*N*, Right-Handed (%)	71 (84.52%)	130 (90.91%)	28 (82.35%)
Full Scale IQ (SD)^a,b,c^	80.01 (12.81)	113.34 (16.53)	95.73 (18.39)
*N*, Psychotic Disorder (%)^a,c^	8 (9.52%)	0	0
*N*, Psychosis-Risk (%)* ^a,b,d^	17 (20.24%)	0	5 (14.71%)
*N*, Autism Spectrum Disorder (ASD), (%)^a,b^	40 (47.62%)	0	14 (41.18%)
Scanner			
*N*, BMC	23	28	0
*N*, CCN	32	102	21
*N*, PRISMA	29	13	13
Mean Interscan Interval, months (SD)	20.52 (12.99)	20.21 (16.04)	16.40 (5.20)

*Psychosis Risk = Attenuated Positive Symptoms.

^a^22q-del ≠CTL (*p* < 0.05).

^b^22q-dup ≠ CTL (*p* < 0.05).

^c^22q-del ≠ 22q-dup (*p* < 0.05).

^d^5 22qDel developed psychosis symptoms post-baseline.

CNV carriers were recruited from either [1] the University of California at Los Angeles or Children’s Hospital, Los Angeles clinics, or [2] local and national support groups and websites. Typically developing (TD) comparison subjects were recruited from the same communities as CNV carriers (see Supplementary Methods for detailed inclusion/exclusion criteria).

All participants underwent a verbal and written informed consent process. Participants under the age of 18 years provided written consent, while their parent or guardian completed written consent after study procedures were explained. Study procedures and informed consent documents were approved by the UCLA Institutional Review Board and performed in accordance with the Declaration of Helsinki. Neurobehavioral phenotyping measures are described in Supplementary Methods.

### Imaging protocol and image processing pipeline

Measures of brain structure were obtained with structural T1-weighted MRI at each timepoint, at the UCLA Brain Mapping Center or at the Center for Cognitive Neuroscience (see Supplementary Methods). Scans were analyzed in an unbiased, whole-brain approach using well-validated publicly available analysis and quality control protocols [49], which have previously been applied by our group and others (e.g, [48–52]. Quality assessment procedures and scanner correction methods are detailed in Supplementary Methods. For our primary analyses, Freesurfer cortical regions of interest (ROI’s) were summed into lobes (frontal, temporal, parietal, occipital) to reduce the number of statistical comparisons (e-Table 3).

### Statistical analyses

#### Aim 1: cortical trajectory analyses

We used general additive mixed models (GAMMs) [53–55] to model group differences (TD vs. 22qDel vs. 22qDup) in the relationship between development (chronological age) and sMRI measures (see Supplementary Methods for details). Because the relationship between the smoothed predictor and the dependent variable is not required to have the same functional form in each group, we examined the smoothed effects of chronological age for 22qDel, 22qDup, and TD separately. Sex, scanner, and estimated total intracranial volume at each timepoint were also included in the models as covariates. To model and account for non-independence of multiple visits, subject was included as a random effect. The dependent variable was the respective sMRI measure being assessed (see Formula 1 in Supplementary Methods). To prevent overfitting, we used restricted maximum likelihood when fitting all GAMMs.

To determine time periods during which age effects in each group differed, we took the difference between the upper and lower 95% confidence intervals of the smoothed fit in two groups, henceforth called the ‘difference in smooths’. For each dependent variable, we considered effects of age to be significantly different in the two groups being compared during periods of time in which the difference in smooths did not include zero. While this approach has been used for other data types [56, 57], to our knowledge this is among the first applications to comparisons of developmental trajectories in neuroimaging data. We also determined time periods in which significant change was occurring in each group (“maturation”; see Supplementary Methods and [43, 44, 58, 59] for details). For any lobar measures that had different age-related trajectories in any group, we conducted post-hoc analyses of the individual ROIs within those lobes (see e-Fig. 1 for analysis flow chart).

#### Aim 2: Relationship of imaging variables to clinical phenotypes

To investigate the relationship of neuroimaging variables to clinical phenotypes, a similar approach was used. Because psychosis spectrum (PS) conditions were only seen in sufficient numbers in the 22qDel group, these analyses compared 22qDel participants with (22qDel-PS+) and without PS symptoms (22qDel-PS−). For this analysis, PS was operationalized as having attenuated or fully psychotic symptoms, based on SIPS interview, at any timepoint. Specifically, we used GAMMs to assess differences in the relationship between development and sMRI measures between 22qDel-PS+ (*N* = 30) and 22qDel-PS− (*N* = 37) (see e-Table 4 and Formula 2 in Supplementary Methods). We also used GAMMs to examine the effects of ASD diagnosis on the relationship between development and sMRI measures in 22qDel and 22qDup separately (e-Tables 5, 6; see Formula 3 in Supplementary Methods). Model set-up was identical to Aim 1, with the respective clinical phenotype included as the group variable (see Supplementary Methods for secondary analyses).

## Results

### Subject characteristics

22q11.2 CNV carriers and TD controls (TD) did not differ in baseline demographic characteristics of sex, age, parental education, handedness, or interscan interval; however, CNV carriers were more likely to be of white (European) ancestry than TD (Table 1). There were also significant group differences in IQ (22qDel < 22qDup < TD), and significantly more psychotic spectrum symptoms in 22qDel vs. 22qDup and TD, whereas rates of ASD were similar across CNV groups, as previously reported [58].

### 22qDel participants exhibit more protracted CT maturation relative to TD and 22qDup

**Fig. 2:**
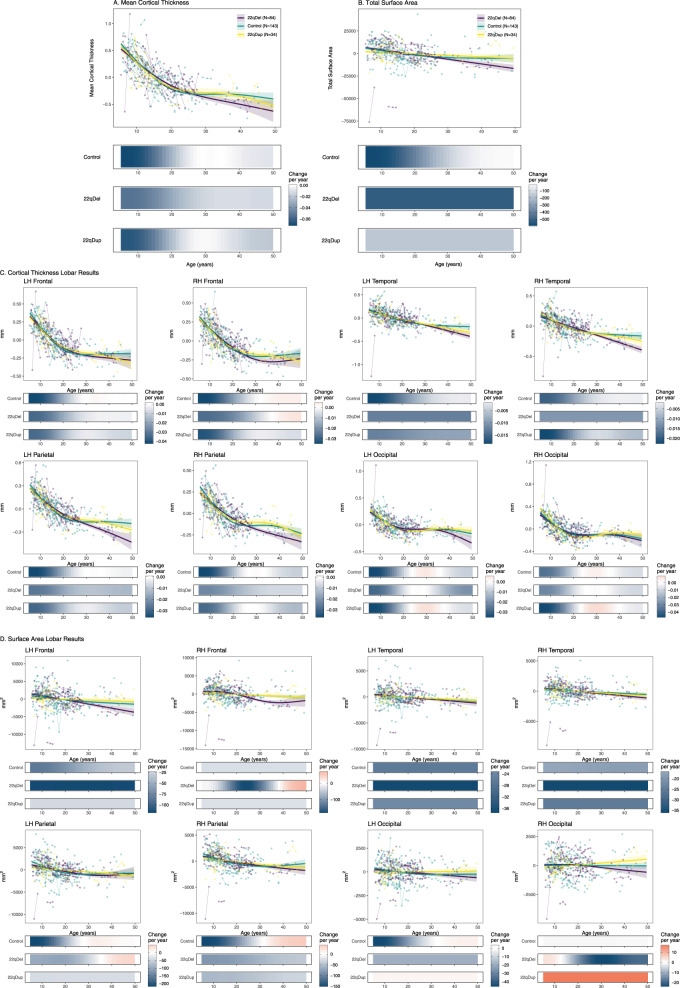
Neurodevelopmental trajectories of cortical thickness and surface area for Controls, 22qDel, and 22qDup. Partial residual plots of **A** mean cortical thickness (CT) trajectories and **B** total surface area (SA) trajectories for Controls (green), 22qDel (purple), and 22qDup (yellow). **C** Lobar values for CT and **D** SA trajectories. The partial residual plots reflect the relationship between age and the respective neuroimaging measures, given the other covariates in the model. Circles reflect an individual participant at a particular visit and participants with longitudinal visits are connected by a straight line. For each group, the thick line reflects the line of best fit and shaded regions are ±standard errors. The bars underneath the age plots reflect the derivative of the slope, i.e., the rate of change taking place at a particular age. Darker blue indicates that there is a stronger decrease in CT or SA taking place at that particular age, while brighter red indicates a stronger increase in CT or SA.

All groups exhibited highly significant cortical thinning with increasing age (Fig. 2a). For most regions, TD and 22qDup showed significant age-related decreases from age 6 through young adulthood (up to age 24). In contrast, 22qDel exhibited an extended period of significant age-related global thinning (up to age 33); a similar developmental pattern was observed for bilateral frontal and right parietal CT. For bilateral temporal and left parietal regions, 22qDel exhibited even more protracted thinning that continued through middle age. Significant occipital thinning was observed from early childhood through young adulthood (6–20 years) across all three groups (Fig. 2c, e-Table 7a).

Difference in smooths revealed a steeper age-related mean CT slope in 22qDel relative to TD, as well as frontal, temporal, and parietal CT slopes between mid-adolescence and early adulthood. For right temporal and bilateral parietal regions, 22qDel also exhibited steeper age-related decline in comparison to TD during mid-adulthood. Bilateral parietal CT slopes also significantly differed between 22qDel and 22qDup during mid-adulthood, with 22qDel showing age-related cortical thinning, after maturation plateaued in TD and 22qDup.

Post-hoc ROI analyses revealed similar patterns of significant difference in smooths, i.e., a steeper age-related slope/smooth from early/mid-adolescence and early adulthood in 22qDel in comparison to TD in the fusiform, inferior and middle temporal, isthmus cingulate, superior parietal, and supramarginal regions bilaterally (e-Table 8).

Lastly, consistent with previous cross-sectional work [35, 48], 22qDel carriers had overall greater CT in comparison to TD and 22qDup, while 22qDup carriers had reduced CT.

### 22qDup fail to show normative age-associated SA decreases

TD and 22qDel exhibited significant age-related decreases in bilateral total and regional SA, whereas 22qDup did not (Fig. 2b, d). While TD exhibited significant age-related declines in total SA from age 6–23, age-related decline in total SA was more protracted in 22qDel, from 6–50 years old (e-Table 7a).

Difference in smooths revealed two periods of significantly steeper age-related change in right frontal SA in 22qDel vs. TD, in early adolescence (10–16 years) and adulthood (24–40 years). A similar pattern was observed for right frontal SA between 22qDel and 22qDup for comparable developmental periods, which was regionally driven by differences in right pars triangularis SA (e-Table 8).In comparison to 22qDup, left parietal SA declined from 6–8 years and 16–22 years in TD, regionally driven by declines in the inferior parietal area. In comparison to 22qDel, right parietal SA declined in early adulthood in TD, regionally driven by declines in postcentral, precuneus, and posterior parietal SA (e-Table 8).

Consistent with our prior cross-sectional findings [35, 48], 22qDel participants had overall lower SA compared to TD and 22qDup participants, whereas 22qDup had greater SA (e-Table 7a).

Results were robust when covarying for IQ and antipsychotic medication usage (e-Tables 9, 10).

### 22qDel-PS+ exhibit two distinct developmental CT trajectories in comparison to 22qDel-PS−

**Fig. 3:**
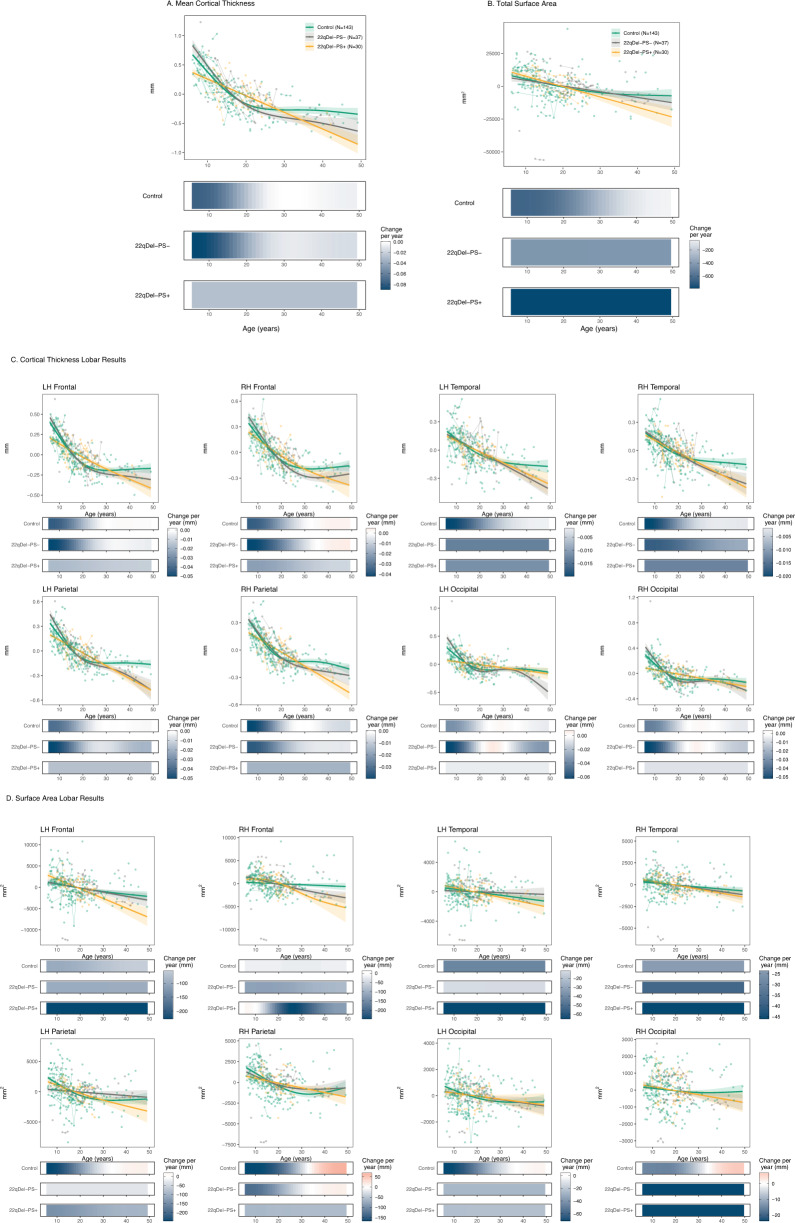
Neurodevelopmental trajectories of cortical thickness and surface area for Controls, 22qDel-PS+, and 22qDel-PS-. Partial residual plots of **A** Mean CT and **B** total SA trajectories for Controls (green), 22qDel-PS− (orange), and 22qDel-PS+(gray). **C** Lobar values for CT and **D** SA trajectories. The partial residual plots reflect the relationship between age and the respective neuroimaging measures, given the other covariates in the model. Circles reflect an individual participant at a particular visit and participants with longitudinal visits are connected by a straight line. For each group, the thick line reflects the line of best fit and shaded regions are ±standard errors. The bars underneath the age plots reflect the derivative of the slope, i.e., the rate of change taking place at a particular age. Darker blue indicates that there is a stronger decrease in CT or SA taking place at that particular age, while brighter red indicates a stronger increase in CT or SA. Control age effects and derivatives are shown for visualization purposes only.

As shown in Fig. 3a, both 22qDel-PS− and 22qDel-PS+ showed significant age-related decreases in mean overall CT, as well as bilateral frontal, temporal and parietal CT, from childhood through adulthood. (Fig. 3c). 22qDel-PS− showed significant occipital thinning bilaterally from 7–20 years old, but 22qDel-PS+ participants did not exhibit significant developmental changes in occipital CT (e-Table 11a).

There were two developmental periods in which age effects differed between 22qDel-PS + vs. 22qDel-PS−. First, 22qDel-PS+ exhibited flatter age-related smooths in mean CT between 7–12 years old in comparison to 22qDel-PS−, observed in left frontal, left parietal, and bilateral occipital lobes (e-Table 11a). Secondly, in comparison to 22qDel-PS−, during late adolescence (from 17–24 years old), 22qDel-PS+ exhibited a steeper age-associated decrease in mean CT, driven by steeper thinning across bilateral frontal and parietal regions (see Supplementary Results and e-Table 12 for individual ROI results).

### 22qDel-PS+ exhibit a more protracted period of age-related SA decline

22qDel-PS− exhibited age-related decline in total SA from 7–30 years old, while 22qDel-PS+ exhibited a more extended period of decline, from 7–43 years old (Fig. 3b). Periods of developmental change for both 22qDel-PS+ and 22qDel-PS− were regionally variable (Fig. 3d).

Two right hemisphere regions had significant differences in developmental trajectories: in right frontal SA, in comparison to 22qDel-PS−, 22qDel-PS+ exhibited a shallower developmental slope from 7–12 years old, but a steeper decline from 17–25 years old. In the right parietal lobe, 22qDel-PS+ had a steeper SA decline in early adulthood, between ages 18–23.

Results for SA and CT remained similar when antipsychotic medication status was included as a covariate, psychosis spectrum status varied as a function of visit, when controlling for comorbid ASD, and when the age range was truncated (7–35 years) due to sparsity of older adult participants (e-Tables 13–16).

### 22q11.2 CNV carriers with ASD exhibit altered SA trajectories relative to CNV carriers without ASD

**Fig. 4:**
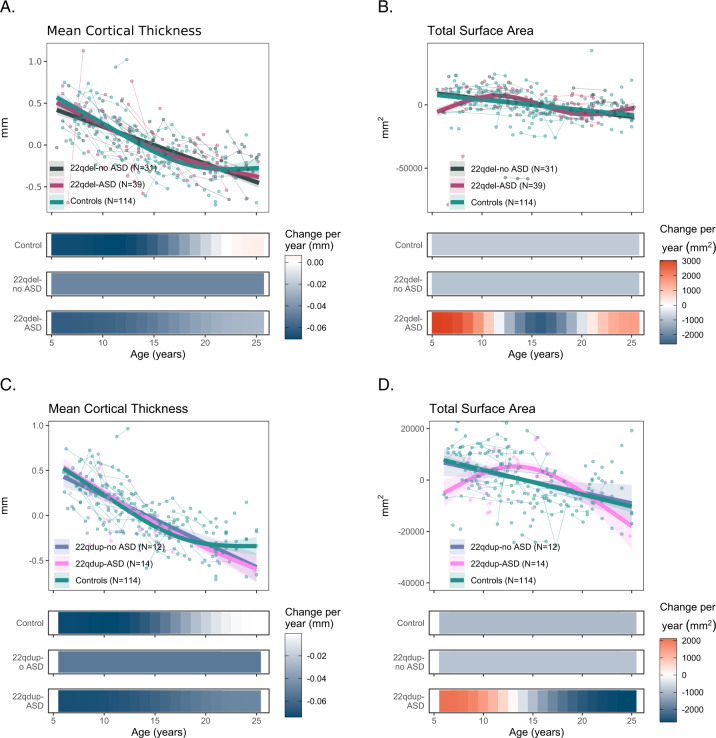
Neurodevelopmental trajectories of mean cortical thickness and total surface area for Controls, individuals with a 22q11 CNV with an ASD diagnosis, and individuals with a 22q11 CNV without an ASD diagnosis. Partial residual plots of **A** Mean CT and **B** total SA trajectories for Controls (green), 22qDel-no ASD (dark blue) and 22qDel-ASD (magenta). **C** Mean CT and **D** total SA trajectories for Controls (green), 22qDup-no ASD (light blue), and 22qDup-ASD (pink). The partial residual plots reflect the relationship between age and the respective neuroimaging measures, given the other covariates in the model. Circles reflect an individual participant at a particular visit and participants with longitudinal visits are connected by a straight line. For each group, the thick line reflects the line of best fit and shaded regions are ±standard errors. The bars underneath the age plots reflect the derivative of the slope, i.e., the rate of change taking place at a particular age. Darker blue indicates that there is a stronger decrease in CT or SA taking place at that particular age, while brighter red indicates a stronger increase in CT or SA. Control age effects and derivatives are shown for visualization purposes only.

Age-related cortical thinning in 22qDel cases with (22qDel-ASD) and without ASD diagnoses (22qDel-no ASD) was similar, and there were no differences in CT trajectories between 22qDel-ASD and 22qDel-no ASD (e-Fig. 2). In contrast, 22qDel cases differed in overall and regional SA as a function of ASD diagnosis (Fig. 4a, b, e-Table 17a, e-Fig. 3). Specifically, 22qDel-ASD had significant overall SA increases from 6–12 years old, while those without an ASD diagnosis showed a linear decrease in SA, similar to TD controls. During adolescence, 22qDel-ASD participants exhibited overall age-related SA decreases, driven by significant bilateral frontal and parietal and right temporal lobe SA decreases. 22qDel-ASD participants showed a significant difference in smooths in comparison to 22qDel-no ASD across childhood and adolescence in total SA, bilateral frontal and parietal SA, and in early adolescence in right temporal SA (e-Tables 17a, 18). Results were similar when comorbid PS was included as a covariate (e-Table 19).

22qDup participants with and without ASD did not show significant differences in overall CT or SA trajectory (Fig. 4c, d), but had a significant difference in smooths in occipital CT bilaterally, involving a steeper age-related slope in 22qDup-ASD in early childhood and adolescence (e-Fig. 4; e-Tables 20, 21). Differences in lobar SA trajectories were not statistically significant in 22qDup-ASD (e-Fig. 5).

All CT results remained consistent when estimated intracranial volume was omitted as a covariate from the model (see e-Tables 7b, 11b, 17b, and 20b).

## Discussion

This is the first study to investigate longitudinal developmental trajectories in reciprocal 22q11.2 CNVs. Our accelerated longitudinal design revealed several novel findings, specifically: (1) 22qDel showed protracted cortical thinning in comparison to TD and 22qDup; (2) although 22qDup CT developmental trajectories were largely intact, 22qDup participants failed to exhibit normative age-related SA decreases; (3) relative to 22qDel individuals without psychotic symptoms, 22qDel with psychosis spectrum symptomatology showed less cortical thinning in childhood, but steeper age-related thinning in adolescence; and (4) 22q11.2 CNV carriers with an autism spectrum diagnosis showed alterations in early SA, rather than global CT, developmental trajectories. To our knowledge, this is the first neuroimaging study to use GAMMs to identify specific developmental epochs where trajectories diverged between groups. Our results thus provide new insights into the timing of neurodevelopmental disruptions resulting from 22q11.2 copy number variation, and how these developmental deviations may be related to clinical phenomena, specifically psychosis and ASD.

### Divergent neurodevelopmental trajectories in 22q11.2 CNV carriers: possible biological mechanisms

Our results in TD controls are highly consistent with prior large-scale longitudinal studies of typical development, which find widespread and regionally variable nonlinear age-associated cortical thinning, with accelerated thinning during adolescence and young adulthood, and comparatively smaller steady decreases in cortical SA [32, 33, 60–62].

We find that brain regions with macroscopic structural abnormalities associated with these CNVs undergo differential rates of growth and decline with age. Specifically, we observed a lack of normative cortical neuromaturation in 22qDel, involving protracted thinning. This aligns with a recent longitudinal study in which, compared to TD controls, participants with 22qDel showed widespread thicker cortex, focal reductions in the cingulate gyrus and superior temporal gyrus (STG), and accelerated cortical thinning during adolescence in frontotemporal and parietal regions [41]. In contrast, we found 22qDup showed a relatively normative thickness trajectory, but a lack of typical age-associated SA decreases.

The abnormal neurodevelopmental trajectories we observed are likely due to multiple neurobiological disruptions. CT partially reflects the number of cells within organizational columns in the neocortex [31]; while age-associated cortical thinning is traditionally believed to be primarily due to glial-cell-dependent synaptic pruning [63, 64], recent evidence suggests that age-associated CT changes may also be driven by white matter maturational processes, i.e. increasing myelination [65]. In contrast, regional SA is thought to be driven by increased progenitor cell production in early development [35]. Notably, the LgDel mouse model of the 22qDel has deficits in intermediate progenitor cell proliferation [66], a possible mechanism for SA dysmaturation. Further, we recently found that SA deviance in 22qDel was associated with expression of 22q11 genes involved in cell proliferation and apoptosis (DGCR8 and AIFM3, respectively) [67], suggesting these genes may contribute to disrupted cortical trajectories in 22q11.2 CNV carriers. However, because age-related SA decreases are consistently observed across child and adolescent development [32, 33], there are likely additional neurobiological and environmental drivers of these changes. Studies of gene dosage effects (i.e., under- vs. over-expression of 22q11.2 genes) in preclinical models are warranted, in conjunction with human studies incorporating multimodal measures of microstructural brain changes and postmortem histology, in order to resolve questions of underlying mechanisms.

### 22qDel with psychosis symptoms show a distinct developmental trajectory

Our finding of divergent trajectories between 22qDel with and without psychotic symptoms suggests that previous cross-sectional findings of lower CT in 22qDel-Psychosis [35] are due to a combination of: (1) earlier reductions that occur during early childhood (or prenatally), and (2) extended cortical thinning from mid-adolescence to early adulthood. Similarly, Bagautdinova et al. [41] recently reported exacerbated cortical thinning in the right STG in 22qDel with psychotic symptoms; while our results instead implicated fronto-parietal regions as driving the abnormal CT trajectory, our studies are consistent—despite differing methodology—in identifying exaggerated cortical thinning in adolescence in 22qDel carriers with psychotic symptomatology. Notably, these findings converge with those in idiopathic (clinical high-risk) youth, in which accelerated gray matter loss, particularly in frontal regions, was observed in those who developed overt psychosis [68].

These findings, in conjunction with other recent work [69–71], suggest that biological factors exert differential influences on behavior at distinct points in development. Failure to progress along normative timetables during childhood (as reflected by flatter trajectories) may put youth with 22qDel at greater risk for developing psychosis, while exaggerated cortical thinning (i.e., steeper age-related slopes) could contribute to psychosis onset in adolescence.

### Effects of ASD on developmental trajectories in 22q11.2 CNV carriers

Notably, ASD diagnosis in 22q11.2 CNV carriers was associated with a different pattern of cortical maturation. In particular, 22qDel with ASD had greater SA increases (relative to 22qDel without ASD), particularly during childhood. While 22qDup was associated with similarly high rates of ASD, this early SA increase was observed visually, but was not statistically significant. Given the modest N in this group, however, we may have had insufficient power to detect this developmental alteration. These patterns are broadly consistent with findings of early brain overgrowth in idiopathic ASD, followed by accelerated decline [72]. However, findings in idiopathic ASD appear driven by anomalous CT development, rather than SA [73]. One possibility is that 22q11.2-associated ASD diagnosis is associated with *accelerated* SA growth patterns, akin to the acceleration-deceleration hypothesis of chronic stress and neurodevelopment [74].

### Limitations

Several limitations of the study should be noted. In particular, we had a sparser distribution in older ages (>35 years old), so caution is warranted in interpreting results for these age epochs. We limited analyses of psychiatric diagnosis to age ranges in which there were a sufficient number of individuals, to ensure robustness of results. Nevertheless, additional longitudinal data points, particularly in older subjects, are required to model growth curves at the single-subject level. Secondly, the sample size of 22qDup was also limited, and the distribution is sparse in the older ages. In addition, we applied longitudinal ComBat correction to adjust for scanner effects, which may have been overly stringent in removing non-linear effects. These limitations may have precluded us from being able to detect significant neurodevelopmental trajectories in surface area in the 22qDup sample. However, at present the 22qDup sample is the only cohort of its kind. We will be able to extend upon our findings with multisite studies that are now underway. Additionally, like other nonparametric approaches, GAMM is prone to overfitting and is sensitive to outliers. However, we attempted to protect against this limitation by limiting the number of splines that can occur when the line of best fit is determined, and we used restricted maximum likelihood to optimize the smoothness fit. It is also possible that the harmonization method we used to remove site effects limited our ability to detect more subtle non-linear age-related deviations. Thus, future studies comparing alternative harmonization techniques (e.g, [75] are warranted.

## Conclusions

Our study provides new insights into effects of 22q11.2 gene dosage on neurodevelopment; findings of disrupted longitudinal brain trajectories are notable, given the opposing brain phenotypes of these neuropsychiatric CNVs, and differential association with psychosis risk of the 22qDel versus 22qDup. Biological mechanisms underlying the observed differential impact of 22q11.2 gene dosage on neuromaturational trajectories are currently unknown, and warrant investigation of cell-type specific effects in animal and in vitro models.

## Supplementary information

SUPPLEMENTAL Material- Methods and Resuls

Supplemental Tables

Supplementary Figures

## References

[1] Hoeffding LK, Pedersen CB, Werge T (2017). Lessons to be Learned From 22q2.11 Syndromes-Reply. JAMA Psychiatry.

[2] Malhotra D, Sebat J (2012). CNVs: harbingers of a rare variant revolution in psychiatric genetics. Cell.

[3] Forsyth JK, Nachun D, Gandal MJ, Geschwind DH, Anderson AE, Coppola G (2020). Synaptic and Gene Regulatory Mechanisms in Schizophrenia, Autism, and 22q11.2 Copy Number Variant–Mediated Risk for Neuropsychiatric Disorders. Biol Psychiatry.

[4] Hiroi N, Takahashi T, Hishimoto A, Izumi T, Boku S, Hiramoto T (2013). Copy number variation at 22q11.2: from rare variants to common mechanisms of developmental neuropsychiatric disorders. Mol Psychiatry.

[5] Shaikh TH, O’Connor RJ, Pierpont ME, McGrath J, Hacker AM, Nimmakayalu M (2007). Low copy repeats mediate distal chromosome 22q11.2 deletions: sequence analysis predicts breakpoint mechanisms. Genome Res.

[6] McDonald-McGinn DM, Sullivan KE, Marino B, Philip N, Swillen A, Vorstman JAS (2015). 22q11.2 deletion syndrome. Nat Rev Dis Prim.

[7] Bassett AS, Chow EWC (2008). Schizophrenia and 22q11.2 Deletion Syndrome. Curr Psychiatry Rep.

[8] Green T, Gothelf D, Glaser B, Debbane M, Frisch A, Kotler M (2009). Psychiatric disorders and intellectual functioning throughout development in velocardiofacial (22q11.2 deletion) syndrome. J Am Acad Child Adolesc Psychiatry.

[9] Schneider M, Debbané M, Bassett AS, Chow EWC, Fung WLA, van den Bree MBM (2014). Psychiatric Disorders From Childhood to Adulthood in 22q11.2 Deletion Syndrome: Results From the International Consortium on Brain and Behavior in 22q11.2 Deletion Syndrome. Am J Psychiatry.

[10] Niklasson L, Rasmussen P, Oskarsdóttir S, Gillberg C (2001). Neuropsychiatric disorders in the 22q11 deletion syndrome. Genet Med.

[11] Niklasson L, Rasmussen P, Oskarsdóttir S (2009). Gillberg C. Autism, ADHD, mental retardation and behavior problems in 100 individuals with 22q11 deletion syndrome. Res Dev Disabil.

[12] Girirajan S, Brkanac Z, Coe BP, Baker C, Vives L, Vu TH, et al. Relative Burden of Large CNVs on a Range of Neurodevelopmental Phenotypes. PLoS Genet. 2011. Accessed 2021 Jan 8. https://www.ncbi.nlm.nih.gov/pmc/articles/PMC3213131/. 10.1371/journal.pgen.1002334 PMC3213131 22102821

[13] Ensenauer RE, Adeyinka A, Flynn HC, Michels VV, Lindor NM, Dawson DB (2003). Microduplication 22q11.2, an emerging syndrome: clinical, cytogenetic, and molecular analysis of thirteen patients. Am J Hum Genet.

[14] Portnoï M-F (2009). Microduplication 22q11.2: A new chromosomal syndrome. Eur J Med Genet.

[15] Ou Z, Berg JS, Yonath H, Enciso VB, Miller DT, Picker J (2008). Microduplications of 22q11.2 are frequently inherited and are associated with variable phenotypes. Genet Med.

[16] Olsen L, Sparsø T, Weinsheimer SM, Dos Santos MBQ, Mazin W, Rosengren A (2018). Rearrangements in the 22q11.2 Region: Prevalence and Population-Based Risk for Neuropsychiatric and Developmental Disorders. Lancet Psychiatry.

[17] Wentzel C, Fernström M, Ohrner Y, Annerén G, Thuresson A-C (2008). Clinical variability of the 22q11.2 duplication syndrome. Eur J Med Genet.

[18] Wenger TL, Miller JS, DePolo LM, de Marchena AB, Clements CC, Emanuel BS (2016). 22q11.2 duplication syndrome: elevated rate of autism spectrum disorder and need for medical screening. Mol Autism.

[19] Chawner SJRA, Owen MJ, Holmans P, Raymond FL, Skuse D, Hall J (2019). Genotype-phenotype associations in children with copy number variants associated with high neuropsychiatric risk in the UK (IMAGINE-ID): a case-control cohort study. Lancet Psychiatry.

[20] Kendall KM, Rees E, Bracher-Smith M, Legge S, Riglin L, Zammit S (2019). Association of Rare Copy Number Variants With Risk of Depression. JAMA Psychiatry.

[21] Li Z, Chen J, Xu Y, Yi Q, Ji W, Wang P (2016). Genome-wide Analysis of the Role of Copy Number Variation in Schizophrenia Risk in Chinese. Biol Psychiatry.

[22] Marshall CR, Howrigan DP, Merico D, Thiruvahindrapuram B, Wu W, Greer DS (2017). Contribution of copy number variants to schizophrenia from a genome-wide study of 41,321 subjects. Nat Genet.

[23] Rees E, Kirov G, Sanders A, Walters JTR, Chambert KD, Shi J (2014). Evidence that duplications of 22q11.2 protect against schizophrenia. Mol Psychiatry.

[24] Rees E, Kendall K, Pardiñas AF, Legge SE, Pocklington A, Escott-Price V (2016). Analysis of Intellectual Disability Copy Number Variants for Association With Schizophrenia. JAMA Psychiatry.

[25] Jalbrzikowski M (2021). Neuroimaging Phenotypes Associated With Risk and Resilience for Psychosis and Autism Spectrum Disorders in 22q11.2 Microdeletion Syndrome. Biol Psychiatry Cogn Neurosci Neuroimaging.

[26] Rogdaki M, Gudbrandsen M, McCutcheon RA, Blackmore CE, Brugger S, Ecker C (2020). Magnitude and heterogeneity of brain structural abnormalities in 22q11.2 deletion syndrome: a meta-analysis. Mol Psychiatry.

[27] Scarpazza C, Lattanzi GM, Antoniades M, Di Fabio F, Sartori G, Eickhoff SB (2019). Systematic review and multi-modal meta-analysis of magnetic resonance imaging findings in 22q11.2 deletion syndrome: Is more evidence needed?. Neurosci Biobehav Rev.

[28] Kremen WS, Fennema‐Notestine C, Eyler LT, Panizzon MS, Chen C-H, Franz CE (2013). Genetics of brain structure: Contributions from the vietnam era twin study of aging. Am J Med Genet Part B.

[29] Winkler AM, Kochunov P, Blangero J, Almasy L, Zilles K, Fox PT (2010). Cortical Thickness or Grey Matter Volume? The Importance of Selecting the Phenotype for Imaging Genetics Studies. Neuroimage.

[30] Panizzon MS, Fennema-Notestine C, Eyler LT, Jernigan TL, Prom-Wormley E, Neale M (2009). Distinct Genetic Influences on Cortical Surface Area and Cortical Thickness. Cereb Cortex.

[31] Rakic P (1995). A small step for the cell, a giant leap for mankind: a hypothesis of neocortical expansion during evolution. Trends Neurosci.

[32] Raznahan A, Shaw P, Lalonde F, Stockman M, Wallace GL, Greenstein D (2011). How Does Your Cortex Grow?. J Neurosci.

[33] Wierenga LM, Langen M, Oranje B, Durston S (2014). Unique developmental trajectories of cortical thickness and surface area. NeuroImage.

[34] ENIGMA. Accessed 2021 Feb 10. http://enigma.ini.usc.edu/.

[35] Sun Z, Williams DJ, Xu B, Gogos JA (2018). Altered function and maturation of primary cortical neurons from a 22q11.2 deletion mouse model of schizophrenia. Transl Psychiatry.

[36] Lin A, Ching CRK, Vajdi A, Sun D, Jonas RK, Jalbrzikowski M (2017). Mapping 22q11.2 Gene Dosage Effects on Brain Morphometry. J Neurosci.

[37] Radoeva PD, Bansal R, Antshel KM, Fremont W, Peterson BS, Kates WR (2017). Longitudinal study of cerebral surface morphology in youth with 22q11.2 deletion syndrome, and association with positive symptoms of psychosis. J Child Psychol Psychiatry.

[38] Kates WR, Antshel KM, Faraone SV, Fremont WP, Higgins AM, Shprintzen RJ (2011). Neuroanatomic Predictors to Prodromal Psychosis in Velocardiofacial Syndrome (22q11.2 Deletion Syndrome): A Longitudinal Study. Biol Psychiatry.

[39] Ramanathan S, Mattiaccio LM, Coman IL, Botti J-AC, Fremont W, Faraone SV (2017). Longitudinal trajectories of cortical thickness as a biomarker for psychosis in individuals with 22q11.2 deletion syndrome. Schizophr Res.

[40] Schaer M, Debbané M, Bach Cuadra M, Ottet M-C, Glaser B, Thiran J-P (2009). Deviant trajectories of cortical maturation in 22q11.2 deletion syndrome (22q11DS): a cross-sectional and longitudinal study. Schizophr Res.

[41] Bagautdinova J, Zöller D, Schaer M, Padula MC, Mancini V, Schneider M (2021). Altered cortical thickness development in 22q11.2 deletion syndrome and association with psychotic symptoms. Mol Psychiatry.

[42] Zhou D, Lebel C, Treit S, Evans A, Beaulieu C (2015). Accelerated longitudinal cortical thinning in adolescence. NeuroImage.

[43] Calabro FJ, Murty VP, Jalbrzikowski M, Tervo-Clemmens B, Luna B (2020). Development of Hippocampal–Prefrontal Cortex Interactions through Adolescence. Cereb Cortex.

[44] Bridgwater M, Bachman P, Tervo-Clemmens B, Haas G, Hayes R, Luna B (2022). Developmental influences on symptom expression in antipsychotic-naïve first-episode psychosis. Psychol Med.

[45] Gothelf D, Hoeft F, Ueno T, Sugiura L, Lee AD, Thompson P (2011). Developmental changes in multivariate neuroanatomical patterns that predict risk for psychosis in 22q11.2 deletion syndrome. J Psychiatr Res.

[46] Padula MC, Schaer M, Armando M, Sandini C, Zöller D, Scariati E (2018). Cortical morphology development in patients with 22q11.2 deletion syndrome at ultra-high risk of psychosis. Psychological Med.

[47] van Erp TGM, Walton E, Hibar DP, Schmaal L, Jiang W, Glahn DC (2018). Cortical Brain Abnormalities in 4474 Individuals With Schizophrenia and 5098 Control Subjects via the Enhancing Neuro Imaging Genetics Through Meta Analysis (ENIGMA) Consortium. Biol Psychiatry.

[48] Lin A, Ching CRK, Vajdi A, Sun D, Jonas RK, Jalbrzikowski M (2017). Mapping 22q11.2 Gene Dosage Effects on Brain Morphometry. J Neurosci.

[49] Thompson PM, Stein JL, Medland SE, Hibar DP, Vasquez AA, Renteria ME (2014). The ENIGMA Consortium: large-scale collaborative analyses of neuroimaging and genetic data. Brain Imaging Behav.

[50] Ching CRK, Gutman BA, Sun D, Villalon Reina J, Ragothaman A, Isaev D (2020). Mapping Subcortical Brain Alterations in 22q11.2 Deletion Syndrome: Effects of Deletion Size and Convergence With Idiopathic Neuropsychiatric Illness. AJP.

[51] Imaging Protocols « ENIGMA. Accessed 2021 Feb 10. http://enigma.ini.usc.edu/protocols/imaging-protocols/.

[52] Beer JC, Tustison NJ, Cook PA, Davatzikos C, Sheline YI, Shinohara RT (2020). Longitudinal ComBat: A method for harmonizing longitudinal multi-scanner imaging data. NeuroImage.

[53] Hastie T, Tibshirani R (1986). Generalized Additive Models. Stat Sci.

[54] Wood SN (2004). Stable and Efficient Multiple Smoothing Parameter Estimation for Generalized Additive Models. J Am Stat Assoc.

[55] Wood SN (2011). Fast stable restricted maximum likelihood and marginal likelihood estimation of semiparametric generalized linear models. J R Stat Soc.

[56] Anas MUM, Simpson GL, Leavitt PR, Cumming BF, Laird KR, Scott KA (2019). Taxon-specific variation in δ13C and δ15N of subfossil invertebrate remains: Insights into historical trophodynamics in lake food-webs. Ecol Indic.

[57] Rose NL, Yang H, Turner SD, Simpson GL (2012). An assessment of the mechanisms for the transfer of lead and mercury from atmospherically contaminated organic soils to lake sediments with particular reference to Scotland, UK. Geochimica et Cosmochimica Acta.

[58] Lin A, Vajdi A, Kushan-Wells L, Helleman G, Hansen LP, Jonas RK (2020). Reciprocal Copy Number Variations at 22q11.2 Produce Distinct and Convergent Neurobehavioral Impairments Relevant for Schizophrenia and Autism Spectrum Disorder. Biol Psychiatry.

[59] Wood SN. Generalized Additive Models: An Introduction with R, Second Edition. Boca Raton, FL: CRC Press; 2017. p. 497.

[60] Vijayakumar N, Allen NB, Youssef G, Dennison M, Yücel M, Simmons JG (2016). Brain development during adolescence: A mixed-longitudinal investigation of cortical thickness, surface area, and volume. Hum Brain Mapp.

[61] Tamnes CK, Herting MM, Goddings A-L, Meuwese R, Blakemore S-J, Dahl RE (2017). Development of the Cerebral Cortex across Adolescence: A Multisample Study of Inter-Related Longitudinal Changes in Cortical Volume, Surface Area, and Thickness. J Neurosci.

[62] Sowell ER, Thompson PM, Leonard CM, Welcome SE, Kan E, Toga AW (2004). Longitudinal mapping of cortical thickness and brain growth in normal children. J Neurosci.

[63] Huttenlocher PR (1979). Synaptic density in human frontal cortex - developmental changes and effects of aging. Brain Res.

[64] Rakic P, Bourgeois J-P, Eckenhoff MF, Zecevic N, Goldman-Rakic PS (1986). Concurrent Overproduction of Synapses in Diverse Regions of the Primate Cerebral Cortex. Science.

[65] Natu VS, Gomez J, Barnett M, Jeska B, Kirilina E, Jaeger C (2019). Apparent thinning of human visual cortex during childhood is associated with myelination. PNAS.

[66] Paronett EM, Meechan DW, Karpinski BA, LaMantia A-S, Maynard TM (2015). Ranbp1, Deleted in DiGeorge/22q11.2 Deletion Syndrome, is a Microcephaly Gene That Selectively Disrupts Layer 2/3 Cortical Projection Neuron Generation. Cereb Cortex.

[67] Forsyth JK, Mennigen E, Lin A, Sun D, Vajdi A, Kushan-Wells L (2021). Prioritizing Genetic Contributors to Cortical Alterations in 22q11.2 Deletion Syndrome Using Imaging Transcriptomics. Cereb Cortex.

[68] Cannon TD, Chung Y, He G, Sun D, Jacobson A, van Erp TGM (2015). Progressive reduction in cortical thickness as psychosis develops: a multisite longitudinal neuroimaging study of youth at elevated clinical risk. Biol Psychiatry.

[69] Jalbrzikowski M, Hayes RA, Scully KE, Franzen PL, Hasler BP, Siegle GJ, et al. Associations between brain structure and sleep patterns across adolescent development. Sleep. 2021. Accessed 2021 Sep 11;(zsab120). 10.1093/sleep/zsab120. 10.1093/sleep/zsab120 PMC8503824 33971013

[70] Jalbrzikowski M, Larsen B, Hallquist MN, Foran W, Calabro F, Luna B (2017). Development of White Matter Microstructure and Intrinsic Functional Connectivity Between the Amygdala and Ventromedial Prefrontal Cortex: Associations With Anxiety and Depression. Biol Psychiatry.

[71] Jalbrzikowski M, Hayes RA, Wood SJ, Nordholm D, Zhou JH, Fusar-Poli P (2021). Association of Structural Magnetic Resonance Imaging Measures With Psychosis Onset in Individuals at Clinical High Risk for Developing Psychosis: An ENIGMA Working Group Mega-analysis. AMA Psychiatry.

[72] Courchesne E, Karns CM, Davis HR, Ziccardi R, Carper RA, Tigue ZD (2001). Unusual brain growth patterns in early life in patients with autistic disorder: an MRI study. Neurology.

[73] Raznahan A, Toro R, Daly E, Robertson D, Murphy C, Deeley Q (2010). Cortical anatomy in autism spectrum disorder: an in vivo MRI study on the effect of age. Cereb Cortex.

[74] Tottenham N, Sheridan M (2010). A review of adversity, the amygdala and the hippocampus: a consideration of developmental timing. Front Hum Neurosci.

[75] Pomponio R, Erus G, Habes M, Doshi J, Srinivasan D, Mamourian E (2020). Harmonization of large MRI datasets for the analysis of brain imaging patterns throughout the lifespan. NeuroImage.

