# Supplementary material for: Longitudinal trajectories of cortical development in 22q11.2 copy number variants and typically developing controls
Source: Mol Psychiatry. 2022 Jul 27;27(10):4181–90. doi: 10.1038/s41380-022-01681-w (PMC9718681; doi:10.1038/s41380-022-01681-w)
Supplement: Supplementary file 1 — SUPPLEMENTAL Material- Methods and Resuls [file 41380_2022_1681_MOESM1_ESM.docx]

SUPPLEMENTAL MATERIAL

***Supplemental Methods***

*Detailed inclusion/exclusion criteria*

All 22q11.2 CNV carriers had molecularly confirmed 22q11.2 deletions or duplications. Typically developing (TD) comparison subjects were recruited from the same communities as CNV carriers via online advertisements and by posting flyers and brochures at local schools, pediatric clinics, and other community sites.

Exclusion criteria for all study participants included significant neurological or medical conditions (unrelated to 22q11.2 CNV) that might affect brain structure, history of head injury with loss of consciousness, insufficient fluency in English, and/or substance or alcohol abuse or dependence within the past 6 months. TD controls could not have significant intellectual disability or meet criteria for any major mental disorder. We aimed to include as representative a cohort of CNV carriers as possible, and thus did not exclude participants for medical conditions such as cardiac-related issues, which are common in 22q11.2 deletion carriers. Control participants were excluded if they had intellectual disability (IQ<70), evidence of past or current major mental disorder, based on information gathered during the Structured Clinical Interview for the Diagnostic and Statistical Manual of Mental Disorders, edition 4 (SCID-4; [(1)](https://www.zotero.org/google-docs/?okFZ2k) and/or Computerized Diagnostic Interview for Children (C-DISC; [(2)](https://www.zotero.org/google-docs/?kwiXoj), and/or first-degree relative with a psychotic disorder diagnosis. As attention-deficit/hyperactivity disorder (ADHD) is a common childhood behavioral disorder [(3)](https://www.zotero.org/google-docs/?60GRGV), this was not exclusionary. Training, reliability, and quality assurance procedures for clinical assessments and diagnostic procedures are described in prior publications [(4,5)](https://www.zotero.org/google-docs/?rATV3e).

*Neurobehavioral phenotyping: Clinical and Cognitive Assessments*

In participants aged 10 and up, the positive symptom items from the Structured Interview for Psychosis-Risk Syndromes (SIPS) [(6)](https://www.zotero.org/google-docs/?ygm45Z) were assessed at each timepoint to capture dimensional psychosis-relevant traits (unusual thought content/delusional ideas, suspiciousness/persecutory ideas, perceptual abnormalities/hallucinations, grandiosity, and disorganized communication). Similarly, social and role functioning were assessed at each timepoint using the Global Functioning Scales: Social and Role [(7)](https://www.zotero.org/google-docs/?KFAsAa) ASD diagnosis was assessed at study entry; in children, via the Autism Diagnostic Observation Schedule (ADOS) [(8)](https://www.zotero.org/google-docs/?SA3Um1) and Autism Diagnostic Interview-Revised (ADI-R) [(9)](https://www.zotero.org/google-docs/?3YLu3p) and in adult participants via SCID interview [(10)](https://www.zotero.org/google-docs/?OsX0kS) with an additional developmental disorders module [(10)](https://www.zotero.org/google-docs/?QWfKio), based on DSM-IV/V criteria [(11)](https://www.zotero.org/google-docs/?tkquzQ) (for details, see [(12)](https://www.zotero.org/google-docs/?MIZGdt)). Measures of global cognitive function were assessed at each timepoint, including Full-Scale, Verbal IQ, and Nonverbal IQ (i.e. Matrix Reasoning) using the Wechsler Abbreviated Scale of Intelligence 2nd Edition (WASI-2 [(13)](https://www.zotero.org/google-docs/?AQpOMg) or Wechsler Adult Intelligence Scale, Ed. 4 [(14)](https://www.zotero.org/google-docs/?il7mEJ)*.*

*Scanning Protocol*

All scans were acquired at UCLA, using an identical protocol on a 3 Tesla Siemens Tim Trio MRI scanner at the UCLA Brain Mapping Center or at the Center for Cognitive Neuroscience (12-channel head coil), on which scans were acquired from 2008 -2016, or on a 3T Siemens Prisma scanner (32-channel head coil), which was used from 2017-2020. The combination of different scanners is expected for large longitudinal cohorts such as this; efforts were taken to ensure comparability of scans, both in terms of the acquisition parameters and post-acquisition processing (see Image Pre-Processing and Analysis, below). Each scan began with a 10 min acquisition to determine regional anatomy, including a sagittal localizer image (TR/TE = 500/33 ms, 192 × 256 matrix), a high-resolution T2-weighted axial image (TR/TE = 5000/33 ms, 128 × 128 matrix, FOV = 200 × 200 mm), and a sagittal 1 mm3 T1-weighted image. We used FreeSurfer to process 1 mm3 T1-weighted anatomical images acquired with an MPRAGE sequence. The parameters for the MPRAGE on the Siemens Tim Trio were the following: TR = 2.3 s, TE = 2.91 ms, FOV = 256 mm, matrix = 240 × 256, flip angle = 9°, slice thickness = 1.20 mm, 160 slices. Prisma MPRAGE acquisition parameters were almost identical: TR = 2.3 s, TE = 2.94 ms, FOV = 256 mm, matrix = 240 × 256, flip angle = 9°, slice thickness = 1.20 mm, 160 slices.

*Imaging Pre-Processing and Quality Control*

The FreeSurfer image analysis suite (version 5.3.0; [(15)](https://www.zotero.org/google-docs/?CUOIjb)) surface-based processing pipeline was used to derive measures of volume, cortical thickness, and surface area. FreeSurfer is a well-validated processing package that has been previously described in detail [(16,17)](https://www.zotero.org/google-docs/?kLULf4). We extracted cortical measures based on the Desikan FreeSurfer atlas [(18)](https://www.zotero.org/google-docs/?lA1pbI). T1-weighted MRI scans were analyzed using an unbiased, whole-brain approach with well-validated analysis and quality control protocols developed for the ENIGMA consortium [(19)](https://www.zotero.org/google-docs/?IK6SeV), which have been applied in previous large-scale imaging studies (e.g., [(20)](https://www.zotero.org/google-docs/?vyD0ZU) including our multisite study of 22qDel [(21)](https://www.zotero.org/google-docs/?uI3nQV). Using the ENIGMA quality assessment pipeline [(22)](https://www.zotero.org/google-docs/?PdVKSl), segmented regions were visually inspected and statistically evaluated for outliers following standardized protocols [(23)](https://www.zotero.org/google-docs/?KENMrN), as described in [(3)](https://www.zotero.org/google-docs/?Ytr9AC). Visual inspection of scans was completed by 2 individuals (AL, AV) blind to diagnostic status. Based on these protocols, 12 scans (8 unique subjects) in the control group, 64 scans (30 unique subjects) in the deletion group, and 22 scans (13 unique subjects) in the duplication group failed quality control and were excluded from subsequent analyses. The relatively greater number of exclusions in the CNV groups were all due to excessive motion, which rendered the scan of insufficient quality for analysis.

After undergoing QC, images were then processed with the longitudinal stream in FreeSurfer [(15,24)](https://www.zotero.org/google-docs/?5R3mnn). Specifically, unbiased within-subject templates were created using robust, inverse consistent registration [(25)](https://www.zotero.org/google-docs/?jKf4mc). Several processing steps, such as skull stripping, Talairach transforms, atlas registration as well as spherical surface maps and parcellations were then initialized with common information from the within-subject template, which significantly improves reliability and statistical power [(24)](https://www.zotero.org/google-docs/?8cGgM9). For our primary analyses, Freesurfer cortical ROI’s were summed into lobes (frontal, temporal, parietal, occipital; **see e-Table 3)** to reduce the number of statistical comparisons conducted.

*Post-processing scan harmonization*

To harmonize data acquired from different scanners (2 Tim Trio scanners and 1 Prisma), prior to performing statistical analyses we applied longitudinal ComBat to our data output [(26)](https://www.zotero.org/google-docs/?F5dxjQ), a method originally developed for genomics data and subsequently adapted for neuroimaging data [(27)](https://www.zotero.org/google-docs/?dkdGet). ComBat has shown to be an effective harmonization technique that removes unwanted variation associated with site and scanner, while preserving biological associations in the data [(27)](https://www.zotero.org/google-docs/?Ktu0Ex). The new longitudinal adaptation, which accounts for within-subject repeated measures [(26)](https://www.zotero.org/google-docs/?tSIrTs), has recently been shown to be more powerful for detecting longitudinal change than cross-sectional ComBat, and to control type I error rate better than unharmonized data adjusting for scanner. R code is available at <https://github.com/pittnerdlab/22q11_longitudinal_cortical_sMRI>.

*Statistical Analyses*

General additive mixed models (GAMMs) were used to model group differences. A GAMM is an extension of the general linear mixed effects model but does not assume a linear relationship between the predictor and dependent variable, allowing for a more flexible relationship. GAMMs assess a penalty on nonlinearity in order to avoid overfitting and allow for structured errors, such as those observed in longitudinal studies. Smoothed predictor functions are automatically derived during model estimation with basis functions.

To determine time periods in which significant change was occurring in each group (“maturation”), we used a multivariate normal distribution whose vector of means and covariance were defined by the fitted GAMM parameters to simulate 10,000 GAMM fits and their first derivatives, generated at 0.1 year age intervals. Similar to previous publications [(28–30)](https://www.zotero.org/google-docs/?wU4rOk) and in line with recent guidelines [(31)](https://www.zotero.org/google-docs/?zmsim6), significant intervals of age-related change in sMRI measures were defined as ages when the 95% confidence intervals of simulated GAMM fits did not include zero.

Like other non-parametric approaches, GAMMs may be sensitive to outliers; we attempted to protect against this limitation by limiting the number of splines that can occur when the line of best fit is determined, and we used restricted maximum likelihood to optimize the smoothness fit.

Analyses utilized the below formulas:

Formula 1:


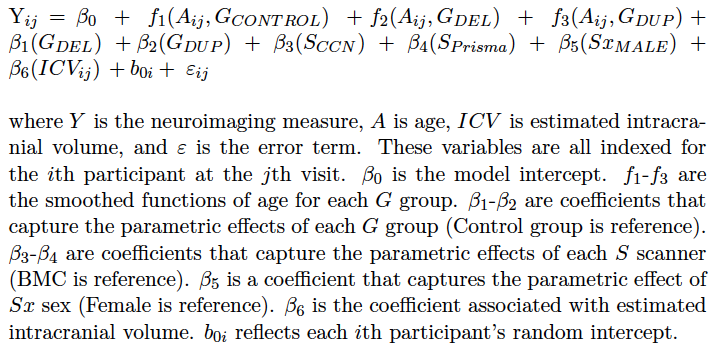


Formula 2:


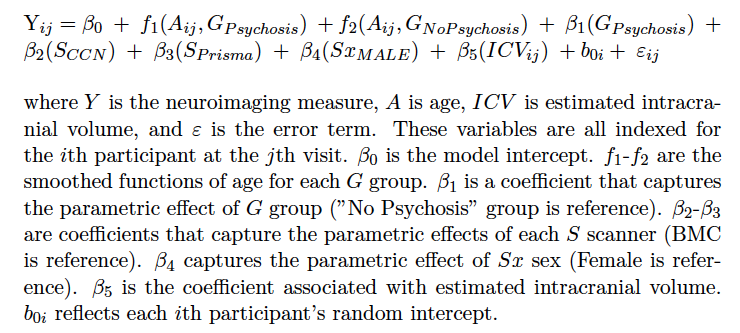


Formula 3:


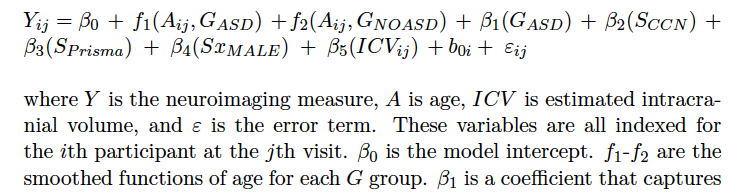


*
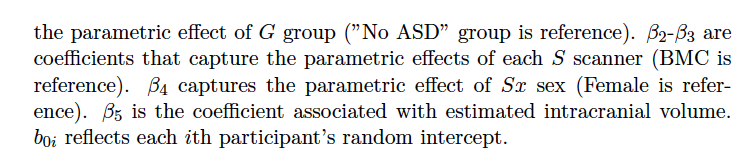
*

*Secondary Analyses, Aim 1. Overall Group Trajectories.* GAMM models were repeated, adjusting for IQ and antipsychotic medication usage.

*Aim 2. Psychosis Spectrum Trajectory Secondary /Sensitivity Analyses in 22qDel.* GAMM models were repeated covarying for antipsychotic medication use and comorbid ASD, treating psychosis spectrum status as a time-varying variable, and truncating the age range to 35 and under (given sparse distribution of subjects in older age range).

*Aim 2: ASD Trajectory Analyses in 22q11.2 CNV Carriers.* Because the number of CNV carriers 26-50 years old with an available ASD diagnosis was sparse (22qDel=5; 22qDup N=7), we restricted all ASD-relevant analyses to participants 25 years and younger. Secondary analyses were conducted in 22qDel with and without ASD covarying for comorbid psychosis spectrum symptoms.

***Supplemental Results***

*Demographic Comparison for Participants with Single vs. Multiple Timepoints*

For participants with a single timepoint, follow-up data were unavailable due to a variety of reasons, most commonly technical (i.e., dental braces) or social reasons (subject moved/family unable to return for follow-up). Demographics largely did not differ between study participants with single vs. multiple timepoints (**e-Table 1**).

*Supplemental Results: Region of Interest Analyses*

*Comparison of 22qDel with (PS+) vs. without Psychosis Spectrum Symptoms (PS -)*

The shallower age-related slope in left frontal CT in 22qDel-PS+ was observed regionally in the frontal pole, medial orbitofrontal, paracentral, precentral, and superior frontal regions (e-Table 12). The shallower age-related slope in left parietal CT in 22qDel-PS+ was observed regionally in the inferior parietal, precuneus, and superior parietal regions. The shallower age-related slope in bilateral occipital cortices was observed regionally in pericalcarine regions.

The steeper frontal CT slope in late adolescence/early adulthood in 22qDel-PS+ was observed bilaterally in the pars orbitalis, pars triangularis, and paracentral regions. The steeper parietal slope in late adolescence/adulthood in 22qDel-PS+ was observed bilaterally in the inferior parietal, postcentral, precuneus, and superior parietal regions. In contrast, there was a shallower occipital slope in late adolescence/early adulthood in 22qDel-PS+ , observed bilaterally in the cuneus, lateral occipital, and pericalcarine regions ( e-Table 12).

**References**

[1. First MB, Gibbon M. The Structured Clinical Interview for DSM-IV Axis I Disorders (SCID-I) and the Structured Clinical Interview for DSM-IV Axis II Disorders (SCID-II). In: Comprehensive handbook of psychological assessment, Vol 2: Personality assessment. Hoboken, NJ, US: John Wiley & Sons Inc; 2004. p. 134–43.](https://www.zotero.org/google-docs/?WzAo2d)

[2. Shaffer D, Fisher P, Lucas CP, Dulcan MK, Schwab-stone ME. NIMH Diagnostic Interview Schedule for Children Version IV (NIMH DISC-IV): Description, Differences From Previous Versions, and Reliability of Some Common Diagnoses. Journal of the American Academy of Child & Adolescent Psychiatry. 2000 Jan 1;39(1):28–38.](https://www.zotero.org/google-docs/?WzAo2d)

[3. Lin A, Ching CRK, Vajdi A, Sun D, Jonas RK, Jalbrzikowski M, et al. Mapping 22q11.2 Gene Dosage Effects on Brain Morphometry. J Neurosci. 2017 Jun 28;37(26):6183–99.](https://www.zotero.org/google-docs/?WzAo2d)

[4. Jalbrzikowski M, Jonas R, Senturk D, Patel A, Chow C, Green MF, et al. Structural abnormalities in cortical volume, thickness, and surface area in 22q11.2 microdeletion syndrome: Relationship with psychotic symptoms. NeuroImage: Clinical. 2013 Jan 1;3:405–15.](https://www.zotero.org/google-docs/?WzAo2d)

[5. Jalbrzikowski M, Bearden CE. Biotypes: The Tip of the Research Domain Criteria Iceberg. Biological Psychiatry: Cognitive Neuroscience and Neuroimaging. 2016 Nov 1;1(6):486–7.](https://www.zotero.org/google-docs/?WzAo2d)

[6. McGlashan TH, Miller TJ, Woods SW, Hoffman RE, Davidson L. Instrument for the Assessment of Prodromal Symptoms and States. In: Miller T, Mednick SA, McGlashan TH, Libiger J, Johannessen JO, editors. Early Intervention in Psychotic Disorders [Internet]. Dordrecht: Springer Netherlands; 2001 [cited 2021 Sep 22]. p. 135–49. (NATO Science Series). Available from: https://doi.org/10.1007/978-94-010-0892-1_7](https://www.zotero.org/google-docs/?WzAo2d)

[7. Cornblatt BA, Auther AM, Niendam T, Smith CW, Zinberg J, Bearden CE, et al. Preliminary Findings for Two New Measures of Social and Role Functioning in the Prodromal Phase of Schizophrenia. Schizophrenia Bulletin. 2007 May 1;33(3):688–702.](https://www.zotero.org/google-docs/?WzAo2d)

[8. Lord C, Risi S, Lambrecht L, Cook EH, Leventhal BL, DiLavore PC, et al. The autism diagnostic observation schedule-generic: a standard measure of social and communication deficits associated with the spectrum of autism. J Autism Dev Disord. 2000 Jun;30(3):205–23.](https://www.zotero.org/google-docs/?WzAo2d)

[9. Lord C, Rutter M, Le Couteur A. Autism Diagnostic Interview-Revised: a revised version of a diagnostic interview for caregivers of individuals with possible pervasive developmental disorders. J Autism Dev Disord. 1994 Oct;24(5):659–85.](https://www.zotero.org/google-docs/?WzAo2d)

[10. First MB, Spitzer RL, Williams J. Structured clinical Interview for DSM-IV axis I disorders clinician version SCID-I. Developmental disorder addition. American Psychiatric Association. 2009;](https://www.zotero.org/google-docs/?WzAo2d)

[11. Diagnostic and statistical manual of mental disorders: DSM-IV 4th ed. 4th ed. Washington (DC): American Psychiatric Association; 1994.](https://www.zotero.org/google-docs/?WzAo2d)

[12. Jalbrzikowski M, Ahmed KH, Patel A, Jonas R, Kushan L, Chow C, et al. Categorical versus dimensional approaches to autism-associated intermediate phenotypes in 22q11.2 microdeletion syndrome. Biol Psychiatry Cogn Neurosci Neuroimaging. 2017 Jan;2(1):53–65.](https://www.zotero.org/google-docs/?WzAo2d)

[13. Wechsler D. Wechsler Abbreviated Scale of Intelligence-Second Edition (WASI-II). Pearson; 2011. book.](https://www.zotero.org/google-docs/?WzAo2d)

[14. Kreutzer JS, DeLuca J, Caplan B, editors. WAIS-IV. In: Encyclopedia of Clinical Neuropsychology [Internet]. New York, NY: Springer New York; 2011. p. 2667–2667. Available from: https://doi.org/10.1007/978-0-387-79948-3_4124](https://www.zotero.org/google-docs/?WzAo2d)

[15. FreeSurferMethodsCitation - Free Surfer Wiki [Internet]. [cited 2021 Feb 10]. Available from: https://surfer.nmr.mgh.harvard.edu/fswiki/FreeSurferMethodsCitation](https://www.zotero.org/google-docs/?WzAo2d)

[16. Dale AM, Fischl B, Sereno MI. Cortical Surface-Based Analysis: I. Segmentation and Surface Reconstruction. NeuroImage. 1999 Feb 1;9(2):179–94.](https://www.zotero.org/google-docs/?WzAo2d)

[17. Fischl B, Sereno MI, Dale AM. Cortical surface-based analysis. II: Inflation, flattening, and a surface-based coordinate system. Neuroimage. 1999 Feb;9(2):195–207.](https://www.zotero.org/google-docs/?WzAo2d)

[18. Desikan RS, Ségonne F, Fischl B, Quinn BT, Dickerson BC, Blacker D, et al. An automated labeling system for subdividing the human cerebral cortex on MRI scans into gyral based regions of interest. NeuroImage. 2006 Jul 1;31(3):968–80.](https://www.zotero.org/google-docs/?WzAo2d)

[19. Thompson PM, Stein JL, Medland SE, Hibar DP, Vasquez AA, Renteria ME, et al. The ENIGMA Consortium: large-scale collaborative analyses of neuroimaging and genetic data. Brain Imaging and Behavior. 2014 Jun;8(2):153–82.](https://www.zotero.org/google-docs/?WzAo2d)

[20. van Erp TGM, Hibar DP, Rasmussen JM, Glahn DC, Pearlson GD, Andreassen OA, et al. Subcortical brain volume abnormalities in 2028 individuals with schizophrenia and 2540 healthy controls via the ENIGMA consortium. Molecular Psychiatry. 2016 Apr;21(4):547–53.](https://www.zotero.org/google-docs/?WzAo2d)

[21. Sun Z, Williams DJ, Xu B, Gogos JA. Altered function and maturation of primary cortical neurons from a 22q11.2 deletion mouse model of schizophrenia. Translational Psychiatry. 2018 Apr 18;8(1):1–14.](https://www.zotero.org/google-docs/?WzAo2d)

[22. Thompson PM, Andreassen OA, Arias-Vasquez A, Bearden CE, Boedhoe PS, Brouwer RM, et al. ENIGMA and the individual: Predicting factors that affect the brain in 35 countries worldwide. NeuroImage. 2017 Jan 15;145:389–408.](https://www.zotero.org/google-docs/?WzAo2d)

[23. Imaging Protocols « ENIGMA [Internet]. [cited 2021 Feb 10]. Available from: http://enigma.ini.usc.edu/protocols/imaging-protocols/](https://www.zotero.org/google-docs/?WzAo2d)

[24. Reuter M, Schmansky NJ, Rosas HD, Fischl B. Within-subject template estimation for unbiased longitudinal image analysis. Neuroimage. 2012 Jul 16;61(4):1402–18.](https://www.zotero.org/google-docs/?WzAo2d)

[25. Reuter M, Rosas HD, Fischl B. Highly accurate inverse consistent registration: A robust approach. NeuroImage. 2010 Dec 1;53(4):1181–96.](https://www.zotero.org/google-docs/?WzAo2d)

[26. Beer JC, Tustison NJ, Cook PA, Davatzikos C, Sheline YI, Shinohara RT, et al. Longitudinal ComBat: A method for harmonizing longitudinal multi-scanner imaging data. NeuroImage. 2020 Oct 15;220:117129.](https://www.zotero.org/google-docs/?WzAo2d)

[27. Fortin J-P, Cullen N, Sheline YI, Taylor WD, Aselcioglu I, Cook PA, et al. Harmonization of cortical thickness measurements across scanners and sites. Neuroimage. 2018 Feb 15;167:104–20.](https://www.zotero.org/google-docs/?WzAo2d)

[28. Calabro FJ, Murty VP, Jalbrzikowski M, Tervo-Clemmens B, Luna B. Development of Hippocampal–Prefrontal Cortex Interactions through Adolescence. Cereb Cortex. 2020 Mar;30(3):1548–58.](https://www.zotero.org/google-docs/?WzAo2d)

[29. Bridgwater M, Bachman P, Tervo-Clemmens B, Haas G, Hayes R, Luna B, et al. Developmental Influences on Symptom Expression in Antipsychotic-Naïve First-Episode Psychosis [Internet]. 2020 Jun [cited 2021 Sep 12] p. 2020.06.19.160093. Available from: https://www.biorxiv.org/content/10.1101/2020.06.19.160093v1](https://www.zotero.org/google-docs/?WzAo2d)

[30. Lin A, Vajdi A, Kushan-Wells L, Helleman G, Hansen LP, Jonas RK, et al. Reciprocal Copy Number Variations at 22q11.2 Produce Distinct and Convergent Neurobehavioral Impairments Relevant for Schizophrenia and Autism Spectrum Disorder. Biological Psychiatry. 2020 Aug 1;88(3):260–72.](https://www.zotero.org/google-docs/?WzAo2d)

[31. Wood SN. Generalized Additive Models: An Introduction with R, Second Edition. CRC Press; 2017. 497 p.](https://www.zotero.org/google-docs/?WzAo2d)
