# Supplementary material for: Longitudinal trajectories of cortical development in 22q11.2 copy number variants and typically developing controls
Source: Mol Psychiatry. 2022 Jul 27;27(10):4181–90. doi: 10.1038/s41380-022-01681-w (PMC9718681; doi:10.1038/s41380-022-01681-w)
Supplement: Supplementary file 2 — Supplemental Tables [file 41380_2022_1681_MOESM2_ESM.docx]

SUPPLEMENTAL TABLES : Contents

[e-Table 1: Comparison of demographics between study participants with single vs. multiple timepoints 3](#_Toc104933663)

[e-Table 2. Baseline psychotropic medications in 22q11.2 CNV carriers 5](#_Toc104933664)

[e-Table 3. Regions of interest included in lobar summary measures 6](#_Toc104933665)

[e-Table 4. Demographics for 22qDel-Psychosis Spectrum (PS+) vs. 7](#_Toc104933666)

[22qDel-No Psychosis (PS-) 7](#_Toc104933667)

[e-Table 5. Demographics for 22qDel-ASD vs. 22qDel-no ASD ^a^ 8](#_Toc104933668)

[e-Table 6. Baseline Demographics for 22qDup-ASD vs. 22qDup-no ASD 9](#_Toc104933669)

[e-Table 7a. Group and Age Effects on Neurodevelopmental Trajectories in 22q11.2 CNV Carriers vs. Controls: Overall and Lobar Results 10](#_Toc104933670)

[e-Table 7b. Group and Age Effects on Neurodevelopmental Trajectories in 22q11.2 CNV Carriers vs. Controls: CT Overall and Lobar Results (without ICV adjustment) 11](#_Toc104933671)

[e-Table 8. Group and Age Effects on CT and SA Neurodevelopmental Trajectories in 22q11.2 CNV Carriers vs. Controls: Individual ROI Results 12](#_Toc104933672)

[e-Table 9. Group and Age Effects on Neurodevelopmental Trajectories in 22q11.2 CNV Carriers vs. Controls, covarying for IQ 16](#_Toc104933673)

[e-Table 10. Group and Age Effects on Neurodevelopmental Trajectories in 22q11.2 CNV Carriers vs. Controls, covarying for antipsychotic medication use 17](#_Toc104933674)

[e-Table 11a. Neurodevelopmental CT and SA trajectories in 22qDel-PS+ vs. 22qDel-PS- 19](#_Toc104933675)

[e-Table 11b. Neurodevelopmental CT trajectories in 22qDel-PS+ vs. 22qDel-PS- (without ICV adjustment) 21](#_Toc104933676)

[e-Table 12. Individual ROI Results for 22qDel-PS+ vs. 22qDel-PS– 22](#_Toc104933677)

[e-Table 13. Neurodevelopmental CT and SA trajectories in 22qDel-PS+ vs. 22qDel-PS-, covarying for antipsychotic medication use. 25](#_Toc104933678)

[e-Table 14. Neurodevelopmental CT and SA trajectories in 22qDel-PS+ vs. 22qDel-PS-, under 35 years old. 26](#_Toc104933679)

[e-Table 15. Neurodevelopmental CT and SA trajectories in 22qDel-PS+ vs. 22qDel-PS-, covarying for comorbid ASD diagnosis. 27](#_Toc104933680)

[e-Table 16. Neurodevelopmental CT and SA trajectories in 22qDel-PS+ vs. 22qDel-PS- when Psychosis Spectrum status varies as a function of visit 28](#_Toc104933681)

[e-Table 17a. Neurodevelopmental CT and SA trajectories in 22qDel-ASD vs. 22qDel-No ASD 29](#_Toc104933682)

[e-Table 17b. Neurodevelopmental CT trajectories in 22qDel-ASD vs. 22qDel-No ASD (without ICV adjustment) 30](#_Toc104933683)

[e-Table 18. Individual ROI Table for 22qDel-ASD vs. 22qDel-no ASD 31](#_Toc104933684)

[e- Table 19. 22q Del-ASD vs 22qDel-no ASD, covarying for comorbid Psychosis Spectrum Symptoms 33](#_Toc104933685)

[e-Table 20a. Neurodevelopmental CT and SA trajectories in 22qDup-ASD vs. 22qDup-no ASD 34](#_Toc104933686)

[e-Table 20b. Neurodevelopmental CT trajectories in 22qDup-ASD vs. 22qDup-no ASD (without ICV adjustment) 35](#_Toc104933687)

[e-Table 21. Individual ROI Table for 22qDup-ASD vs. 22qDup-no ASD 36](#_Toc104933688)

# e-Table 1: Comparison of demographics between study participants with single vs. multiple timepoints

|  | **One scan only** | | | **More than one scan** | | |
| --- | --- | --- | --- | --- | --- | --- |
|  | 22qDel | TD Controls^a^ | 22qDup | 22qDel | TD Controls | 22qDup |
| **Baseline n** | 38 | 51 | 20 | 46 | 43 | 14 |
| **Age (SD)** | 16.31 (8.54) | 14.54 (7.00) | 19.69 (15.25) | 18.40 (8.06) | 12.89 (5.27) | 17.11 (11.55) |
| **Age**  **Range** | 5.5-39.4 | 6-45 | 6.7-49.5 | 6.1-42.7 | 6.1-29.1 | 8.2-42.8 |
| **N, females (%)** | 15 (39.47%) | 26 (50.98%) | 10 (50%) | 26 (56.52%) | 24 (55.81%) | 4 (28.57%) |
| **N, right-handed (%)** | 31 (81.58%) | 45 (88%) | 17 (85%) | 40 (86.96%) | 37 (86.05%) | 11 (78.57%) |
| **N, Hispanic (%)** | 4 (10.53%)**^b^** | 21 (41.18%) | 0 (0%) | 13 (28.26%)^b^ | 16 (37.21%) | 0 (0%) |
| **Race^c^** |  | | | | | |
| **American Indian/Alaska Native (%)** | 0% | 0% | 0% | 0% | 0% | 0% |
| **Asian (%)** | 0% | 5.88% | 0% | 2.17% | 4.65% | 2.56% |
| **Black (%)** | 0% | 11.76% | 0% | 2.17% | 11.63% | 0% |
| **White (%)** | 92.11% | 66.67% | 100% | 86.96% | 74.42% | 92.86% |
| **Multiple (%)** | 7.89% | 15.69% | 0% | 8.70% | 9.30% | 0% |
| **N, Psychotic Disorder (%)** | 3 (7.89%) | 0 | 0 | 5 (10.87%) | 0 | 0 |
| **N, Psychosis-Risk (%)** | 7 (18.42%) | 0 | 0 | 10 (44.44%) | 0 | 0 |
| **Parental education, years (SD)** | 15.41 (2.69) | 13.32 (3.67)**^d^** | 14.2 (2.38) | 14.81 (3.18) | 16.06 (2.95)**^d^** | 13.79 (2.67) |
| **Full Scale IQ (SD)** | 80.11 (13.17) | 111.18 (16.73) | 95.05 (19.23) | 79.93 (12.65) | 112.95 (19.27) | 96.64 (17.87) |

Comparisons were made within groups (e.g., between 22qDel with 1 scan vs. 22qDel with multiple scans). Two sample t-tests or their non-parametric equivalent were used. For distribution comparisons, chi-squared tests were used. Unless otherwise noted, there were no significant differences between groups.

**^a^** CNP controls (cross-sectional study) are excluded for this comparison.

**^b^** 22qDel, 1 timepoint ≠ 22qDel, more than 1 timepoint (p<.05)

**^c^** Participants did not differ in overall race distribution between groups.

**^d^** Controls, 1 timepoint ≠ controls, more than 1 timepoint (p<.05)

# e-Table 2. Baseline psychotropic medications in 22q11.2 CNV carriers

|  | 22q11.2  Deletion Carriers | 22q11.2  Duplication Carriers |
| --- | --- | --- |
| **N** | 84 | 34 |
| **Class of medication** |  |  |
| **N, Typical Antipsychotic (%)** | 0 | 0 |
| **N, Atypical Antipsychotic (%)** | 1 (1.19%) | 0 |
| **N, Mood Stabilizer / Anticonvulsant (%)** | 2 (2.38%) | 0 |
| **N, Antidepressant (%)** | 1 (1.19%) | 0 |
| **N, Tricyclic (%)** | 0 | 0 |
| **N, SSRI and SNRI (%)** | 6 (7.14%) | 1 (2.94%) |
| **N, Psychostimulant (%)** | 3 (3.57%) | 4 (11.76%) |
| **N, Benzodiazepine** | 0 | 0 |
| **N, Non-stimulant ADHD med (%)** | 0 | 0 |
| **N, More than one class of medication^a^ (%)** | 14 (16.67%) | 8 (23.53%) |

^a^ Includes 9 22qDel and 2 22qDup on atypical antipsychotics + another medication

# e-Table 3. Regions of interest included in lobar summary measures

| **Frontal Lobe** |  | **Parietal Lobe** |  | **Temporal Lobe** |  | **Occipital Lobe** |
| --- | --- | --- | --- | --- | --- | --- |
| Caudal anterior cingulate |  | Inferior parietal |  | Bank of the superior temporal sulcus |  | Cuneus |
| Caudal middle frontal gyrus |  | Isthmus cingulate |  | Entorhinal cortex |  | Lateral occipital gyrus |
| Frontal pole |  | Postcentral gyrus |  | Fusiform gyrus |  | Lingual gyrus |
| Medial orbitofrontal |  | Posterior cingulate |  | Inferior temporal gyrus |  | Pericalcarine region |
| Lateral orbitofrontal |  | Precuneus |  | Insula |  |  |
| Paracentral gyrus |  | Superior parietal |  | Middle temporal gyrus |  |  |
| Pars opercularis |  | Supramarginal gyrus |  | Parahippocampal gyrus |  |  |
| Pars orbitalis |  |  |  | Superior temporal gyrus |  |  |
| Pars triangularis |  |  |  | Temporal pole |  |  |
| Precentral gyrus |  |  |  | Transverse temporal gyrus |  |  |
| Rostral anterior cingulate |  |  |  |  |  |  |
| Rostral middle frontal gyrus |  |  |  |  |  |  |
| Superior frontal gyrus |  |  |  |  |  |  |

# e-Table 4. Demographics for 22qDel-Psychosis Spectrum (PS+) vs.

# 22qDel-No Psychosis (PS-)*

|  | 22q11DS-PS+ | 22q11DS-PS- |
| --- | --- | --- |
| **N** | 30 | 37 |
| **Age (SD)** | 18.6 (6.5) | 19. 2 (8.6) |
| **Age Range** | 10.0-42.0 | 6.6-42.7 |
| **N, females (%)** | 18 (60%) | 20 (54.0%) |
| **Race** |  |  |
| **N, American Indian/Alaska Native (%)** | 0 | 0 |
| **N, Asian (%)** | 0 | 1 (2.7%) |
| **Black or African American (%)** | 0 | 1 (2.7%) |
| **N, White (%)** | 27 (90%) | 33 (89.2%) |
| **N, Multiple Race (%)** | 3 (10%) | 2 (5.4%) |
| **N, Right-Handed (%)** | 29 | 29 |
| **Full Scale IQ (SD)** | 79.5 (13.8) | 80.2 (12.3) |
| **N, Autism Spectrum Disorder (ASD), (%)** | 19 (63%) | 16 (43%) |
| **Scanner** |  |  |
| **N, BMC** | 10 | 5 |
| **N, CCN** | 8 | 18 |
| **N, PRISMA** | 12 | 14 |

22qDel with and without Psychosis Spectrum symptoms did not significantly differ on age (*p*=0.73), sex (*p*=0.81), race (*p*=0.55), handedness (*p*=0.08 ), IQ (*p*=0.82), rate of ASD diagnosis (*p*=0.16), or scanner (*p*=0.08).

# e-Table 5. Demographics for 22qDel-ASD vs. 22qDel-no ASD ^a^

|  | 22q11Del-ASD | 22q11Del-no ASD |
| --- | --- | --- |
| N | 39 | 31 |
| Age (SD) | 15.2 (5.6) | 15.2 (6.0) |
| Age Range | 6.1-25.2 | 5.5-24.7 |
| N, females (%) | 18 (46.0%) | 17 (54.8%) |
| Race |  |  |
| N, American Indian/Alaska Native (%) | 0 (0%) | 0 (0%) |
| N, Asian (%) | 0 (0%) | 1 (3.2%) |
| Black or African American (%) | 0 (0%) | 1 (3.2%) |
| N, White (%) | 35 (89.7%) | 26 (83.9%) |
| N, Multiple (%) | 4 (10.3%) | 3 (9.7%) |
| N, Right-Handed (%) | 35 (89.7%) | 27 (87.1%) |
| Full Scale IQ (SD) | 77.9 (9.7) | 80.5 (14.0) |
| N, Psychotic Disorder, (%) | 4 (10.2%) | 1(3.5%) |
| N, Psychosis-Risk, (%) | 14 (36%) | 4 (14%) |
| Scanner |  |  |
| N, BMC | 11 | 10 |
| N, CCN | 15 | 11 |
| N, PRISMA | 13 | 10 |

^a^ Analysis restricted to subjects < 25 years old who were assessed with ADI/ADOS.

^b^Groups did not significantly differ on psychosis risk or psychotic disorder category (*p*=0.16), age (*p*=0.95), sex (*p*=0.63), race (*p*=0.46), handedness (*p*=0.51), IQ (*p*=0.40), or scanner (*p*=0.93).

# e-Table 6. Baseline Demographics for 22qDup-ASD vs. 22qDup-no ASD

|  | 22q11Dup-ASD | 22q11Dup-no ASD |
| --- | --- | --- |
| N | 14 | 12 |
| Age (SD) | 12.0 (4.8) | 11.0 (3.2) |
| Age Range | 6.7-24.8 | 8.3-19.7 |
| N, females (%) | 5 (35.7%) | 5 (41.7%) |
| Race |  |  |
| N, American Indian/Alaska Native (%) | 0 (0%) | 0 (0%) |
| N, Asian (%) | 0 (0%) | 1 (8.3%) |
| Black or African American (%) | 0 (0%) | 0 (0%) |
| N, White (%) | 14 (100%) | 11 (91.7%) |
| N, Multiple (%) | 0 (0%) | 0 (0%) |
| N, Right-Handed (%) | 11 (78%) | 9 (75%) |
| Full Scale IQ (SD) | 93.2 (19.2) | 89.2 (18.3) |
| N, Psychosis Risk, (%)^a^ | 4 (28.6%) | 0 (0.0%) |
| Scanner |  |  |
| N, BMC | 0 | 0 |
| N, CCN | 10 | 7 |
| N, PRISMA | 4 | 5 |

^a^  Excludes one subject over age 25 who was not included in ASD analyses.

22qDup-ASD and 22qDup-no ASD did not significantly differ with regard to age (*p*=0.56), sex (*p*=0.99), race (*p*=0.94), handedness (*p*=0.99), IQ (*p*=0.60), psychosis risk criteria (p=0.06), or scanner (p=0.77).

# e-Table 7a. Group and Age Effects on Neurodevelopmental Trajectories in 22q11.2 CNV Carriers vs. Controls: Overall and Lobar Results

|  |  | Parametric Effects of Group (Control as Reference) | | | | | | Non-parametric (smooth) Effects of Age | | | | | | | | | Age range(s) when significant change is taking place | | | Age range(s) when smoothed effects of age differed between groups | | |
| --- | --- | --- | --- | --- | --- | --- | --- | --- | --- | --- | --- | --- | --- | --- | --- | --- | --- | --- | --- | --- | --- | --- |
|  |  | **22qdel** | | | **22qdup** | | | **Controls** | | | **22qdel** | | | **22qdup** | | | **Controls** | **22qdel** | **22qdup** | **Controls vs 22qdel** | **Controls vs 22qdup** | **22qdel vs 22qdup** |
| **brain** | **hemisphere** | **T** | **p** | **q** | **T** | **p** | **q** | **F** | **p** | **q** | **F** | **p** | **q** | **F** | **p** | **q** |  |  |  |  |  |  |
| Overall Mean Thickness |  | 6.99 | 0.00 | 0.00 | -3.97 | 0.00 | 0.00 | 100.08 | 0.00 | 0.00 | 60.13 | 0.00 | 0.00 | 31.42 | 0.00 | 0.00 | 6-24.1 | 5.5-33.6 | 6-21.9 | 13.9-21.5 ; 36.2-38.8 | NA | NA |
| Frontal Thickness | L | 6.29 | 0.00 | 0.00 | -2.35 | 0.02 | 0.03 | 107.89 | 0.00 | 0.00 | 60.33 | 0.00 | 0.00 | 22.20 | 0.00 | 0.00 | 6-25 | 5.5-29.6 | 6-22.5 | 18.4-19.3 | NA | NA |
| Frontal Thickness | R | 5.05 | 0.00 | 0.00 | -3.61 | 0.00 | 0.00 | 81.60 | 0.00 | 0.00 | 55.95 | 0.00 | 0.00 | 16.84 | 0.00 | 0.00 | 6-26.7 | 5.5-31.6 | 6-22.7 | 14.8-17.9 | NA | NA |
| Temporal Thickness | L | -0.90 | 0.37 | 0.38 | -1.89 | 0.06 | 0.07 | 28.66 | 0.00 | 0.00 | 39.00 | 0.00 | 0.00 | 13.96 | 0.00 | 0.00 | 6-31.1 | 5.5-49.5 | 6-35.6 | 15.7-21.1 | NA | NA |
| Temporal Thickness | R | 1.97 | 0.05 | 0.06 | -3.92 | 0.00 | 0.00 | 28.14 | 0.00 | 0.00 | 47.40 | 0.00 | 0.00 | 12.21 | 0.00 | 0.00 | 6-26.9 | 5.5-49.5 | 6-31.1 | 13.9-21.5 ; 36.2-49.5 | NA | NA |
| Parietal Thickness | L | 3.51 | 0.00 | 0.00 | -2.70 | 0.01 | 0.01 | 90.15 | 0.00 | 0.00 | 62.74 | 0.00 | 0.00 | 16.60 | 0.00 | 0.00 | 6-24.7 | 5.5-40.4 | 6-22.3 | 13.1-22.8 ; 33.9-49.5 | NA | 37.1-41.9 |
| Parietal Thickness | R | 3.64 | 0.00 | 0.00 | -3.88 | 0.00 | 0.00 | 88.78 | 0.00 | 0.00 | 55.13 | 0.00 | 0.00 | 22.48 | 0.00 | 0.00 | 6-23 | 5.5-35.1 | 6-20.3 | 6-7.7 ; 13.1-21.9 ; 32.2-41.1 | NA | 12.6-15.7 ; 31.3-40.6 |
| Occipital Thickness | L | 6.80 | 0.00 | 0.00 | -4.84 | 0.00 | 0.00 | 47.10 | 0.00 | 0.00 | 18.29 | 0.00 | 0.00 | 5.52 | 0.00 | 0.00 | 6-21.2 | 5.5-20.3 | 6-19.9 | 19.7-21.9 | NA | NA |
| Occipital Thickness | R | 8.63 | 0.00 | 0.00 | -5.79 | 0.00 | 0.00 | 49.50 | 0.00 | 0.00 | 22.55 | 0.00 | 0.00 | 13.38 | 0.00 | 0.00 | 6-21.4 | 5.5-21.2 | 6-20.5 | NA | NA | NA |
|  |  |  |  |  |  |  |  |  |  |  |  |  |  |  |  |  |  |  |  |  |  |  |
| Total Surface Area |  | -32.39 | 0.00 | 0.00 | 5.94 | 0.00 | 0.00 | 7.91 | 0.00 | 0.00 | 17.11 | 0.00 | 0.00 | 2.05 | 0.15 | 0.17 | 6-23 | 5.5-49.5 | NA | NA | NA | NA |
| Frontal Area | L | -14.61 | 0.00 | 0.00 | 4.33 | 0.00 | 0.00 | 3.09 | 0.03 | 0.04 | 12.92 | 0.00 | 0.00 | 0.83 | 0.36 | 0.38 | 6-22.7 | 5.5-49.5 | NA | NA | NA | NA |
| Frontal Area | R | -21.88 | 0.00 | 0.00 | 8.06 | 0.00 | 0.00 | 2.41 | 0.12 | 0.14 | 6.50 | 0.00 | 0.00 | 1.50 | 0.22 | 0.24 | NA | 14.8-32 | NA | 10.4-16.2 ; 23.7-39.3 | NA | 12.2-15.7 ; 24.6-36.2 |
| Temporal Area | L | -18.90 | 0.00 | 0.00 | 2.87 | 0.00 | 0.01 | 4.00 | 0.05 | 0.06 | 4.05 | 0.05 | 0.06 | 1.73 | 0.19 | 0.21 | NA | NA | NA | NA | NA | NA |
| Temporal Area | R | -34.16 | 0.00 | 0.00 | 5.29 | 0.00 | 0.00 | 2.84 | 0.09 | 0.11 | 7.23 | 0.01 | 0.01 | 3.27 | 0.07 | 0.09 | NA | 5.5-49.5 | NA | NA | NA | NA |
| Parietal Area | L | -23.68 | 0.00 | 0.00 | 1.60 | 0.11 | 0.13 | 15.08 | 0.00 | 0.00 | 4.90 | 0.00 | 0.01 | 1.66 | 0.14 | 0.16 | 6-21.2 | 5.5-25.6 | NA | NA | 6-8.2 ; 16.6-22.8 | NA |
| Parietal Area | R | -41.27 | 0.00 | 0.00 | 6.43 | 0.00 | 0.00 | 18.27 | 0.00 | 0.00 | 6.87 | 0.00 | 0.00 | 2.45 | 0.06 | 0.07 | 6-23.4 | 5.5-30.9 | NA | 18.4-20.6 | NA | NA |
| Occipital Area | L | -28.95 | 0.00 | 0.00 | 4.52 | 0.00 | 0.00 | 2.63 | 0.05 | 0.06 | 2.34 | 0.13 | 0.14 | 0.00 | 0.98 | 0.98 | NA | NA | NA | NA | NA | NA |
| Occipital Area | R | -46.87 | 0.00 | 0.00 | 2.67 | 0.01 | 0.01 | 0.00 | 0.97 | 0.98 | 1.43 | 0.31 | 0.33 | 1.49 | 0.22 | 0.24 | NA | NA | NA | NA | NA | NA |

# e-Table 7b. Group and Age Effects on Neurodevelopmental Trajectories in 22q11.2 CNV Carriers vs. Controls: CT Overall and Lobar Results (without ICV adjustment)

|  |  | Parametric Effects of Group (Control as Reference) | | | | | | Non-parametric (smooth) Effects of Age | | | | | | | | | Age range(s) when significant change is taking place | | | Age range(s) when smoothed effects of age differed between groups | | |
| --- | --- | --- | --- | --- | --- | --- | --- | --- | --- | --- | --- | --- | --- | --- | --- | --- | --- | --- | --- | --- | --- | --- |
|  |  | 22qdel | | | 22qdup | | | Control | | | 22qdel | | | 22qdup | | | Controls | 22qDel | 22qdup | Controls vs 22qdel | Controls vs 22qdup | 22qdel vs 22qdup |
| **brain** | **hemisphere** | **T** | **p** | **q** | **T** | **p** | **q** | **F** | **p** | **q** | **F** | **p** | **q** | **F** | **p** | **q** |  |  |  |  |  |  |
| Mean Thickness |  | 7.39 | 0.00 | 0.00 | -3.98 | 0.00 | 0.00 | 97.34 | 0.00 | 0.00 | 64.35 | 0.00 | 0.00 | 33.14 | 0.00 | 0.00 | 5.5-24.3 | 5.5-33.4 | 5.5-21.9 | 14.4-20.6 | NA | NA |
| Frontal Thickness | L | 6.99 | 0.00 | 0.00 | -2.26 | 0.02 | 0.03 | 107.72 | 0.00 | 0.00 | 66.59 | 0.00 | 0.00 | 23.24 | 0.00 | 0.00 | 5.5-25 | 5.5-29.2 | 5.5-23.4 | NA | NA | NA |
| Frontal Thickness | R | 6.29 | 0.00 | 0.00 | -3.45 | 0.00 | 0.00 | 88.40 | 0.00 | 0.00 | 64.30 | 0.00 | 0.00 | 18.89 | 0.00 | 0.00 | 5.5-26.3 | 5.5-31.4 | 5.5-23.4 | 14.8-16.6 | NA | NA |
| Temporal Thickness | L | -0.58 | 0.57 | 0.57 | -1.72 | 0.09 | 0.09 | 29.50 | 0.00 | 0.00 | 43.22 | 0.00 | 0.00 | 18.79 | 0.00 | 0.00 | 5.5-30.9 | 5.5-49.5 | 5.5-49.5 | 15.3-21.5 ; 40.6-49.5 | NA | NA |
| Temporal Thickness | R | 2.11 | 0.04 | 0.04 | -3.91 | 0.00 | 0.00 | 29.47 | 0.00 | 0.00 | 51.40 | 0.00 | 0.00 | 14.36 | 0.00 | 0.00 | 5.5-27.2 | 5.5-49.5 | 5.5-22.1 | 13.9-21.5 ; 35.7-49.5 | NA | NA |
| Parietal Thickness | L | 4.02 | 0.00 | 0.00 | -2.67 | 0.01 | 0.01 | 88.97 | 0.00 | 0.00 | 64.72 | 0.00 | 0.00 | 17.84 | 0.00 | 0.00 | 5.5-24.7 | 5.5-39.8 | 5.5-22.5 | 13.5-22.4 ; 33.9-49.5 | NA | 37.5-41.1 |
| Parietal Thickness | R | 4.20 | 0.00 | 0.00 | -3.80 | 0.00 | 0.00 | 89.25 | 0.00 | 0.00 | 60.99 | 0.00 | 0.00 | 25.13 | 0.00 | 0.00 | 5.5-23 | 5.5-34.7 | 5.5-20.3 | 5.5-7.3 ; 13.1-21.9 ; 32.2-41.5 | NA | 12.6-15.3 ; 31.3-40.6 |
| Occipital Thickness | L | 6.82 | 0.00 | 0.00 | -4.81 | 0.00 | 0.00 | 44.09 | 0.00 | 0.00 | 19.49 | 0.00 | 0.00 | 11.57 | 0.00 | 0.00 | 5.5-21.2 | 5.5-20.1 | 5.5-19.9 | NA | NA | NA |
| Occipital Thickness | R | 8.37 | 0.00 | 0.00 | -5.80 | 0.00 | 0.00 | 45.13 | 0.00 | 0.00 | 22.40 | 0.00 | 0.00 | 16.50 | 0.00 | 0.00 | 5.5-21.4 | 5.5-21 | 5.5-20.3 ; 29.2-34 | NA | NA | 18.4-21.5 |

# e-Table 8. Group and Age Effects on CT and SA Neurodevelopmental Trajectories in 22q11.2 CNV Carriers vs. Controls: Individual ROI Results

|  |  | Parametric Effects of Group (Control as Reference) | | | | | | Non-parametric (smooth) Effects of Age | | | | | | | | | Age range(s) when significant change is taking place | | | Age range(s) when smoothed effects of age differed between groups | | |
| --- | --- | --- | --- | --- | --- | --- | --- | --- | --- | --- | --- | --- | --- | --- | --- | --- | --- | --- | --- | --- | --- | --- |
|  |  | **22qdel** | | | **22qdup** | | | **Controls** | | | **22qdel** | | | **22qdup** | | | **Controls** | **22qdel** | **22qdup** | **Controls vs 22qdel** | **Controls vs 22qdup** | **22qdel vs 22qdup** |
| **Brain Region** | **Hemisphere** | **T** | **p** | **q** | **T** | **p** | **q** | **F** | **p** | **q** | **F** | **p** | **q** | **F** | **p** | **q** |  |  |  |  |  |  |
| Bank STS Thickness | L | 0.69 | 0.49 | 0.55 | 3.77 | 0.00 | 0.00 | 43.71 | 0.00 | 0.00 | 21.07 | 0.00 | 0.00 | 33.33 | 0.00 | 0.00 | 6-31.6 | 5.5-22.5 | 6-49.5 | NA | 6-9.5; 15.3-24.6 | 6-9.1; 14.8-23.7 |
| Bank STS Thickness | R | 4.45 | 0.00 | 0.00 | 0.78 | 0.43 | 0.48 | 51.02 | 0.00 | 0.00 | 20.83 | 0.00 | 0.00 | 19.49 | 0.00 | 0.00 | 6-23.2 | 5.5-28.5 | 6-25.8; 28.7-34.7 | 6-8.6 | NA | NA |
| Entorhinal Thickness | L | -1.43 | 0.16 | 0.21 | -5.67 | 0.00 | 0.00 | 0.55 | 0.55 | 0.60 | 0.32 | 0.57 | 0.62 | 0.57 | 0.45 | 0.50 | NA | NA | NA | NA | NA | NA |
| Entorhinal Thickness | R | 1.33 | 0.18 | 0.24 | -4.59 | 0.00 | 0.00 | 0.53 | 0.64 | 0.69 | 1.42 | 0.24 | 0.29 | 0.03 | 0.87 | 0.88 | NA | NA | NA | NA | NA | NA |
| Fusiform Thickness | L | 0.16 | 0.88 | 0.89 | -2.92 | 0.00 | 0.01 | 27.76 | 0.00 | 0.00 | 42.46 | 0.00 | 0.00 | 30.79 | 0.00 | 0.00 | 6-23.4 | 5.5-49.5 | 6-49.5 | 13.9-23.3; 36.2-49.5 | 6-8.2; 14.4-24.2; 42.8-44.6 | NA |
| Fusiform Thickness | R | 1.70 | 0.09 | 0.13 | -2.05 | 0.04 | 0.06 | 32.22 | 0.00 | 0.00 | 42.84 | 0.00 | 0.00 | 15.09 | 0.00 | 0.00 | 6-23.4 | 5.5-49.5 | 6-35.8 | 6-8.6; 13.9-25.1; 35.7-49.5 | 17.5-18.4; 40.6-48.6 | NA |
| Inferior Temporal Thickness | L | 2.67 | 0.01 | 0.01 | 1.69 | 0.09 | 0.13 | 25.80 | 0.00 | 0.00 | 30.48 | 0.00 | 0.00 | 27.10 | 0.00 | 0.00 | 6-26.9 | 5.5-49.5 | 6-49.5 | 15.3-21.1 | 15.7-23.3 | NA |
| Inferior Temporal Thickness | R | 1.71 | 0.09 | 0.12 | -1.34 | 0.18 | 0.23 | 30.59 | 0.00 | 0.00 | 70.22 | 0.00 | 0.00 | 27.77 | 0.00 | 0.00 | 6-28.1 | 5.5-49.5 | 6-49.5 | 13.5-22.4; 34.4-49.5 | 6-9.1; 16.2-27.3; 44.6-49.5 | 6-17.5; 18.4-49.5 |
| Insula Thickness | L | 9.39 | 0.00 | 0.00 | -5.84 | 0.00 | 0.00 | 38.92 | 0.00 | 0.00 | 28.54 | 0.00 | 0.00 | 15.00 | 0.00 | 0.00 | 6-28.1 | 5.5-34.5 | 6-24.5 | NA | NA | 19.7-22.8 |
| Insula Thickness | R | 7.50 | 0.00 | 0.00 | -5.31 | 0.00 | 0.00 | 54.33 | 0.00 | 0.00 | 59.52 | 0.00 | 0.00 | 20.71 | 0.00 | 0.00 | 6-23.6 | 5.5-49.5 | 6-30.7 | 6-10.4; 13.5-25.5; 35.7-49.5 | NA | NA |
| Middle Temporal Thickness | L | 3.26 | 0.00 | 0.00 | 1.38 | 0.17 | 0.22 | 38.48 | 0.00 | 0.00 | 44.93 | 0.00 | 0.00 | 38.98 | 0.00 | 0.00 | 6-27.4 | 5.5-49.5 | 6-49.5 | 6-7.3; 14.8-24.6; 40.6-49.5 | 6-8.6; 14.8-25.1; 43.7-49.5 | NA |
| Middle Temporal Thickness | R | 5.66 | 0.00 | 0.00 | -1.28 | 0.20 | 0.26 | 34.38 | 0.00 | 0.00 | 33.01 | 0.00 | 0.00 | 15.00 | 0.00 | 0.00 | 6-28.1 | 5.5-49.5 | 6-35.3 | 14.4-19.7; 36.6-49.5 | NA | NA |
| Parahippocampal Thickness | L | -14.10 | 0.00 | 0.00 | -0.20 | 0.84 | 0.86 | 7.92 | 0.01 | 0.01 | 3.95 | 0.05 | 0.07 | 7.11 | 0.01 | 0.01 | 6-49.5 | NA | 6-49.5 | NA | NA | NA |
| Parahippocampal Thickness | R | -6.25 | 0.00 | 0.00 | -4.10 | 0.00 | 0.00 | 1.99 | 0.23 | 0.29 | 3.58 | 0.06 | 0.09 | 0.79 | 0.30 | 0.36 | NA | NA | NA | NA | NA | NA |
| Superior Temporal Thickness | L | -5.04 | 0.00 | 0.00 | -0.99 | 0.32 | 0.38 | 51.30 | 0.00 | 0.00 | 25.60 | 0.00 | 0.00 | 15.32 | 0.00 | 0.00 | 6-32 | 5.5-40.7 | 6-34.9 | 20.2-22.8 | NA | NA |
| Superior Temporal Thickness | R | -1.27 | 0.20 | 0.26 | -0.88 | 0.38 | 0.44 | 30.31 | 0.00 | 0.00 | 20.65 | 0.00 | 0.00 | 12.80 | 0.00 | 0.00 | 6-22.7 | 5.5-29.4 | 6-21.4 | NA | NA | NA |
| Temporal Pole Thickness | L | 0.86 | 0.39 | 0.45 | -1.04 | 0.30 | 0.36 | 0.12 | 0.73 | 0.78 | 2.31 | 0.13 | 0.18 | 0.93 | 0.34 | 0.39 | NA | NA | NA | NA | NA | NA |
| Temporal Pole Thickness | R | 1.00 | 0.32 | 0.38 | -5.13 | 0.00 | 0.00 | 0.40 | 0.53 | 0.58 | 5.25 | 0.02 | 0.03 | 0.86 | 0.32 | 0.38 | NA | 5.5-49.5 | NA | 6-17.5; 18.4-49.5 | NA | NA |
| Transverse Temporal Thickness | L | -0.45 | 0.65 | 0.70 | -1.18 | 0.24 | 0.29 | 31.29 | 0.00 | 0.00 | 21.07 | 0.00 | 0.00 | 8.28 | 0.00 | 0.00 | 6-25.4 | 5.5-24.7 | 6-19.7 | NA | NA | NA |
| Transverse Temporal Thickness | R | 2.77 | 0.01 | 0.01 | 0.30 | 0.76 | 0.80 | 25.70 | 0.00 | 0.00 | 13.65 | 0.00 | 0.00 | 12.80 | 0.00 | 0.00 | 6-22.1 | 5.5-24.3 | 6-19.7 | NA | NA | NA |
| Inferior Parietal Thickness | L | 2.74 | 0.01 | 0.01 | -2.53 | 0.01 | 0.02 | 73.35 | 0.00 | 0.00 | 51.85 | 0.00 | 0.00 | 16.32 | 0.00 | 0.00 | 6-26.1 | 5.5-33.1 | 6-23.2 | NA | NA | NA |
| Inferior Parietal Thickness | R | 3.96 | 0.00 | 0.00 | -1.65 | 0.10 | 0.14 | 73.65 | 0.00 | 0.00 | 50.53 | 0.00 | 0.00 | 18.09 | 0.00 | 0.00 | 6-23.6 | 5.5-35.1 | 6-36 | 13.9-21.1; 35.3-41.5 | 6-10.8; 14.8-25.1 | 6-11.7 |
| Isthmus Cingulate Thickness | L | 0.91 | 0.36 | 0.42 | -1.37 | 0.17 | 0.23 | 20.62 | 0.00 | 0.00 | 24.03 | 0.00 | 0.00 | 13.04 | 0.00 | 0.00 | 6-23.6 | 5.5-49.5 | 6-49.5 | 14.8-23.7; 39.7-49.5 | 6-9.1; 15.7-25.5 | NA |
| Isthmus Cingulate Thickness | R | -0.80 | 0.42 | 0.48 | -2.36 | 0.02 | 0.03 | 28.79 | 0.00 | 0.00 | 43.01 | 0.00 | 0.00 | 12.49 | 0.00 | 0.00 | 6-25 | 5.5-49.5 | 6-49.5 | 13.9-21.9; 36.2-49.5 | 6-9.9; 16.2-25.5 | NA |
| Postcentral Thickness | L | 10.77 | 0.00 | 0.00 | -4.45 | 0.00 | 0.00 | 40.96 | 0.00 | 0.00 | 21.48 | 0.00 | 0.00 | 9.34 | 0.00 | 0.00 | 6-20.5; 28.3-31.1 | 5.5-23.4 | 6-19 | 6-6.4; 15.7-19.3 | NA | NA |
| Postcentral Thickness | R | 8.55 | 0.00 | 0.00 | -3.36 | 0.00 | 0.00 | 35.77 | 0.00 | 0.00 | 16.13 | 0.00 | 0.00 | 12.27 | 0.00 | 0.00 | 6-19.9; 26.3-31.4; 45.7-49.5 | 5.5-20.3 | 6-18.8; 24.7-33.4 | NA | NA | 14.8-20.2 |
| Posterior Cingulate Thickness | L | -1.91 | 0.06 | 0.08 | 3.33 | 0.00 | 0.00 | 51.62 | 0.00 | 0.00 | 48.48 | 0.00 | 0.00 | 37.11 | 0.00 | 0.00 | 6-30.5 | 5.5-47.1 | 6-49.5 | 15.3-20.6; 35.7-49.5 | 6-11.7; 16.2-30.4; 43.7-49.5 | 6-13.9; 20.2-39.3 |
| Posterior Cingulate Thickness | R | -5.12 | 0.00 | 0.00 | 1.95 | 0.05 | 0.08 | 47.50 | 0.00 | 0.00 | 24.38 | 0.00 | 0.00 | 33.56 | 0.00 | 0.00 | 6-31.1 | 5.5-32.5 | 6-49.5 | NA | 6-10.4; 16.2-27.7 | 20.6-27.7 |
| Precuneus Thickness | L | 1.97 | 0.05 | 0.07 | -4.55 | 0.00 | 0.00 | 70.32 | 0.00 | 0.00 | 48.20 | 0.00 | 0.00 | 13.89 | 0.00 | 0.00 | 6-23.2 | 5.5-32.3 | 6-22.7 | NA | NA | NA |
| Precuneus Thickness | R | 4.40 | 0.00 | 0.00 | -7.15 | 0.00 | 0.00 | 68.21 | 0.00 | 0.00 | 40.09 | 0.00 | 0.00 | 8.63 | 0.00 | 0.00 | 6-22.7 | 5.5-24.5 | 6-20.8 | NA | NA | NA |
| Superior Parietal Thickness | L | 4.11 | 0.00 | 0.00 | -2.56 | 0.01 | 0.02 | 54.73 | 0.00 | 0.00 | 32.59 | 0.00 | 0.00 | 15.83 | 0.00 | 0.00 | 6-21.4 | 5.5-24.3 | 6-19.7 | 16.2-17.5 | NA | NA |
| Superior Parietal Thickness | R | 4.17 | 0.00 | 0.00 | -3.05 | 0.00 | 0.00 | 51.32 | 0.00 | 0.00 | 30.90 | 0.00 | 0.00 | 15.83 | 0.00 | 0.00 | 6-21; 28.5-31.6 | 5.5-24.1 | 6-20.1 | 14.4-18.4 | NA | NA |
| Supramarginal Thickness | L | 7.32 | 0.00 | 0.00 | -1.35 | 0.18 | 0.23 | 58.96 | 0.00 | 0.00 | 45.05 | 0.00 | 0.00 | 24.19 | 0.00 | 0.00 | 6-23.2 | 5.5-35.8 | 6-49.5 | 13.5-21.1; 33.9-44.6 | 6-10.8; 14.4-25.5 | 6-11.7; 20.2-24.6 |
| Supramarginal Thickness | R | 9.14 | 0.00 | 0.00 | -5.47 | 0.00 | 0.00 | 62.62 | 0.00 | 0.00 | 37.55 | 0.00 | 0.00 | 10.86 | 0.00 | 0.00 | 6-22.1 | 5.5-34 | 6-21.9 | 6-8.6; 13.5-22.4; 33.9-38.8 | NA | NA |
| Cuneus Thickness | R | 8.09 | 0.00 | 0.00 | -4.83 | 0.00 | 0.00 | 43.50 | 0.00 | 0.00 | 24.43 | 0.00 | 0.00 | 10.30 | 0.00 | 0.00 | 6-20.8 | 5.5-20.3; 28.3-30.9 | 6-19.4; 28.7-34 | NA | NA | NA |
| Lateral occipital Thickness | R | 3.22 | 0.00 | 0.00 | -2.83 | 0.00 | 0.01 | 37.70 | 0.00 | 0.00 | 28.29 | 0.00 | 0.00 | 17.39 | 0.00 | 0.00 | 6-23 | 5.5-24.7 | 6-20.5 | 39.3-46.4 | NA | 15.7-22.4 |
| Lingual Thickness | R | 7.53 | 0.00 | 0.00 | -4.79 | 0.00 | 0.00 | 38.28 | 0.00 | 0.00 | 18.56 | 0.00 | 0.00 | 8.60 | 0.00 | 0.00 | 6-22.3 | 5.5-22.7 | 6-21.2 | 17.9-22.8 | NA | NA |
| Pericalcarine Thickness | R | 11.84 | 0.00 | 0.00 | -5.78 | 0.00 | 0.00 | 13.77 | 0.00 | 0.00 | 8.63 | 0.00 | 0.00 | 6.16 | 0.01 | 0.02 | 6-18.8 | 5.5-19.4 | 6-19.9 | NA | NA | NA |
|  |  |  |  |  |  |  |  |  |  |  |  |  |  |  |  |  |  |  |  |  |  |  |
| Caudal Anterior Cingulate Area | R | -25.83 | 0.00 | 0.00 | 6.88 | 0.00 | 0.00 | 0.10 | 0.76 | 0.80 | 4.15 | 0.04 | 0.06 | 0.19 | 0.66 | 0.71 | NA | NA | NA | NA | NA | NA |
| Caudal middle frontal Area | R | -11.86 | 0.00 | 0.00 | 3.88 | 0.00 | 0.00 | 1.75 | 0.11 | 0.15 | 5.58 | 0.02 | 0.03 | 1.74 | 0.19 | 0.24 | NA | 5.5-49.5 | NA | NA | NA | NA |
| Frontal Pole Area | R | -0.90 | 0.37 | 0.43 | 3.32 | 0.00 | 0.00 | 1.54 | 0.22 | 0.27 | 1.46 | 0.23 | 0.28 | 5.78 | 0.02 | 0.03 | NA | NA | 6-49.5 | NA | NA | NA |
| Medial Orbitofrontal Area | R | -5.63 | 0.00 | 0.00 | 3.32 | 0.00 | 0.00 | 2.13 | 0.22 | 0.27 | 2.69 | 0.03 | 0.05 | 1.04 | 0.30 | 0.36 | NA | 15.7-28.1 | NA | 23.7-31.7 | NA | NA |
| Lateral Orbitofrontal Area | R | -5.73 | 0.00 | 0.00 | 4.05 | 0.00 | 0.00 | 0.60 | 0.44 | 0.49 | 1.00 | 0.32 | 0.38 | 0.02 | 0.90 | 0.91 | NA | NA | NA | NA | NA | NA |
| Paracentral Area | R | -26.22 | 0.00 | 0.00 | 13.00 | 0.00 | 0.00 | 7.11 | 0.01 | 0.02 | 0.77 | 0.38 | 0.44 | 0.20 | 0.83 | 0.85 | 6-20.1 | NA | NA | 6-6.8; 17.5-24.2 | NA | NA |
| Pars opercularis Area | R | -6.06 | 0.00 | 0.00 | -3.35 | 0.00 | 0.00 | 4.09 | 0.04 | 0.06 | 2.58 | 0.05 | 0.07 | 0.00 | 1.00 | 1.00 | NA | NA | NA | NA | NA | NA |
| Pars orbitalis Area | R | -0.82 | 0.41 | 0.46 | 3.55 | 0.00 | 0.00 | 0.00 | 0.96 | 0.96 | 2.30 | 0.14 | 0.18 | 0.06 | 0.81 | 0.84 | NA | NA | NA | NA | NA | NA |
| Pars triangularis Area | R | -23.01 | 0.00 | 0.00 | 1.89 | 0.06 | 0.09 | 1.07 | 0.28 | 0.34 | 5.66 | 0.00 | 0.00 | 0.78 | 0.53 | 0.59 | NA | 16.3-32.7 | NA | 8.6-13.5; 33.1-39.7 | NA | 11.3-14.8; 25.1-37.5 |
| Precentral Area | R | 1.00 | 0.32 | 0.38 | 8.96 | 0.00 | 0.00 | 0.08 | 0.78 | 0.81 | 2.16 | 0.07 | 0.10 | 1.04 | 0.31 | 0.37 | NA | NA | NA | NA | NA | NA |
| Rostral anterior cingulate Area | R | -22.84 | 0.00 | 0.00 | 4.83 | 0.00 | 0.00 | 0.78 | 0.38 | 0.44 | 0.12 | 0.73 | 0.77 | 0.05 | 0.83 | 0.85 | NA | NA | NA | NA | NA | NA |
| Rostral middle frontal Area | R | -35.38 | 0.00 | 0.00 | 1.42 | 0.16 | 0.21 | 9.45 | 0.00 | 0.00 | 15.98 | 0.00 | 0.00 | 2.10 | 0.13 | 0.18 | 6-49.5 | 5.5-49.5 | NA | NA | NA | 47.3-49.5 |
| Superior frontal Area | R | -20.72 | 0.00 | 0.00 | 5.15 | 0.00 | 0.00 | 0.47 | 0.67 | 0.72 | 7.61 | 0.00 | 0.00 | 0.69 | 0.41 | 0.46 | NA | 17.2-34 | NA | 10.4-18.4; 24.6-42.8 | NA | 12.6-19.7; 27.3-40.2 |
| Inferior parietal Area | L | -22.77 | 0.00 | 0.00 | -14.07 | 0.00 | 0.00 | 9.67 | 0.00 | 0.00 | 5.51 | 0.02 | 0.03 | 0.69 | 0.41 | 0.46 | 6-31.6 | 5.5-49.5 | NA | NA | 6-9.5; 19.3-26.8 | NA |
| Inferior parietal Area | R | -13.57 | 0.00 | 0.00 | 3.73 | 0.00 | 0.00 | 4.11 | 0.01 | 0.02 | 2.44 | 0.12 | 0.16 | 1.76 | 0.19 | 0.24 | 6-23.9 | NA | NA | NA | NA | NA |
| Isthmus Cingulate Area | L | -11.65 | 0.00 | 0.00 | 12.62 | 0.00 | 0.00 | 8.77 | 0.00 | 0.00 | 3.12 | 0.03 | 0.05 | 2.39 | 0.12 | 0.17 | 6-27.4 | NA | NA | NA | NA | NA |
| Isthmus Cingulate Area | R | -6.26 | 0.00 | 0.00 | 11.75 | 0.00 | 0.00 | 10.44 | 0.00 | 0.00 | 6.62 | 0.00 | 0.00 | 1.01 | 0.35 | 0.41 | 6-49.5 | 5.5-20.5 | NA | 15.3-22.8 | NA | NA |
| Postcentral Area | L | -31.27 | 0.00 | 0.00 | 0.20 | 0.84 | 0.86 | 7.45 | 0.00 | 0.01 | 2.91 | 0.03 | 0.05 | 2.35 | 0.13 | 0.17 | 6-21 | NA | NA | 6-6.8; 15.3-21.1 | 17.5-20.6 | NA |
| Postcentral Area | R | -36.88 | 0.00 | 0.00 | -4.16 | 0.00 | 0.00 | 7.18 | 0.01 | 0.01 | 2.58 | 0.04 | 0.05 | 0.02 | 0.90 | 0.91 | 6-21.2 | NA | NA | NA | 6-9.9; 17.9-27.7 | 23.3-32.2 |
| Posterior Cingulate Area | L | -26.03 | 0.00 | 0.00 | 5.60 | 0.00 | 0.00 | 6.96 | 0.00 | 0.00 | 5.11 | 0.01 | 0.01 | 4.30 | 0.11 | 0.14 | 6-31.4 | 5.5-32.3 | NA | NA | 16.2-19.7; 41.9-49.5 | 15.7-21.1 |
| Posterior Cingulate Area | R | -23.66 | 0.00 | 0.00 | 1.33 | 0.19 | 0.24 | 9.17 | 0.00 | 0.00 | 4.76 | 0.00 | 0.01 | 4.83 | 0.02 | 0.03 | 6-41.5 | 5.5-21.2 | 6-22.3; 36.7-43.3 | NA | 20.2-23.3; 44.6-49.5 | NA |
| Precuneus Area | L | -67.12 | 0.00 | 0.00 | 10.70 | 0.00 | 0.00 | 8.85 | 0.00 | 0.00 | 11.68 | 0.00 | 0.00 | 6.27 | 0.01 | 0.01 | 6-22.7 | 5.5-49.5 | 6-49.5 | 6-9.1; 16.2-25.1 | 16.2-22.4 | NA |
| Precuneus Area | R | -67.25 | 0.00 | 0.00 | 13.48 | 0.00 | 0.00 | 7.08 | 0.00 | 0.00 | 8.78 | 0.00 | 0.01 | 12.16 | 0.00 | 0.00 | 6-23.2 | 5.5-49.5 | 6-32.3 | 6-9.9; 17.1-25.9 | NA | 6-13.5; 18.8-38.4 |
| Cuneus Area | L | -58.46 | 0.00 | 0.00 | 6.85 | 0.00 | 0.00 | 0.95 | 0.40 | 0.46 | 2.03 | 0.16 | 0.21 | 0.74 | 0.39 | 0.45 | NA | NA | NA | NA | NA | NA |
| Cuneus Area | R | -52.96 | 0.00 | 0.00 | 2.36 | 0.02 | 0.03 | 0.08 | 0.79 | 0.82 | 1.47 | 0.23 | 0.28 | 8.34 | 0.00 | 0.01 | NA | NA | 6-49.5 | NA | 6-17.1; 18.8-49.5 | 6-17.5; 18.4-49.5 |
| Superior Parietal Area | L | -63.44 | 0.00 | 0.00 | 0.43 | 0.67 | 0.71 | 18.39 | 0.00 | 0.00 | 3.62 | 0.01 | 0.02 | 2.06 | 0.16 | 0.21 | 6-22.7 | 10.1-24.5 | NA | 6-5.9 | NA | NA |
| Superior Parietal Area | R | -46.09 | 0.00 | 0.00 | 3.75 | 0.00 | 0.00 | 12.85 | 0.00 | 0.00 | 11.40 | 0.00 | 0.00 | 0.15 | 0.70 | 0.74 | 6-21.9 | 5.5-49.5 | NA | 16.6-21.1 | 6-13.1; 17.5-33.5 | 6-17.5; 18.4-49.5 |
| Supramarginal Area | L | -11.29 | 0.00 | 0.00 | 8.49 | 0.00 | 0.00 | 5.36 | 0.01 | 0.02 | 5.48 | 0.02 | 0.03 | 9.52 | 0.00 | 0.00 | 6-21.9 | 5.5-49.5 | 6-49.5 | NA | 41.9-49.5 | NA |
| Supramarginal Area | R | -20.69 | 0.00 | 0.00 | 7.74 | 0.00 | 0.00 | 4.75 | 0.01 | 0.02 | 5.92 | 0.02 | 0.02 | 5.59 | 0.02 | 0.03 | 6-21.2 | 5.5-49.5 | 6-49.5 | NA | NA | NA |
| Lateral occipital Area | L | -24.59 | 0.00 | 0.00 | 4.25 | 0.00 | 0.00 | 1.08 | 0.21 | 0.26 | 0.73 | 0.40 | 0.45 | 1.09 | 0.22 | 0.27 | NA | NA | NA | NA | NA | NA |
| Lateral occipital Area | R | -16.50 | 0.00 | 0.00 | -1.33 | 0.18 | 0.24 | 1.11 | 0.30 | 0.36 | 4.21 | 0.04 | 0.06 | 1.06 | 0.30 | 0.36 | NA | NA | NA | NA | NA | NA |
| Lingual Area | L | -68.88 | 0.00 | 0.00 | 12.60 | 0.00 | 0.00 | 2.27 | 0.10 | 0.14 | 2.19 | 0.14 | 0.19 | 0.03 | 0.87 | 0.88 | NA | NA | NA | NA | NA | NA |
| Lingual Area | R | -58.28 | 0.00 | 0.00 | 8.88 | 0.00 | 0.00 | 0.37 | 0.55 | 0.60 | 0.08 | 0.79 | 0.82 | 1.61 | 0.21 | 0.26 | NA | NA | NA | NA | NA | NA |
| Pericalcarine Area | L | -45.01 | 0.00 | 0.00 | 0.51 | 0.61 | 0.67 | 0.08 | 0.78 | 0.81 | 0.25 | 0.62 | 0.67 | 3.22 | 0.06 | 0.08 | NA | NA | NA | NA | NA | NA |
| Pericalcarine Area | R | -50.49 | 0.00 | 0.00 | 0.07 | 0.95 | 0.95 | 1.63 | 0.20 | 0.26 | 0.21 | 0.77 | 0.81 | 3.52 | 0.03 | 0.04 | NA | NA | 30.5-49.5 | NA | 46.8-49.5 | 40.6-49.5 |

# e-Table 9. Group and Age Effects on Neurodevelopmental Trajectories in 22q11.2 CNV Carriers vs. Controls, covarying for IQ

|  |  | Parametric Effects of Group (Control as Reference) | | | | | | Non-parametric (smooth) Effects of Age | | | | | | | | | Age range(s) when significant change is taking place | | | Age range(s) when smoothed effects of age differed between groups | | |
| --- | --- | --- | --- | --- | --- | --- | --- | --- | --- | --- | --- | --- | --- | --- | --- | --- | --- | --- | --- | --- | --- | --- |
|  |  | **22qdel** | | | **22qdup** | | | **Controls** | | | **22qdel** | | | **22qdup** | | | **Controls** | **22qdel** | **22qdup** | **Controls vs 22qdel** | **Controls vs 22qdup** | **22qdel vs 22qdup** |
| **brain** | **hemisphere** | **T** | **p** | **q** | **T** | **p** | **q** | **F** | **p** | **q** | **F** | **p** | **q** | **F** | **p** | **q** |  |  |  |  |  |  |
| Mean Thickness |  | 7.34 | 0.00 | 0.00 | -3.92 | 0.00 | 0.00 | 100.35 | 0.00 | 0.00 | 62.22 | 0.00 | 0.00 | 32.09 | 0.00 | 0.00 | 6-24.1 | 5.5-33.1 | 6-21.9 | 13.9-20.6 ; 37.9-37.9 | NA | NA |
| Frontal Thickness | L | 6.56 | 0.00 | 0.00 | -2.33 | 0.02 | 0.03 | 107.47 | 0.00 | 0.00 | 62.91 | 0.00 | 0.00 | 22.31 | 0.00 | 0.00 | 6-25 | 5.5-28.9 | 6-23.2 | NA | NA | NA |
| Frontal Thickness | R | 5.32 | 0.00 | 0.00 | -3.60 | 0.00 | 0.00 | 81.58 | 0.00 | 0.00 | 58.54 | 0.00 | 0.00 | 17.10 | 0.00 | 0.00 | 6-27.2 | 5.5-31.6 | 6-23.2 | NA | NA | NA |
| Temporal Thickness | L | -0.60 | 0.55 | 0.56 | -1.80 | 0.07 | 0.09 | 28.43 | 0.00 | 0.00 | 41.93 | 0.00 | 0.00 | 15.93 | 0.00 | 0.00 | 6-30.9 | 5.5-49.5 | 6-39.8 | 15.3-21.1 ; 41.1-49.5 | NA | NA |
| Temporal Thickness | R | 2.11 | 0.04 | 0.05 | -3.85 | 0.00 | 0.00 | 28.62 | 0.00 | 0.00 | 48.88 | 0.00 | 0.00 | 13.40 | 0.00 | 0.00 | 6-26.7 | 5.5-49.5 | 6-22.7 | 13.9-21.9 ; 36.2-49.5 | NA | NA |
| Parietal Thickness | L | 3.82 | 0.00 | 0.00 | -2.66 | 0.01 | 0.01 | 90.72 | 0.00 | 0.00 | 64.08 | 0.00 | 0.00 | 17.23 | 0.00 | 0.00 | 6-24.7 | 5.5-40 | 6-22.5 | 13.5-22.4 ; 34.4-49.5 | NA | 37.9-41.1 |
| Parietal Thickness | R | 3.88 | 0.00 | 0.00 | -3.84 | 0.00 | 0.00 | 89.96 | 0.00 | 0.00 | 57.06 | 0.00 | 0.00 | 23.60 | 0.00 | 0.00 | 6-23 | 5.5-34.9 | 6-20.3 | 6-6.8 ; 13.1-21.9 ; 32.6-41.1 | NA | 12.6-15.3 ; 31.3-40.6 |
| Occipital Thickness | L | 7.20 | 0.00 | 0.00 | -4.65 | 0.00 | 0.00 | 48.08 | 0.00 | 0.00 | 19.95 | 0.00 | 0.00 | 11.73 | 0.00 | 0.00 | 6-21.2 | 5.5-20.1 | 6-19.9 | 20.6-21.5 | NA | NA |
| Occipital Thickness | R | 8.88 | 0.00 | 0.00 | -5.59 | 0.00 | 0.00 | 50.53 | 0.00 | 0.00 | 23.59 | 0.00 | 0.00 | 16.98 | 0.00 | 0.00 | 6-21.4 | 5.5-21.2 | 6-20.3 ; 30.3-33.4 | NA | NA | NA |
|  |  |  |  |  |  |  |  |  |  |  |  |  |  |  |  |  |  |  |  |  |  |  |
| Total Surface Area | T | -32.86 | 0.00 | 0.00 | 5.07 | 0.00 | 0.00 | 7.24 | 0.00 | 0.00 | 16.84 | 0.00 | 0.00 | 1.46 | 0.23 | 0.25 | 6-23.6 | 5.5-49.5 | NA | NA | NA | NA |
| Frontal Area | L | -14.88 | 0.00 | 0.00 | 3.79 | 0.00 | 0.00 | 3.83 | 0.03 | 0.03 | 12.79 | 0.00 | 0.00 | 0.52 | 0.47 | 0.49 | 6-26.1 | 5.5-49.5 | NA | NA | NA | NA |
| Frontal Area | R | -22.23 | 0.00 | 0.00 | 7.10 | 0.00 | 0.00 | 2.29 | 0.13 | 0.15 | 6.51 | 0.00 | 0.00 | 1.00 | 0.32 | 0.34 | NA | 14.8-32 | NA | 9.9-16.2 ; 23.3-39.3 | NA | 11.3-16.2 ; 24.2-37.5 |
| Temporal Area | L | -19.21 | 0.00 | 0.00 | 2.30 | 0.02 | 0.03 | 3.90 | 0.05 | 0.06 | 3.92 | 0.05 | 0.06 | 1.31 | 0.25 | 0.27 | NA | NA | NA | NA | NA | NA |
| Temporal Area | R | -34.56 | 0.00 | 0.00 | 4.14 | 0.00 | 0.00 | 2.70 | 0.11 | 0.13 | 7.29 | 0.01 | 0.01 | 2.41 | 0.12 | 0.14 | NA | 5.5-49.5 | NA | NA | NA | NA |
| Parietal Area | L | -24.14 | 0.00 | 0.00 | 1.27 | 0.21 | 0.23 | 14.62 | 0.00 | 0.00 | 4.78 | 0.00 | 0.01 | 2.16 | 0.14 | 0.16 | 6-21.2 | 8.8-25.6 | NA | NA | 6-10.4 ; 16.2-25.5 | NA |
| Parietal Area | R | -41.81 | 0.00 | 0.00 | 6.26 | 0.00 | 0.00 | 17.71 | 0.00 | 0.00 | 6.91 | 0.00 | 0.00 | 2.26 | 0.08 | 0.09 | 6-23.4 | 5.5-31.1 | NA | 18.8-19.7 | 19.3-20.2 | NA |
| Occipital Area | L | -29.24 | 0.00 | 0.00 | 4.31 | 0.00 | 0.00 | 2.43 | 0.06 | 0.07 | 2.16 | 0.14 | 0.16 | 0.01 | 0.93 | 0.94 | NA | NA | NA | NA | NA | NA |
| Occipital Area | R | -47.13 | 0.00 | 0.00 | 2.55 | 0.01 | 0.02 | 0.00 | 0.98 | 0.98 | 1.32 | 0.34 | 0.35 | 1.55 | 0.21 | 0.24 | NA | NA | NA | NA | NA | NA |

# e-Table 10. Group and Age Effects on Neurodevelopmental Trajectories in 22q11.2 CNV Carriers vs. Controls, covarying for antipsychotic medication use

|  |  | Parametric Effects of Group (Control as Reference) | | | | | | Non-parametric (smooth) Effects of Age | | | | | | | | | Age range(s) when significant change is taking place | | | Age range(s) when smoothed effects of age differed between groups | | |
| --- | --- | --- | --- | --- | --- | --- | --- | --- | --- | --- | --- | --- | --- | --- | --- | --- | --- | --- | --- | --- | --- | --- |
|  |  | **22qdel** | | | **22qdup** | | | **Controls** | | | **22qdel** | | | **22qdup** | | | **Controls** | **22qdel** | **22qdup** | **Controls vs 22qdel** | **Controls vs 22qdup** | **22qdel vs 22qdup** |
| **brain** | **hemisphere** | **T** | **p** | **q** | **T** | **p** | **q** | **F** | **p** | **q** | **F** | **p** | **q** | **F** | **p** | **q** |  |  |  |  |  |  |
| Mean Thickness |  | 7.92 | 0.00 | 0.00 | -3.72 | 0.00 | 0.00 | 101.37 | 0.00 | 0.00 | 59.57 | 0.00 | 0.00 | 33.26 | 0.00 | 0.00 | 6-24.3 | 5.5-32.7 | 6-22.1 | 14.4-21.1 | NA | NA |
| Frontal Thickness | L | 7.04 | 0.00 | 0.00 | -2.11 | 0.04 | 0.05 | 109.04 | 0.00 | 0.00 | 59.09 | 0.00 | 0.00 | 22.75 | 0.00 | 0.00 | 6-25.2 | 5.5-28.7 | 6-23.9 | NA | NA | NA |
| Frontal Thickness | R | 5.73 | 0.00 | 0.00 | -3.44 | 0.00 | 0.00 | 81.96 | 0.00 | 0.00 | 49.65 | 0.00 | 0.00 | 17.37 | 0.00 | 0.00 | 6-27.2 | 5.5-31.4 | 6-23.6 | NA | NA | NA |
| Temporal Thickness | L | 0.28 | 0.78 | 0.80 | -1.41 | 0.16 | 0.18 | 28.83 | 0.00 | 0.00 | 36.34 | 0.00 | 0.00 | 20.20 | 0.00 | 0.00 | 6-30.9 | 5.5-49.5 | 6-49.5 | 15.7-21.5 | NA | NA |
| Temporal Thickness | R | 2.37 | 0.02 | 0.03 | -3.79 | 0.00 | 0.00 | 28.37 | 0.00 | 0.00 | 46.05 | 0.00 | 0.00 | 13.87 | 0.00 | 0.00 | 6-26.9 | 5.5-49.5 | 6-22.3 | 13.9-21.5 ; 36.6-49.5 | NA | NA |
| Parietal Thickness | L | 4.48 | 0.00 | 0.00 | -2.46 | 0.01 | 0.02 | 90.79 | 0.00 | 0.00 | 59.67 | 0.00 | 0.00 | 17.70 | 0.00 | 0.00 | 6-24.7 | 5.5-38.9 | 6-22.7 | 13.5-22.4 ; 34.8-48.2 | NA | NA |
| Parietal Thickness | R | 4.19 | 0.00 | 0.00 | -3.71 | 0.00 | 0.00 | 90.23 | 0.00 | 0.00 | 53.59 | 0.00 | 0.00 | 23.91 | 0.00 | 0.00 | 6-23 | 5.5-34.5 | 6-20.5 | 6-7.7 ; 13.1-21.9 ; 33.1-40.2 | NA | 13.1-14.8 ; 32.6-39.7 |
| Occipital Thickness | L | 7.18 | 0.00 | 0.00 | -4.61 | 0.00 | 0.00 | 47.29 | 0.00 | 0.00 | 19.40 | 0.00 | 0.00 | 12.49 | 0.00 | 0.00 | 6-21.2 | 5.5-20.1 | 6-19.9 | NA | NA | NA |
| Occipital Thickness | R | 9.06 | 0.00 | 0.00 | -5.50 | 0.00 | 0.00 | 49.82 | 0.00 | 0.00 | 22.49 | 0.00 | 0.00 | 18.10 | 0.00 | 0.00 | 6-21.4 | 5.5-21 | 6-20.3 ; 30-33.4 | NA | NA | 19.7-21.1 |
|  |  |  |  |  |  |  |  |  |  |  |  |  |  |  |  |  |  |  |  |  |  |  |
| Total Surface Area |  | -29.61 | 0.00 | 0.00 | 4.95 | 0.00 | 0.00 | 7.89 | 0.00 | 0.00 | 15.47 | 0.00 | 0.00 | 1.32 | 0.25 | 0.27 | 6-23.4 | 5.5-49.5 | NA | NA | 19.7-21.1 | NA |
| Frontal Area | L | -13.76 | 0.00 | 0.00 | 3.61 | 0.00 | 0.00 | 3.41 | 0.03 | 0.04 | 11.81 | 0.00 | 0.00 | 0.41 | 0.52 | 0.54 | 6-24.1 | 5.5-49.5 | NA | NA | NA | NA |
| Frontal Area | R | -19.41 | 0.00 | 0.00 | 7.08 | 0.00 | 0.00 | 2.36 | 0.13 | 0.15 | 6.20 | 0.00 | 0.00 | 0.92 | 0.34 | 0.36 | NA | 15-31.8 | NA | 10.4-16.2 ; 23.7-38.8 | NA | 11.7-16.2 ; 24.6-37.5 |
| Temporal Area | L | -18.10 | 0.00 | 0.00 | 2.21 | 0.03 | 0.04 | 3.98 | 0.05 | 0.06 | 3.90 | 0.05 | 0.06 | 1.25 | 0.26 | 0.28 | NA | NA | NA | NA | NA | NA |
| Temporal Area | R | -30.33 | 0.00 | 0.00 | 4.04 | 0.00 | 0.00 | 2.82 | 0.10 | 0.12 | 7.01 | 0.01 | 0.01 | 2.37 | 0.13 | 0.15 | NA | 5.5-49.5 | NA | NA | NA | NA |
| Parietal Area | L | -22.62 | 0.00 | 0.00 | 1.15 | 0.25 | 0.27 | 14.97 | 0.00 | 0.00 | 4.68 | 0.01 | 0.01 | 2.03 | 0.16 | 0.18 | 6-21.2 | 9.3-25.6 | NA | NA | 6-10.8 ; 16.2-25.9 | NA |
| Parietal Area | R | -36.48 | 0.00 | 0.00 | 7.00 | 0.00 | 0.00 | 18.09 | 0.00 | 0.00 | 6.35 | 0.00 | 0.00 | 4.09 | 0.04 | 0.06 | 6-23.6 | 5.5-31.6 | NA | 17.5-21.9 | 6-8.6 ; 16.6-25.5 | NA |
| Occipital Area | L | -26.58 | 0.00 | 0.00 | 4.36 | 0.00 | 0.00 | 2.82 | 0.04 | 0.06 | 1.41 | 0.24 | 0.26 | 0.02 | 0.90 | 0.91 | NA | NA | NA | NA | NA | NA |
| Occipital Area | R | -40.73 | 0.00 | 0.00 | 2.52 | 0.01 | 0.02 | 0.01 | 0.96 | 0.96 | 1.00 | 0.40 | 0.42 | 1.74 | 0.19 | 0.21 | NA | NA | NA | NA | NA | NA |

# e-Table 11a. Neurodevelopmental CT and SA trajectories in 22qDel-PS+ vs. 22qDel-PS-

|  |  | Parametric Effects of Group  (22qDel-PS- group as Reference) | | | Non-parametric (smooth) Effects of Age | | | | | | Age range(s) when significant change is taking place | | Age range(s) when smoothed effects of age were different in groups |
| --- | --- | --- | --- | --- | --- | --- | --- | --- | --- | --- | --- | --- | --- |
|  |  | **22qDel-PS+ Group** | | | **22qDel-PS-Group** | | | **22qDel-PS+ Group** | | | **22qDel-PS-Group** | **22qDel-PS+ Group** | **22qDel-PS+ vs  22qDel-PS-** |
| **brain** | **hemisphere** | **t** | **p** | **q** | **F** | **p** | **q** | **F** | **p** | **q** |  |  |  |
| Overall Mean Thickness |  | 0.13 | 0.16 | 0.16 | 26.14 | 0.00 | 0.00 | 23.11 | 0.00 | 0.00 | 7.3-28.5 | 7.3-42.7 | 7.3-12.7; 17.7-24.8 |
| Frontal Thickness | L | 0.03 | 0.04 | 0.04 | 24.20 | 0.00 | 0.00 | 12.42 | 0.00 | 0.00 | 7.3-27.4 | 7.3-42.7 | 7.3-13; 18.4-24.8 |
| Frontal Thickness | R | 0.82 | 0.85 | 0.85 | 24.33 | 0.00 | 0.00 | 8.93 | 0.00 | 0.00 | 7.3-28.6 | 7.3-32 | 20.5-23.8 |
| Temporal Thickness | L | 0.84 | 0.86 | 0.86 | 16.72 | 0.00 | 0.00 | 9.12 | 0.00 | 0.01 | 7.3-42.7 | 7.3-42.7 | NA |
| Temporal Thickness | R | 0.78 | 0.82 | 0.82 | 15.97 | 0.00 | 0.00 | 12.18 | 0.00 | 0.00 | 7.3-42.7 | 7.3-42.7 | NA |
| Parietal Thickness | L | 0.50 | 0.55 | 0.55 | 29.43 | 0.00 | 0.00 | 26.27 | 0.00 | 0.00 | 7.3-22.6; 26.9-33.6 | 7.3-42.7 | 7.3-13; 17.3-23.8 |
| Parietal Thickness | R | 0.92 | 0.92 | 0.92 | 22.62 | 0.00 | 0.00 | 27.44 | 0.00 | 0.00 | 7.3-29.5 | 7.3-42.7 | 18-23.4 |
| Occipital Thickness | L | 0.53 | 0.57 | 0.57 | 10.27 | 0.00 | 0.00 | 1.00 | 0.32 | 0.41 | 7.3-19.1 | NA | 7.3-13.4; 17.3-23.4 |
| Occipital Thickness | R | 0.00 | 0.00 | 0.00 | 8.15 | 0.00 | 0.00 | 1.89 | 0.17 | 0.25 | 7.3-19.9 | NA | 7.3-13; 17.7-23.8 |
|  |  |  |  |  |  |  |  |  |  |  |  |  |  |
| Total Surface Area |  | 0.00 | 0.00 | 0.00 | 5.54 | 0.00 | 0.01 | 8.74 | 0.00 | 0.01 | 7.3-29.4 | 7.3-42.7 | NA |
| Frontal Area | L | 0.09 | 0.12 | 0.12 | 12.89 | 0.00 | 0.00 | 10.82 | 0.00 | 0.01 | 7.3-42.7 | 7.3-42.7 | NA |
| Frontal Area | R | 0.00 | 0.01 | 0.01 | 4.41 | 0.01 | 0.02 | 3.67 | 0.02 | 0.03 | 7.3-27.8 | 7.3-12.5; 21.2-31.5 | 7.3-12.3; 17.3-24.8 |
| Temporal Area | L | 0.00 | 0.00 | 0.00 | 4.04 | 0.05 | 0.09 | 6.25 | 0.02 | 0.03 | NA | 7.3-42.7 | NA |
| Temporal Area | R | 0.10 | 0.13 | 0.13 | 4.63 | 0.01 | 0.02 | 1.60 | 0.21 | 0.30 | 7.3-27.8 | NA | NA |
| Parietal Area | L | 0.00 | 0.00 | 0.00 | 3.73 | 0.01 | 0.02 | 9.05 | 0.00 | 0.00 | 11.6-23.5 | 7.3-28.3 | NA |
| Parietal Area | R | 0.00 | 0.00 | 0.00 | 5.39 | 0.00 | 0.01 | 2.75 | 0.10 | 0.17 | 7.3-21.5 | NA | 18.4-23.4 |
| Occipital Area | L | 0.00 | 0.00 | 0.00 | 4.02 | 0.05 | 0.09 | 0.98 | 0.30 | 0.40 | NA | NA | NA |
| Occipital Area | R | 0.00 | 0.00 | 0.00 | 3.79 | 0.06 | 0.10 | 1.05 | 0.31 | 0.41 | NA | NA | NA |

# e-Table 11b. Neurodevelopmental CT trajectories in 22qDel-PS+ vs. 22qDel-PS- (without ICV adjustment)

|  |  | Parametric Effects of Group  (22qDel-PS- group as Reference) | | | Non-parametric (smooth) Effects of Age | | | | | | Age range(s) when significant change is taking place | | Age range(s) when smoothed effects of age were different in groups |
| --- | --- | --- | --- | --- | --- | --- | --- | --- | --- | --- | --- | --- | --- |
|  |  | **22qDel-PS+ Group** | | | **22qDel-PS-Group** | | | **22qDel-PS+ Group** | | | **22qDel-PS- Group** | **22qDel-PS+ Group** | **22qDel-PS+ vs  22qDel-PS-** |
| **brain** | **hemisphere** | **t** | **p** | **q** | **F** | **p** | **q** | **F** | **p** | **q** |  |  |  |
| Mean Thickness |  | -1.49 | 0.14 | 0.20 | 31.15 | 0.00 | 0.00 | 23.48 | 0.00 | 0.00 | 7.3-28.6 | 7.3-42.7 | 7.3-13.4 ; 17.7-25.5 |
| Frontal Thickness | L | -1.90 | 0.06 | 0.09 | 26.42 | 0.00 | 0.00 | 13.61 | 0.00 | 0.00 | 7.3-27.2 | 7.3-42.7 | 7.3-13.4 ; 18.4-25.2 |
| Frontal Thickness | R | 0.56 | 0.58 | 0.65 | 27.13 | 0.00 | 0.00 | 9.17 | 0.00 | 0.00 | 7.3-28.3 | 7.3-34 | 7.3-12.7 ; 19.5-25.5 |
| Temporal Thickness | L | 0.01 | 0.99 | 0.99 | 20.03 | 0.00 | 0.00 | 9.07 | 0.00 | 0.01 | 7.3-42.7 | 7.3-42.7 | NA |
| Temporal Thickness | R | 0.36 | 0.72 | 0.75 | 11.92 | 0.00 | 0.00 | 12.25 | 0.00 | 0.00 | 7.3-37 | 7.3-42.7 | NA |
| Parietal Thickness | L | 0.85 | 0.40 | 0.49 | 36.06 | 0.00 | 0.00 | 27.42 | 0.00 | 0.00 | 7.3-24.4 ; 25.3-33.3 | 7.3-42.7 | 7.3-13.4 ; 17.3-24.5 |
| Parietal Thickness | R | 0.42 | 0.68 | 0.73 | 25.89 | 0.00 | 0.00 | 26.83 | 0.00 | 0.00 | 7.3-29 | 7.3-42.7 | 7.3-10.9 ; 18-24.1 |
| Occipital Thickness | L | -0.78 | 0.44 | 0.52 | 11.44 | 0.00 | 0.00 | 0.99 | 0.32 | 0.42 | 7.3-19.2 | NA | 7.3-13.4 ; 17.3-23.4 |
| Occipital Thickness | R | -4.12 | 0.00 | 0.00 | 9.40 | 0.00 | 0.00 | 1.85 | 0.18 | 0.24 | 7.3-20.1 | NA | 7.3-13.4 ; 18-24.1 |

# e-Table 12. Individual ROI Results for 22qDel-PS+ vs. 22qDel-PS–

|  |  | Parametric Effects of Group  (22qDel-PS- Group as Reference) | | | Non-parametric (smooth) Effects of Age | | | | | | Age range(s) when significant change is taking place | | Age range(s) when smoothed effects of age were different in groups |
| --- | --- | --- | --- | --- | --- | --- | --- | --- | --- | --- | --- | --- | --- |
|  |  | **22qDel-PS+ Group** | | | **22qDel-PS- Group** | | | **22qDel-PS+ Group** | | | **22qDel-PS- Group** | **22qDel-PS+ Group** | **22qDel-PS+ vs  22qDel-PS-** |
| **Brain Region** | **Hemisphere** | **t** | **p** | **q** | **F** | **p** | **q** | **F** | **p** | **q** |  |  |  |
| Caudal Anterior Cingulate Thickness | L | -2.63 | 0.01 | 0.02 | 14.22 | 0.00 | 0.00 | 7.32 | 0.00 | 0.00 | 7.3-42.7 | 7.3-25.8 | NA |
| Caudal Anterior Cingulate Thickness | R | 0.47 | 0.64 | 0.70 | 9.66 | 0.00 | 0.00 | 5.18 | 0.03 | 0.05 | 13.7-26.5 | NA | NA |
| Caudal Middle Frontal Thickness | L | -1.29 | 0.20 | 0.27 | 11.79 | 0.00 | 0.00 | 5.03 | 0.01 | 0.02 | 7.3-24.9 | 7.3-30.1 | NA |
| Caudal Middle Frontal Thickness | R | -0.80 | 0.43 | 0.50 | 11.66 | 0.00 | 0.00 | 7.78 | 0.01 | 0.02 | 7.3-28.8 | 7.3-42.7 | NA |
| Frontal Pole Thickness | L | -0.38 | 0.70 | 0.76 | 6.18 | 0.00 | 0.00 | 0.81 | 0.37 | 0.46 | 7.3-20.3 | NA | 7.3-12.3; 18.4-24.8 |
| Frontal Pole Thickness | R | -1.12 | 0.27 | 0.34 | 1.58 | 0.14 | 0.20 | 3.20 | 0.08 | 0.12 | NA | NA | NA |
| Medial Orbitofrontal Thickness | L | -1.67 | 0.10 | 0.15 | 17.88 | 0.00 | 0.00 | 9.43 | 0.00 | 0.01 | 7.3-29 | 7.3-42.7 | 7.3-13; 19.5-25.9 |
| Medial Orbitofrontal Thickness | R | -1.05 | 0.30 | 0.37 | 22.16 | 0.00 | 0.00 | 18.42 | 0.00 | 0.00 | 7.3-35 | 7.3-30.8 | NA |
| Lateral Orbitofrontal Thickness | L | -1.24 | 0.22 | 0.29 | 20.64 | 0.00 | 0.00 | 7.97 | 0.00 | 0.00 | 7.3-28.6 | 7.3-33.1 | 21.6-23 |
| Lateral Orbitofrontal Thickness | R | -2.95 | 0.00 | 0.01 | 25.46 | 0.00 | 0.00 | 11.09 | 0.00 | 0.00 | 7.3-31.5 | 7.3-22.2; 27.9-28.3 | NA |
| Paracentral Thickness | L | -1.49 | 0.14 | 0.20 | 12.41 | 0.00 | 0.00 | 3.82 | 0.02 | 0.04 | 7.3-22.1 | 7.3-27.6 | 7.3-11.3; 19.1-22.7 |
| Paracentral Thickness | R | -1.97 | 0.05 | 0.09 | 11.72 | 0.00 | 0.00 | 3.83 | 0.08 | 0.12 | 7.3-22.8 | NA | 20.9-23 |
| Pars Opercularis Thickness | L | -1.86 | 0.07 | 0.11 | 17.25 | 0.00 | 0.00 | 9.79 | 0.00 | 0.01 | 7.3-30.1 | 7.3-42.7 | 20.5-24.8 |
| Pars Opercularis Thickness | R | -0.23 | 0.82 | 0.84 | 14.70 | 0.00 | 0.00 | 6.80 | 0.01 | 0.02 | 7.3-42.7 | 7.3-42.7 | NA |
| Pars Orbitalis Thickness | L | -3.19 | 0.00 | 0.01 | 12.08 | 0.00 | 0.00 | 7.66 | 0.01 | 0.02 | 7.3-26.2 | 7.3-42.7 | 19.5-24.8 |
| Pars Orbitalis Thickness | R | -0.26 | 0.79 | 0.83 | 13.98 | 0.00 | 0.00 | 5.06 | 0.01 | 0.02 | 8-28.1 | 7.3-29.9 | 22.3-25.9 |
| Pars Triangularis Thickness | L | -0.77 | 0.44 | 0.51 | 18.25 | 0.00 | 0.00 | 15.37 | 0.00 | 0.00 | 7.3-26.9 | 7.3-42.7 | 18.8-25.5 |
| Pars Triangularis Thickness | R | 0.50 | 0.62 | 0.68 | 10.48 | 0.00 | 0.00 | 5.57 | 0.02 | 0.04 | 7.3-27.8 | 7.3-42.7 | 20.2-23 |
| Precentral Thickness | L | -0.30 | 0.77 | 0.80 | 9.23 | 0.00 | 0.00 | 0.77 | 0.38 | 0.47 | 7.3-28.1 | NA | 7.3-14.5; 20.9-28.4 |
| Precentral Thickness | R | -2.98 | 0.00 | 0.01 | 9.73 | 0.00 | 0.00 | 1.81 | 0.10 | 0.15 | 7.3-32.5 | NA | NA |
| Rostral Anterior Cingulate Thickness | L | -1.84 | 0.07 | 0.11 | 5.40 | 0.00 | 0.01 | 8.71 | 0.00 | 0.01 | 7.3-28.6 | 7.3-42.7 | NA |
| Rostral Anterior Cingulate Thickness | R | 2.80 | 0.01 | 0.02 | 27.29 | 0.00 | 0.00 | 4.13 | 0.01 | 0.03 | 7.3-42.7 | 7.3-20.5 | NA |
| Rostral Middle Frontal Thickness | L | -2.53 | 0.01 | 0.03 | 14.96 | 0.00 | 0.00 | 6.80 | 0.00 | 0.01 | 7.3-24.2 | 7.3-25.4 | NA |
| Rostral Middle Frontal Thickness | R | 0.12 | 0.91 | 0.92 | 15.22 | 0.00 | 0.00 | 6.60 | 0.00 | 0.01 | 7.3-20.8 | 7.3-29.4 | 7.3-11.3; 17.3-22 |
| Superior Frontal Thickness | L | -2.04 | 0.05 | 0.08 | 19.32 | 0.00 | 0.00 | 6.19 | 0.01 | 0.01 | 7.3-29.5 | 7.3-32.9 | 7.3-13.4; 19.8-25.2 |
| Superior Frontal Thickness | R | -1.40 | 0.17 | 0.22 | 20.93 | 0.00 | 0.00 | 8.32 | 0.00 | 0.00 | 7.3-25.4 | 7.3-25.6 | NA |
| Inferior Parietal Thickness | L | -1.79 | 0.08 | 0.12 | 28.21 | 0.00 | 0.00 | 21.04 | 0.00 | 0.00 | 7.3-22.6 | 7.3-42.7 | 7.3-13.4; 17.3-25.2 |
| Inferior Parietal Thickness | R | -0.65 | 0.52 | 0.57 | 21.88 | 0.00 | 0.00 | 21.35 | 0.00 | 0.00 | 7.3-28.3 | 7.3-42.7 | 7.3-11.6; 18-24.1 |
| Isthmus Cingulate Thickness | L | -0.30 | 0.76 | 0.80 | 16.06 | 0.00 | 0.00 | 6.74 | 0.01 | 0.02 | 7.3-42.7 | 7.3-42.7 | NA |
| Isthmus Cingulate Thickness | R | 1.17 | 0.24 | 0.32 | 14.86 | 0.00 | 0.00 | 10.67 | 0.00 | 0.00 | 7.3-42.7 | 7.3-42.7 | NA |
| Postcentral Thickness | L | 0.79 | 0.43 | 0.50 | 7.07 | 0.00 | 0.00 | 5.43 | 0.02 | 0.04 | 7.3-21.7 | 7.3-42.7 | 19.1-22.3 |
| Postcentral Thickness | R | -0.81 | 0.42 | 0.50 | 5.42 | 0.00 | 0.00 | 4.02 | 0.05 | 0.08 | 7.3-19.1 | NA | 7.3-9.5; 17.7-21.6 |
| Posterior Cingulate Thickness | L | 1.86 | 0.07 | 0.11 | 22.29 | 0.00 | 0.00 | 12.74 | 0.00 | 0.00 | 7.3-31.8 | 7.3-29.5 | NA |
| Posterior Cingulate Thickness | R | 3.23 | 0.00 | 0.01 | 13.96 | 0.00 | 0.00 | 8.03 | 0.00 | 0.00 | 7.3-26.2 | 7.3-32.2 | NA |
| Precuneus Thickness | L | -0.15 | 0.88 | 0.90 | 21.44 | 0.00 | 0.00 | 11.82 | 0.00 | 0.00 | 7.3-23 | 7.3-42.7 | 7.3-13.8; 18-25.2 |
| Precuneus Thickness | R | -1.54 | 0.13 | 0.18 | 16.11 | 0.00 | 0.00 | 5.41 | 0.00 | 0.01 | 7.3-20.6 | 7.3-27.6 | 7.3-11.6; 18-22 |
| Superior Parietal Thickness | L | -0.46 | 0.65 | 0.70 | 16.55 | 0.00 | 0.00 | 13.22 | 0.00 | 0.00 | 7.3-25.4 | 7.3-42.7 | 7.3-12; 18.4-24.5 |
| Superior Parietal Thickness | R | -0.82 | 0.41 | 0.49 | 11.92 | 0.00 | 0.00 | 14.03 | 0.00 | 0.00 | 7.3-21.4 | 7.3-42.7 | 7.3-8.8; 17.3-22.7 |
| Supramarginal Thickness | L | 0.83 | 0.41 | 0.49 | 15.57 | 0.00 | 0.00 | 15.44 | 0.00 | 0.00 | 7.3-23.7 | 7.3-42.7 | 7.3-10.2; 18-23.4 |
| Supramarginal Thickness | R | -3.45 | 0.00 | 0.00 | 15.00 | 0.00 | 0.00 | 21.79 | 0.00 | 0.00 | 7.3-29.5 | 7.3-42.7 | NA |
| Cuneus Thickness | L | -1.13 | 0.26 | 0.34 | 9.10 | 0.00 | 0.00 | 3.07 | 0.08 | 0.13 | 7.3-19.6 | NA | 7.3-12.7; 17.3-23.4 |
| Cuneus Thickness | R | -4.43 | 0.00 | 0.00 | 11.30 | 0.00 | 0.00 | 2.18 | 0.15 | 0.20 | 7.3-19.4 | NA | 7.3-13; 16.6-24.1 |
| Lateral Occipital Thickness | L | -1.13 | 0.26 | 0.34 | 14.51 | 0.00 | 0.00 | 2.25 | 0.14 | 0.20 | 7.3-21.2 | NA | 7.3-14.1; 18-26.2 |
| Lateral Occipital Thickness | R | -2.48 | 0.02 | 0.03 | 11.13 | 0.00 | 0.00 | 7.31 | 0.01 | 0.02 | 7.3-27.4 | 7.3-42.7 | 19.8-22.7 |
| Lingual Thickness | L | -0.78 | 0.44 | 0.51 | 5.05 | 0.00 | 0.01 | 2.58 | 0.11 | 0.16 | 7.3-19.6 | NA | 20.2-20.5 |
| Lingual Thickness | R | -0.87 | 0.39 | 0.47 | 13.63 | 0.00 | 0.00 | 2.51 | 0.12 | 0.17 | 7.3-42.7 | NA | NA |
| Pericalcarine Thickness | L | -0.21 | 0.83 | 0.86 | 3.05 | 0.02 | 0.04 | 0.16 | 0.69 | 0.75 | 7.3-17.3; 23.5-25.4 | NA | 7.3-11.6; 17.3-22 |
| Pericalcarine Thickness | R | -4.43 | 0.00 | 0.00 | 3.34 | 0.02 | 0.03 | 0.56 | 0.46 | 0.52 | 7.3-18.7 | NA | 7.3-14.1; 19.5-24.5 |
|  |  |  |  |  |  |  |  |  |  |  |  |  |  |
| Caudal Anterior Cingulate Area | R | 1.48 | 0.14 | 0.20 | 1.49 | 0.23 | 0.30 | 0.70 | 0.46 | 0.52 | NA | NA | NA |
| Caudal Middle Frontal Area | R | 1.08 | 0.28 | 0.36 | 2.92 | 0.09 | 0.14 | 4.47 | 0.04 | 0.07 | NA | NA | NA |
| Frontal Pole Area | R | -2.05 | 0.04 | 0.08 | 3.42 | 0.04 | 0.07 | 3.83 | 0.06 | 0.10 | NA | NA | NA |
| Medial Orbitofrontal Area | R | 1.71 | 0.09 | 0.14 | 1.96 | 0.21 | 0.28 | 0.14 | 0.71 | 0.76 | NA | NA | NA |
| Lateral Orbitofrontal Area | R | 0.06 | 0.95 | 0.96 | 0.45 | 0.48 | 0.54 | 1.67 | 0.12 | 0.17 | NA | NA | NA |
| Paracentral Area | R | -3.47 | 0.00 | 0.00 | 0.81 | 0.37 | 0.46 | 2.94 | 0.08 | 0.13 | NA | NA | NA |
| Pars Opercularis Area | R | 0.37 | 0.71 | 0.76 | 1.82 | 0.14 | 0.20 | 1.57 | 0.23 | 0.30 | NA | NA | NA |
| Pars Orbitalis Area | R | -4.55 | 0.00 | 0.00 | 6.17 | 0.02 | 0.03 | 0.71 | 0.49 | 0.55 | 7.3-42.7 | NA | 7.3-9.5 |
| Pars Triangularis Area | R | -6.09 | 0.00 | 0.00 | 3.50 | 0.02 | 0.04 | 4.07 | 0.05 | 0.08 | 7.3-27.4 | NA | NA |
| Precentral Area | R | -3.38 | 0.00 | 0.00 | 0.65 | 0.41 | 0.49 | 0.01 | 0.94 | 0.95 | NA | NA | NA |
| Rostral Anterior Cingulate Area | R | 0.02 | 0.99 | 0.99 | 1.17 | 0.37 | 0.46 | 1.30 | 0.17 | 0.23 | NA | NA | NA |
| Rostral Middle Frontal Area | R | -8.60 | 0.00 | 0.00 | 3.47 | 0.03 | 0.06 | 1.01 | 0.37 | 0.46 | NA | NA | NA |
| Superior Frontal Area | R | 1.40 | 0.17 | 0.22 | 15.14 | 0.00 | 0.00 | 3.15 | 0.04 | 0.08 | 7.3-42.7 | NA | 7.3-10.9; 18.8-23.4 |
| Inferior Parietal Area | R | 6.42 | 0.00 | 0.00 | 3.44 | 0.04 | 0.07 | 2.94 | 0.09 | 0.14 | NA | NA | NA |
| Isthmus Cingulate Area | R | 2.86 | 0.01 | 0.01 | 0.74 | 0.45 | 0.52 | 0.42 | 0.52 | 0.57 | NA | NA | NA |
| Postcentral Area | R | 3.50 | 0.00 | 0.00 | 3.97 | 0.01 | 0.02 | 4.50 | 0.04 | 0.07 | 7.3-24.4 | NA | NA |
| Posterior Cingulate Area | R | 1.91 | 0.06 | 0.10 | 5.51 | 0.00 | 0.01 | 0.09 | 0.76 | 0.80 | 7.3-18.5; 26.7-37.5 | NA | 7.3-9.1; 17-23.4 |
| Precuneus Area | R | 16.21 | 0.00 | 0.00 | 3.20 | 0.03 | 0.05 | 3.18 | 0.08 | 0.12 | NA | NA | NA |
| Superior Parietal Area | R | 9.92 | 0.00 | 0.00 | 4.30 | 0.01 | 0.03 | 2.60 | 0.04 | 0.06 | 7.3-30.4 | NA | NA |
| Supramarginal Area | R | -0.02 | 0.98 | 0.99 | 3.46 | 0.03 | 0.06 | 0.44 | 0.51 | 0.57 | NA | NA | NA |

# e-Table 13. Neurodevelopmental CT and SA trajectories in 22qDel-PS+ vs. 22qDel-PS-, covarying for antipsychotic medication use.

|  |  | Parametric Effects of Group  (22qDel-PS- group as Reference) | | | Non-parametric (smooth) Effects of Age | | | | | | Age range(s) when significant change is taking place | | Age range(s) when smoothed effects of age were different in groups |
| --- | --- | --- | --- | --- | --- | --- | --- | --- | --- | --- | --- | --- | --- |
|  |  | **22qDel-PS+ Group** | | | **22qDel-PS- Group** | | | **22qDel-PS+ Group** | | | **22qDel-PS- Group** | **22qDel-PS+ Group** | **22qDel-PS+ vs  22qDel-PS-** |
| **brain** | **hemisphere** | **t** | **p** | **q** | **F** | **p** | **q** | **F** | **p** | **q** |  |  |  |
| Overall Mean Thickness |  | -0.73 | 0.46 | 0.50 | 25.58 | 0.00 | 0.00 | 18.10 | 0.00 | 0.00 | 7.3-28.5 | 7.3-42.7 | 7.3-13; 18-24.8 |
| Frontal Thickness | L | -1.61 | 0.11 | 0.15 | 23.67 | 0.00 | 0.00 | 7.63 | 0.00 | 0.00 | 7.3-27.2 | 7.3-32.4 | 7.3-13; 19.1-24.5 |
| Frontal Thickness | R | 0.37 | 0.71 | 0.73 | 23.79 | 0.00 | 0.00 | 11.64 | 0.00 | 0.00 | 7.3-29 | 7.3-28.8 | NA |
| Temporal Thickness | L | 1.03 | 0.31 | 0.36 | 16.65 | 0.00 | 0.00 | 6.41 | 0.01 | 0.02 | 7.3-42.7 | 7.3-42.7 | NA |
| Temporal Thickness | R | 0.45 | 0.65 | 0.68 | 16.12 | 0.00 | 0.00 | 11.04 | 0.00 | 0.00 | 7.3-42.7 | 7.3-42.7 | NA |
| Parietal Thickness | L | 1.20 | 0.24 | 0.29 | 28.98 | 0.00 | 0.00 | 22.98 | 0.00 | 0.00 | 7.3-22.4; 26.9-34.1 | 7.3-42.7 | 7.3-13; 17.3-24.1 |
| Parietal Thickness | R | 0.25 | 0.81 | 0.81 | 22.12 | 0.00 | 0.00 | 24.68 | 0.00 | 0.00 | 7.3-29.5 | 7.3-42.7 | 7.3-7.7; 18-23.4 |
| Occipital Thickness | L | -0.50 | 0.62 | 0.66 | 10.09 | 0.00 | 0.00 | 0.88 | 0.35 | 0.40 | 7.3-19.1 | NA | 7.3-13.4; 17.3-23.4 |
| Occipital Thickness | R | -3.09 | 0.00 | 0.01 | 7.92 | 0.00 | 0.00 | 1.58 | 0.21 | 0.27 | 7.3-19.9 | NA | 7.3-13; 18-23.8 |
|  |  |  |  |  |  |  |  |  |  |  |  |  |  |
| Total Surface Area |  | 3.03 | 0.00 | 0.01 | 5.51 | 0.00 | 0.01 | 8.42 | 0.01 | 0.01 | 7.3-29.4 | 7.3-42.7 | NA |
| Frontal Area | L | -2.00 | 0.05 | 0.08 | 12.93 | 0.00 | 0.00 | 10.64 | 0.00 | 0.00 | 7.3-42.7 | 7.3-42.7 | NA |
| Frontal Area | R | -4.00 | 0.00 | 0.00 | 4.49 | 0.01 | 0.01 | 3.85 | 0.01 | 0.02 | 7.3-27.9 | 7.3-12.1;21-32.2 | 7.3-12.3; 17.3-24.8 |
| Temporal Area | L | 4.20 | 0.00 | 0.00 | 3.83 | 0.06 | 0.08 | 5.57 | 0.02 | 0.03 | NA | 7.3-42.7 | NA |
| Temporal Area | R | 1.11 | 0.27 | 0.33 | 4.65 | 0.01 | 0.02 | 1.64 | 0.21 | 0.26 | 7.3-27.9 | NA | NA |
| Parietal Area | L | 8.25 | 0.00 | 0.00 | 3.75 | 0.01 | 0.02 | 9.69 | 0.00 | 0.00 | 11.4-23.5 | 7.3-28.6 | 17-22; 32-42.7 |
| Parietal Area | R | 7.93 | 0.00 | 0.00 | 5.37 | 0.00 | 0.01 | 2.72 | 0.10 | 0.14 | 7.3-21.4 | NA | 18.4-23.4 |
| Occipital Area | L | 5.59 | 0.00 | 0.00 | 3.59 | 0.06 | 0.09 | 0.66 | 0.42 | 0.47 | NA | NA | NA |
| Occipital Area | R | 6.67 | 0.00 | 0.00 | 3.47 | 0.07 | 0.09 | 0.63 | 0.43 | 0.47 | NA | NA | NA |

# e-Table 14. Neurodevelopmental CT and SA trajectories in 22qDel-PS+ vs. 22qDel-PS-, under 35 years old.

|  |  | Parametric Effects of Group  (22qDel-PS- Group as Reference) | | | Non-parametric (smooth) Effects of Age | | | | | | Age range(s) when significant change is taking place | | Age range(s) when smoothed effects of age differed between groups |
| --- | --- | --- | --- | --- | --- | --- | --- | --- | --- | --- | --- | --- | --- |
|  |  | **22qDel-PS+ Group** | | | **22qDel-PS- Group** | | | **22qDel-PS+ Group** | | | **22qDel-PS-Group** | **22qDel-PS+ Group** | **22qDel-PS+ vs  22qDel-PS-** |
| **brain** | **hemisphere** | **t** | **p** | **q** | **F** | **p** | **q** | **F** | **p** | **q** |  |  |  |
| Overall Mean Thickness |  | -1.32 | 0.19 | 0.24 | 25.23 | 0.00 | 0.00 | 18.61 | 0.00 | 0.00 | 7.3-23.6 | 7.3-27.8 | 7.3-12.3; 17.9-21.8 |
| Frontal Thickness | L | -2.20 | 0.03 | 0.05 | 24.41 | 0.00 | 0.00 | 18.10 | 0.00 | 0.00 | 7.3-23 | 7.3-27.8 | 7.3-12.3; 18.1-22.2 |
| Frontal Thickness | R | -0.45 | 0.66 | 0.70 | 26.51 | 0.00 | 0.00 | 20.37 | 0.00 | 0.00 | 7.3-27.8 | 7.3-27.8 | NA |
| Temporal Thickness | L | 0.04 | 0.97 | 0.97 | 8.71 | 0.00 | 0.01 | 3.64 | 0.06 | 0.09 | 7.3-27.8 | NA | NA |
| Temporal Thickness | R | 0.74 | 0.46 | 0.51 | 15.03 | 0.00 | 0.00 | 4.02 | 0.03 | 0.05 | 7.3-27.8 | NA | NA |
| Parietal Thickness | L | 0.67 | 0.50 | 0.54 | 27.90 | 0.00 | 0.00 | 18.13 | 0.00 | 0.00 | 7.3-22.7 | 7.3-27.8 | 7.3-12.9; 17.5-22.7 |
| Parietal Thickness | R | 0.28 | 0.78 | 0.81 | 23.85 | 0.00 | 0.00 | 19.16 | 0.00 | 0.00 | 7.3-27 | 7.3-27.8 | NA |
| Occipital Thickness | L | -0.87 | 0.39 | 0.45 | 10.88 | 0.00 | 0.00 | 0.74 | 0.39 | 0.45 | 7.3-19.5 | NA | 7.3-13.5; 17.3-23.7 |
| Occipital Thickness | R | -3.36 | 0.00 | 0.00 | 10.67 | 0.00 | 0.00 | 1.85 | 0.18 | 0.23 | 7.3-20.6 | NA | 7.3-13.1; 17.9-22.9 |
|  |  |  |  |  |  |  |  |  |  |  |  |  |  |
| Total Surface Area |  | 4.53 | 0.00 | 0.00 | 17.35 | 0.00 | 0.00 | 8.61 | 0.00 | 0.01 | 7.3-27.8 | 7.3-27.8 | NA |
| Frontal Area | L | 0.26 | 0.80 | 0.81 | 18.83 | 0.00 | 0.00 | 9.96 | 0.00 | 0.01 | 7.3-27.8 | 7.3-27.8 | NA |
| Frontal Area | R | -1.30 | 0.20 | 0.24 | 13.30 | 0.00 | 0.00 | 4.22 | 0.01 | 0.02 | 7.3-27.8 | 20.8-27.8 | 7.3-14; 17.5-23.9 |
| Temporal Area | L | 4.04 | 0.00 | 0.00 | 4.22 | 0.04 | 0.07 | 7.41 | 0.01 | 0.02 | NA | 7.3-27.8 | NA |
| Temporal Area | R | 2.49 | 0.02 | 0.03 | 13.10 | 0.00 | 0.00 | 2.04 | 0.16 | 0.21 | 7.3-27.8 | NA | NA |
| Parietal Area | L | 10.67 | 0.00 | 0.00 | 12.14 | 0.00 | 0.00 | 10.36 | 0.00 | 0.00 | 7.3-27.8 | 7.3-27.8 | NA |
| Parietal Area | R | 8.37 | 0.00 | 0.00 | 4.46 | 0.01 | 0.02 | 2.16 | 0.15 | 0.20 | 9.9-20.8 | NA | 18.3-21.2 |
| Occipital Area | L | 3.81 | 0.00 | 0.00 | 3.13 | 0.05 | 0.08 | 1.42 | 0.32 | 0.38 | NA | NA | NA |
| Occipital Area | R | 5.54 | 0.00 | 0.00 | 2.01 | 0.15 | 0.20 | 0.71 | 0.43 | 0.49 | NA | NA | NA |

# e-Table 15. Neurodevelopmental CT and SA trajectories in 22qDel-PS+ vs. 22qDel-PS-, covarying for comorbid ASD diagnosis.

|  |  | Parametric Effects of Group  (22qDel-PS- group as Reference) | | | Non-parametric (smooth) Effects of Age | | | | | | Age range(s) when significant change is taking place | | Age range(s) when smoothed effects of age were different in groups |
| --- | --- | --- | --- | --- | --- | --- | --- | --- | --- | --- | --- | --- | --- |
|  |  | **22qDel-PS+ Group** | | | **22qDel-PS-** | | | **22qDel-PS+ Group** | | | **22qDel-PS-** | **22qDel-PS+ Group** | **22qDel-PS+ vs  22qDel-PS-** |
| **brain** | **hemisphere** | **t** | **p** | **q** | **F** | **p** | **q** | **F** | **p** | **q** |  |  |  |
| Mean Thickness |  | -0.47 | 0.64 | 0.67 | 26.20 | 0.00 | 0.00 | 24.47 | 0.00 | 0.00 | 7.3-29.4 | 7.3-42.7 | 7.3-13 ; 17.7-25.2 |
| Frontal Thickness | L | -1.04 | 0.30 | 0.33 | 25.42 | 0.00 | 0.00 | 20.06 | 0.00 | 0.00 | 7.3-28.1 | 7.3-42.7 | 7.3-13.8 ; 18-25.9 |
| Frontal Thickness | R | 0.60 | 0.55 | 0.58 | 24.71 | 0.00 | 0.00 | 20.37 | 0.00 | 0.00 | 7.3-29 | 7.3-42.7 | 7.3-13.4 ; 18.8-27 |
| Temporal Thickness | L | 0.41 | 0.68 | 0.70 | 18.57 | 0.00 | 0.00 | 10.28 | 0.00 | 0.00 | 7.3-42.7 | 7.3-42.7 | NA |
| Temporal Thickness | R | 0.65 | 0.52 | 0.56 | 15.04 | 0.00 | 0.00 | 12.85 | 0.00 | 0.00 | 7.3-42.7 | 7.3-42.7 | NA |
| Parietal Thickness | L | 1.48 | 0.14 | 0.19 | 29.53 | 0.00 | 0.00 | 27.69 | 0.00 | 0.00 | 7.3-22.8 ; 26.5-36.5 | 7.3-42.7 | 7.3-13.4 ; 17.3-24.1 |
| Parietal Thickness | R | 1.12 | 0.27 | 0.30 | 24.19 | 0.00 | 0.00 | 29.58 | 0.00 | 0.00 | 7.3-30.2 | 7.3-42.7 | 7.3-11.3 ; 18-24.1 |
| Occipital Thickness | L | 0.36 | 0.72 | 0.72 | 10.93 | 0.00 | 0.00 | 1.32 | 0.25 | 0.29 | 7.3-19.2 ; 37.7-42.7 | NA | 7.3-13.4 ; 17.3-23.4 |
| Occipital Thickness | R | -2.48 | 0.02 | 0.03 | 8.89 | 0.00 | 0.00 | 2.40 | 0.13 | 0.17 | 7.3-20.1 | NA | 7.3-13 ; 17.7-24.1 |
|  |  |  |  |  |  |  |  |  |  |  |  |  |  |
| Total Surface Area |  | 3.98 | 0.00 | 0.00 | 5.78 | 0.00 | 0.01 | 9.16 | 0.00 | 0.01 | 7.3-29.9 | 7.3-42.7 | NA |
| Frontal Area | L | -1.16 | 0.25 | 0.29 | 12.51 | 0.00 | 0.00 | 10.86 | 0.00 | 0.00 | 7.3-42.7 | 7.3-42.7 | NA |
| Frontal Area | R | -1.28 | 0.21 | 0.25 | 4.76 | 0.01 | 0.01 | 3.85 | 0.01 | 0.02 | 7.3-28.5 | 7.3-11.9 ; 21-31.8 | 7.3-12.7 ; 17.3-25.2 |
| Temporal Area | L | 2.88 | 0.01 | 0.01 | 3.63 | 0.06 | 0.09 | 6.05 | 0.02 | 0.03 | NA | 7.3-42.7 | NA |
| Temporal Area | R | 1.98 | 0.05 | 0.08 | 4.61 | 0.01 | 0.02 | 1.73 | 0.19 | 0.25 | 7.3-28.1 | NA | NA |
| Parietal Area | L | 9.20 | 0.00 | 0.00 | 3.91 | 0.01 | 0.02 | 9.68 | 0.00 | 0.00 | 9.8-23.8 | 7.3-28.6 | NA |
| Parietal Area | R | 9.09 | 0.00 | 0.00 | 5.67 | 0.00 | 0.00 | 1.95 | 0.12 | 0.17 | 7.3-21.7 | NA | 18.8-23.4 |
| Occipital Area | L | 5.44 | 0.00 | 0.00 | 5.40 | 0.02 | 0.04 | 1.67 | 0.20 | 0.25 | 7.3-42.7 | NA | NA |
| Occipital Area | R | 6.45 | 0.00 | 0.00 | 4.56 | 0.04 | 0.06 | 1.32 | 0.25 | 0.29 | NA | NA | NA |

# e-Table 16. Neurodevelopmental CT and SA trajectories in 22qDel-PS+ vs. 22qDel-PS- when Psychosis Spectrum status varies as a function of visit

|  |  | Parametric Effects of Group  (22qDel-PS- Group as Reference) | | | Non-parametric (smooth) Effects of Age | | | | | | Age range(s) when significant change is taking place | | Age range(s) when smoothed effects of age were different in groups |
| --- | --- | --- | --- | --- | --- | --- | --- | --- | --- | --- | --- | --- | --- |
|  |  | **22qDel-PS+ Group** | | | **22qDel-PS- Group** | | | **22qDel-PS+ Group** | | | **22qDel-PS-** | **22qDel-PS+ Group** | **22qDel-PS+ vs  22qDel-PS-** |
| **brain** | **hemisphere** | **t** | **p** | **q** | **F** | **p** | **q** | **F** | **p** | **q** |  |  |  |
| Overall Mean Thickness |  | 1.05 | 0.30 | 0.36 | 27.54 | 0.00 | 0.00 | 29.92 | 0.00 | 0.00 | 7.3-24.2 | 7.3-42.7 | 7.3-11.6; 16.6-23.8 |
| Frontal Thickness | L | 0.44 | 0.66 | 0.73 | 25.38 | 0.00 | 0.00 | 11.11 | 0.00 | 0.00 | 7.3-26.2 | 7.3-42.7 | 7.3-12; 18-23.8 |
| Frontal Thickness | R | 0.36 | 0.72 | 0.78 | 26.53 | 0.00 | 0.00 | 15.46 | 0.00 | 0.00 | 7.3-28.8 | 7.3-31.1 | 20.5-22.7 |
| Temporal Thickness | L | 1.81 | 0.08 | 0.11 | 17.74 | 0.00 | 0.00 | 10.06 | 0.00 | 0.01 | 7.3-42.7 | 7.3-42.7 | NA |
| Temporal Thickness | R | -0.18 | 0.86 | 0.87 | 21.90 | 0.00 | 0.00 | 14.79 | 0.00 | 0.00 | 7.3-42.7 | 7.3-42.7 | NA |
| Parietal Thickness | L | 2.46 | 0.02 | 0.03 | 29.62 | 0.00 | 0.00 | 36.96 | 0.00 | 0.00 | 7.3-21.5; 27.9-42.7 | 7.3-42.7 | 7.3-11.3; 15.9-22.7 |
| Parietal Thickness | R | 2.72 | 0.01 | 0.02 | 24.18 | 0.00 | 0.00 | 35.47 | 0.00 | 0.00 | 7.3-28.6 | 7.3-42.7 | 16.6-22.7; 40.2-42.7 |
| Occipital Thickness | L | -0.21 | 0.83 | 0.87 | 10.07 | 0.00 | 0.00 | 4.97 | 0.03 | 0.05 | 7.3-18.7; 35.9-42.7 | 7.3-42.7 | 7.3-11.6; 15.9-22 |
| Occipital Thickness | R | -0.86 | 0.39 | 0.47 | 8.08 | 0.00 | 0.00 | 6.85 | 0.01 | 0.02 | 7.3-19.1 | 7.3-42.7 | 7.3-10.5; 15.9-22.3 |
|  |  |  |  |  |  |  |  |  |  |  |  |  |  |
| Total Surface Area |  | 1.59 | 0.12 | 0.16 | 7.37 | 0.00 | 0.00 | 10.17 | 0.00 | 0.01 | 7.3-30.6 | 7.3-42.7 | NA |
| Frontal Area | L | 0.81 | 0.42 | 0.49 | 9.25 | 0.00 | 0.00 | 7.33 | 0.02 | 0.03 | 7.3-33.8 | 13.2-34.7 | NA |
| Frontal Area | R | 1.19 | 0.24 | 0.30 | 13.26 | 0.00 | 0.00 | 0.81 | 0.44 | 0.49 | 7.3-42.7 | NA | 7.3-13.4; 21.3-25.9 |
| Temporal Area | L | 1.25 | 0.22 | 0.28 | 6.32 | 0.01 | 0.03 | 6.47 | 0.01 | 0.03 | 7.3-42.7 | 7.3-42.7 | NA |
| Temporal Area | R | -0.16 | 0.87 | 0.87 | 5.20 | 0.01 | 0.02 | 2.50 | 0.12 | 0.16 | 7.3-29 | NA | NA |
| Parietal Area | L | 0.78 | 0.44 | 0.49 | 8.79 | 0.00 | 0.00 | 15.21 | 0.00 | 0.00 | 7.3-24.2 | 7.3-42.7 | 17.3-22; 32.7-42.7 |
| Parietal Area | R | -0.17 | 0.86 | 0.87 | 4.95 | 0.00 | 0.01 | 8.94 | 0.00 | 0.01 | 7.3-21.2 | 7.3-42.7 | 16.6-21.6; 34.5-42.7 |
| Occipital Area | L | 2.06 | 0.04 | 0.07 | 2.60 | 0.11 | 0.16 | 5.42 | 0.02 | 0.04 | NA | 7.3-42.7 | NA |
| Occipital Area | R | 2.06 | 0.04 | 0.07 | 2.57 | 0.11 | 0.16 | 4.32 | 0.04 | 0.07 | NA | NA | NA |

# e-Table 17a. Neurodevelopmental CT and SA trajectories in 22qDel-ASD vs. 22qDel-No ASD

|  |  | Parametric Effects of Group  (22qDel-No ASD as Reference) | | | Non-parametric (smooth) Effects of Age | | | | | | Age range(s) when significant change is taking place | | Age range(s) when smoothed effects of age differed between groups |
| --- | --- | --- | --- | --- | --- | --- | --- | --- | --- | --- | --- | --- | --- |
|  |  | **22qDel-ASD Group** | | | **22qDel-No ASD Group** | | | **22qDel-ASD Group** | | | **22qDel-No ASD Group** | **22qDel- ASD Group** | **22qDel-ASD vs 22qDel-No ASD** |
| **brain** | **hemisphere** | **t** | **p** | **q** | **F** | **p** | **q** | **F** | **p** | **q** |  |  |  |
| Overall Mean Thickness |  | -0.93 | 0.36 | 0.40 | 29.52 | 0.00 | 0.00 | 28.54 | 0.00 | 0.00 | 5.5-25.2 | 5.5-25.2 | NA |
| Mean Thickness | L | -0.67 | 0.50 | 0.54 | 32.04 | 0.00 | 0.00 | 22.91 | 0.00 | 0.00 | 5.5-25.2 | 5.5-22.2 | NA |
| Mean Thickness | R | -1.15 | 0.25 | 0.29 | 25.33 | 0.00 | 0.00 | 44.96 | 0.00 | 0.00 | 5.5-25.2 | 5.5-25.2 | NA |
| Frontal Thickness | L | -1.96 | 0.05 | 0.08 | 36.91 | 0.00 | 0.00 | 37.57 | 0.00 | 0.00 | 5.5-25.2 | 5.5-25.2 | NA |
| Frontal Thickness | R | -2.20 | 0.03 | 0.05 | 26.61 | 0.00 | 0.00 | 37.41 | 0.00 | 0.00 | 5.5-25.2 | 5.5-25.2 | NA |
| Temporal Thickness | L | -0.47 | 0.64 | 0.67 | 7.55 | 0.01 | 0.01 | 3.86 | 0.05 | 0.08 | 5.5-25.2 | NA | NA |
| Temporal Thickness | R | -0.77 | 0.44 | 0.49 | 9.94 | 0.00 | 0.01 | 7.50 | 0.01 | 0.01 | 5.5-25.2 | 5.5-25.2 | NA |
| Parietal Thickness | L | -1.29 | 0.20 | 0.24 | 29.41 | 0.00 | 0.00 | 42.66 | 0.00 | 0.00 | 5.5-25.2 | 5.5-25.2 | NA |
| Parietal Thickness | R | -1.55 | 0.12 | 0.16 | 23.37 | 0.00 | 0.00 | 26.12 | 0.00 | 0.00 | 5.5-25.2 | 5.5-25.2 | NA |
| Occipital Thickness | L | -0.01 | 0.99 | 0.99 | 13.19 | 0.00 | 0.00 | 10.27 | 0.00 | 0.00 | 5.5-25.2 | 5.5-16.9 | NA |
| Occipital Thickness | R | -0.61 | 0.54 | 0.57 | 7.77 | 0.00 | 0.00 | 12.96 | 0.00 | 0.00 | 5.5-17.8 | 5.5-22.2 | NA |
|  |  |  |  |  |  |  |  |  |  |  |  |  |  |
| Total Surface Area | T | 2.32 | 0.02 | 0.04 | 7.33 | 0.01 | 0.02 | 7.46 | 0.00 | 0.00 | 5.5-25.2 | 5.5-10.7 ; 12.8-19.5 | 5.5-7.5 ; 11.3-15.1 |
| Frontal Area | L | 2.23 | 0.03 | 0.05 | 11.31 | 0.00 | 0.00 | 7.07 | 0.00 | 0.00 | 5.5-25.2 | 5.5-11.4 ; 13.8-19.6 | 5.5-8.9 ; 12.5-15.7 |
| Frontal Area | R | 1.58 | 0.12 | 0.16 | 3.48 | 0.07 | 0.10 | 5.59 | 0.00 | 0.00 | NA | 5.5-10.8 ; 13.5-19.5 | 5.5-7.1 ; 11.7-15.1 |
| Temporal Area | L | 3.79 | 0.00 | 0.00 | 2.34 | 0.13 | 0.16 | 3.72 | 0.04 | 0.06 | NA | NA | NA |
| Temporal Area | R | 3.18 | 0.00 | 0.01 | 2.28 | 0.14 | 0.17 | 4.68 | 0.00 | 0.01 | NA | 5.5-9.9 ; 13-19.3 | 11.3-14.7 |
| Parietal Area | L | 2.28 | 0.03 | 0.05 | 8.39 | 0.01 | 0.01 | 7.32 | 0.00 | 0.00 | 5.5-25.2 | 5.5-10.2 ; 12.7-19.7 | 5.5-6.9 ; 11.5-15.1 |
| Parietal Area | R | 1.60 | 0.11 | 0.16 | 1.77 | 0.13 | 0.16 | 7.40 | 0.00 | 0.00 | NA | 5.5-9.3 ; 12.2-19.6 | 10.3-14.9 ; 18.9-21.5 |
| Occipital Area | L | -0.29 | 0.78 | 0.79 | 3.76 | 0.08 | 0.11 | 1.61 | 0.13 | 0.16 | NA | NA | NA |
| Occipital Area | R | 1.44 | 0.15 | 0.19 | 0.79 | 0.46 | 0.51 | 2.30 | 0.06 | 0.08 | NA | NA | NA |

# e-Table 17b. Neurodevelopmental CT trajectories in 22qDel-ASD vs. 22qDel-No ASD (without ICV adjustment)

|  |  | Parametric Effects of Group  (22qDel-No ASD as Reference) | | | Non-parametric (smooth) Effects of Age | | | | | | Age range(s) when significant change is taking place | | Age range(s) when smoothed effects of age differed between groups |
| --- | --- | --- | --- | --- | --- | --- | --- | --- | --- | --- | --- | --- | --- |
|  |  | **22qDel-ASD Group** | | | **22qDel-No ASD Group** | | | **22qDel-ASD Group** | | | **22qDel-No ASD Group** | **22qDel- ASD Group** | **22qDel-ASD vs 22qDel-No ASD** |
| **brain** | **hemisphere** | **t** | **p** | **q** | **F** | **p** | **q** | **F** | **p** | **q** |  |  |  |
| Overall Mean Thickness |  | -1.06 | 0.29 | 0.34 | 36.41 | 0.00 | 0.00 | 25.45 | 0.00 | 0.00 | 5.5-25.2 | 5.5-23 | NA |
| Mean Thickness | L | -0.79 | 0.43 | 0.48 | 38.62 | 0.00 | 0.00 | 23.72 | 0.00 | 0.00 | 5.5-25.2 | 5.5-21.1 | NA |
| Mean Thickness | R | -1.31 | 0.19 | 0.24 | 31.98 | 0.00 | 0.00 | 29.91 | 0.00 | 0.00 | 5.5-25.2 | 5.5-25.2 | NA |
| Frontal Thickness | L | -2.06 | 0.04 | 0.06 | 43.12 | 0.00 | 0.00 | 32.07 | 0.00 | 0.00 | 5.5-25.2 | 5.5-25.2 | NA |
| Frontal Thickness | R | -2.43 | 0.02 | 0.03 | 33.52 | 0.00 | 0.00 | 40.64 | 0.00 | 0.00 | 5.5-25.2 | 5.5-25.2 | NA |
| Temporal Thickness | L | -0.56 | 0.58 | 0.60 | 9.08 | 0.00 | 0.01 | 4.32 | 0.04 | 0.06 | 5.5-25.2 | NA | NA |
| Temporal Thickness | R | -0.87 | 0.39 | 0.44 | 12.08 | 0.00 | 0.00 | 8.25 | 0.01 | 0.01 | 5.5-25.2 | 5.5-25.2 | NA |
| Parietal Thickness | L | -1.52 | 0.13 | 0.17 | 38.54 | 0.00 | 0.00 | 25.17 | 0.00 | 0.00 | 5.5-25.2 | 5.5-25.2 | NA |
| Parietal Thickness | R | -1.79 | 0.08 | 0.10 | 30.80 | 0.00 | 0.00 | 21.79 | 0.00 | 0.00 | 5.5-25.2 | 5.5-21.5 | NA |
| Occipital Thickness | L | -0.09 | 0.93 | 0.93 | 15.83 | 0.00 | 0.00 | 11.24 | 0.00 | 0.00 | 5.5-25.2 | 5.5-16.5 | NA |
| Occipital Thickness | R | -0.63 | 0.53 | 0.56 | 8.47 | 0.00 | 0.00 | 13.15 | 0.00 | 0.00 | 5.5-17.9 | 5.5-22.2 | NA |

# e-Table 18. Individual ROI Table for 22qDel-ASD vs. 22qDel-no ASD

|  |  | Parametric Effects of Group  (22qDel-No ASD as Reference) | | | Non-parametric (smooth) Effects of Age | | | | | | Age range(s) when significant change is taking place | | Age range(s) when smoothed effects of age differed between groups |
| --- | --- | --- | --- | --- | --- | --- | --- | --- | --- | --- | --- | --- | --- |
|  |  | **22qDel-ASD Group** | | | **22qDel-No ASD Group** | | | **22qDel-ASD Group** | | | **22qDel-No ASD Group** | **22qDel-ASD Group** | **22qDel-ASD vs 22qDel-No ASD** |
| **brain** | **hemisphere** | **t** | **p** | **q** | **F** | **p** | **q** | **F** | **p** | **q** |  |  |  |
| Caudal Anterior Cingulate Area | L | 3.28 | 0.00 | 0.03 | 0.00 | 1.00 | 1.00 | 1.82 | 0.22 | 0.45 | NA | NA | NA |
| Caudal Middle Frontal Area | L | 0.89 | 0.37 | 0.57 | 5.33 | 0.02 | 0.14 | 0.18 | 0.67 | 0.83 | NA | NA | NA |
| Frontal Pole Area | L | -0.19 | 0.85 | 0.94 | 4.87 | 0.00 | 0.07 | 2.69 | 0.08 | 0.28 | NA | NA | NA |
| Medial Orbitofrontal Area | L | 1.87 | 0.06 | 0.25 | 2.18 | 0.10 | 0.32 | 4.33 | 0.01 | 0.13 | NA | NA | NA |
| Lateral Orbitofrontal Area | L | 1.25 | 0.21 | 0.45 | 0.52 | 0.52 | 0.69 | 1.73 | 0.19 | 0.45 | NA | NA | NA |
| Paracentral Area | L | 1.45 | 0.15 | 0.40 | 0.75 | 0.39 | 0.57 | 5.88 | 0.02 | 0.13 | NA | NA | NA |
| Pars Opercularis Area | L | 0.90 | 0.37 | 0.57 | 2.15 | 0.11 | 0.36 | 0.04 | 0.85 | 0.94 | NA | NA | NA |
| Pars Orbitalis Area | L | -0.58 | 0.57 | 0.74 | 1.87 | 0.08 | 0.28 | 0.03 | 0.88 | 0.94 | NA | NA | NA |
| Pars Triangularis Area | L | -0.14 | 0.89 | 0.94 | 0.76 | 0.38 | 0.57 | 5.78 | 0.02 | 0.13 | NA | NA | NA |
| Precentral Area | L | -0.82 | 0.41 | 0.58 | 1.21 | 0.27 | 0.49 | 0.95 | 0.33 | 0.55 | NA | NA | NA |
| Rostral Anterior Cingulate Area | L | 1.25 | 0.21 | 0.45 | 0.09 | 0.77 | 0.89 | 1.10 | 0.37 | 0.57 | NA | NA | NA |
| Rostral Middle Frontal Area | L | 1.35 | 0.18 | 0.44 | 3.13 | 0.08 | 0.28 | 4.22 | 0.01 | 0.09 | NA | NA | NA |
| Superior Frontal Area | L | -0.08 | 0.94 | 0.96 | 4.36 | 0.00 | 0.03 | 2.12 | 0.07 | 0.27 | 9.6-12.4 ; 15.6-18 ; 23.2-23.6 | NA | 5.5-7.9 ; 11.1-15.9 ; 19.3-22.5 |
| Caudal Anterior Cingulate Area | R | 1.23 | 0.22 | 0.45 | 0.29 | 0.59 | 0.76 | 0.79 | 0.38 | 0.57 | NA | NA | NA |
| Caudal Middle Frontal Area | R | 1.05 | 0.30 | 0.52 | 3.28 | 0.03 | 0.20 | 3.93 | 0.05 | 0.23 | NA | NA | NA |
| Frontal Pole Area | R | 0.00 | 1.00 | 1.00 | 1.95 | 0.13 | 0.39 | 5.75 | 0.00 | 0.02 | NA | 16.4-22.9 | 5.5-5.9 ; 10.5-16.9 ; 20.7-25.2 |
| Medial Orbitofrontal Area | R | -0.29 | 0.77 | 0.89 | 2.26 | 0.05 | 0.23 | 1.62 | 0.16 | 0.40 | NA | NA | NA |
| Lateral Orbitofrontal Area | R | 0.82 | 0.42 | 0.58 | 0.10 | 0.75 | 0.89 | 0.15 | 0.70 | 0.85 | NA | NA | NA |
| Paracentral Area | R | 0.44 | 0.66 | 0.82 | 0.67 | 0.41 | 0.58 | 4.14 | 0.02 | 0.13 | NA | NA | NA |
| Pars Opercularis Area | R | 1.43 | 0.15 | 0.40 | 0.98 | 0.35 | 0.57 | 0.03 | 0.89 | 0.94 | NA | NA | NA |
| Pars Orbitalis Area | R | 0.88 | 0.38 | 0.57 | 0.35 | 0.55 | 0.73 | 0.00 | 0.99 | 1.00 | NA | NA | NA |
| Pars Triangularis Area | R | 1.43 | 0.16 | 0.40 | 0.93 | 0.37 | 0.57 | 1.57 | 0.21 | 0.45 | NA | NA | NA |
| Precentral Area | R | -0.45 | 0.65 | 0.82 | 3.99 | 0.05 | 0.23 | 3.02 | 0.06 | 0.25 | NA | NA | NA |
| Rostral Anterior Cingulate Area | R | 0.76 | 0.45 | 0.62 | 0.11 | 0.74 | 0.89 | 0.01 | 0.91 | 0.94 | NA | NA | NA |
| Rostral Middle Frontal Area | R | -0.87 | 0.39 | 0.57 | 0.22 | 0.68 | 0.83 | 1.98 | 0.19 | 0.45 | NA | NA | NA |
| Superior Frontal Area | R | 1.89 | 0.06 | 0.25 | 0.22 | 0.64 | 0.80 | 3.95 | 0.00 | 0.08 | NA | NA | NA |
| Banks STS Area | R | -0.13 | 0.89 | 0.94 | 1.46 | 0.20 | 0.45 | 3.66 | 0.01 | 0.12 | NA | NA | NA |
| Entorhinal Area | R | 0.92 | 0.36 | 0.57 | 3.29 | 0.02 | 0.14 | 3.35 | 0.02 | 0.13 | NA | NA | NA |
| Fusiform Area | R | 1.09 | 0.28 | 0.50 | 1.77 | 0.23 | 0.46 | 2.08 | 0.11 | 0.36 | NA | NA | NA |
| Inferior Temporal Area | R | 1.97 | 0.05 | 0.23 | 0.02 | 0.88 | 0.94 | 2.14 | 0.09 | 0.29 | NA | NA | NA |
| Insula Area | R | 1.48 | 0.14 | 0.40 | 1.76 | 0.19 | 0.45 | 7.40 | 0.01 | 0.10 | NA | NA | NA |
| Middle Temporal Area | R | 0.29 | 0.77 | 0.89 | 1.42 | 0.19 | 0.45 | 3.93 | 0.00 | 0.08 | NA | NA | NA |
| Parahippocampal Area | R | 1.00 | 0.32 | 0.55 | 0.02 | 0.90 | 0.94 | 0.05 | 0.83 | 0.94 | NA | NA | NA |
| Superior Temporal Area | R | 1.57 | 0.12 | 0.37 | 0.73 | 0.39 | 0.57 | 4.39 | 0.01 | 0.13 | NA | NA | NA |
| Temporal Pole Area | R | 2.10 | 0.04 | 0.20 | 1.37 | 0.24 | 0.46 | 0.93 | 0.37 | 0.57 | NA | NA | NA |
| Transverse Temporal Area | R | 0.81 | 0.42 | 0.58 | 1.37 | 0.24 | 0.46 | 2.33 | 0.13 | 0.39 | NA | NA | NA |
| Inferior Parietal Area | L | 0.22 | 0.83 | 0.94 | 1.95 | 0.17 | 0.42 | 1.93 | 0.12 | 0.38 | NA | NA | NA |
| Isthmus Cingulate Area | L | 0.92 | 0.36 | 0.57 | 0.98 | 0.32 | 0.55 | 0.76 | 0.59 | 0.76 | NA | NA | NA |
| Postcentral Area | L | 0.74 | 0.46 | 0.63 | 1.42 | 0.20 | 0.45 | 6.17 | 0.00 | 0.02 | NA | 7.9-9.2 ; 18.4-21.7 | 10.7-15.7 ; 21.9-24.9 |
| Posterior Cingulate Area | L | 0.66 | 0.51 | 0.69 | 1.37 | 0.24 | 0.46 | 4.02 | 0.02 | 0.14 | NA | NA | NA |
| Precuneus Area | L | 0.11 | 0.91 | 0.94 | 0.87 | 0.32 | 0.55 | 5.74 | 0.02 | 0.13 | NA | NA | NA |
| Superior Parietal Area | L | 0.13 | 0.90 | 0.94 | 3.31 | 0.07 | 0.27 | 5.73 | 0.02 | 0.13 | NA | NA | NA |
| Supramarginal Area | L | 2.67 | 0.01 | 0.11 | 1.45 | 0.27 | 0.49 | 0.66 | 0.63 | 0.80 | NA | NA | NA |
| Inferior Parietal Area | R | 1.16 | 0.25 | 0.47 | 1.29 | 0.31 | 0.54 | 2.84 | 0.04 | 0.20 | NA | NA | NA |
| Isthmus Cingulate Area | R | 1.98 | 0.05 | 0.23 | 0.16 | 0.85 | 0.94 | 2.14 | 0.14 | 0.40 | NA | NA | NA |
| Postcentral Area | R | 0.03 | 0.98 | 1.00 | 1.44 | 0.23 | 0.46 | 6.35 | 0.00 | 0.02 | NA | 14.6-20.3 | 10.3-14.5 ; 20.9-23.3 |
| Posterior Cingulate Area | R | 0.65 | 0.52 | 0.69 | 1.60 | 0.27 | 0.49 | 2.06 | 0.15 | 0.40 | NA | NA | NA |
| Precuneus Area | R | 2.08 | 0.04 | 0.20 | 1.55 | 0.20 | 0.45 | 2.08 | 0.15 | 0.40 | NA | NA | NA |
| Superior Parietal Area | R | -0.12 | 0.90 | 0.94 | 1.39 | 0.24 | 0.46 | 12.94 | 0.00 | 0.02 | NA | 5.5-25.2 | NA |
| Supramarginal Area | R | 0.30 | 0.76 | 0.89 | 2.87 | 0.04 | 0.20 | 4.47 | 0.04 | 0.20 | NA | NA | NA |

# e- Table 19. 22q Del-ASD vs 22qDel-no ASD, covarying for comorbid Psychosis Spectrum Symptoms

|  |  | Parametric Effects of Group  (22qDel-No ASD as Reference) | | | Non-parametric (smooth) Effects of Age | | | | | | | | Age range(s) when significant change is taking place | | | | Age range(s) when smoothed effects of age differed between groups | |  |
| --- | --- | --- | --- | --- | --- | --- | --- | --- | --- | --- | --- | --- | --- | --- | --- | --- | --- | --- | --- |
|  |  | **22qDel-ASD Group** | | | **22qDel-No ASD Group** | | | | **22qDel-ASD Group** | | | | **22qDel-No ASD Group** | | **22qDel-ASD Group** | | **22qDel-ASD vs 22qDel-No ASD** | |  |
| **brain** | **hemisphere** | **T** | **p** | **q** | | **F** | **p** | **q** | | **F** | **p** | **q** | |  | |  | |  | |
| Overall Mean Thickness |  | -0.61 | 0.54 | 0.60 | | 28.18 | 0.00 | 0.00 | | 19.76 | 0.00 | 0.00 | | 5.5-25.2 | | 5.5-25.2 | | NA | |
| Mean Thickness | L | -0.36 | 0.72 | 0.77 | | 31.41 | 0.00 | 0.00 | | 16.06 | 0.00 | 0.00 | | 5.5-25.2 | | 5.5-20.9 | | NA | |
| Mean Thickness | R | -0.85 | 0.40 | 0.45 | | 23.39 | 0.00 | 0.00 | | 30.64 | 0.00 | 0.00 | | 5.5-25.2 | | 5.5-25.2 | | NA | |
| Frontal Thickness | L | -1.58 | 0.12 | 0.15 | | 36.47 | 0.00 | 0.00 | | 21.54 | 0.00 | 0.00 | | 5.5-25.2 | | 5.5-25.2 | | NA | |
| Frontal Thickness | R | -1.98 | 0.05 | 0.07 | | 26.60 | 0.00 | 0.00 | | 29.65 | 0.00 | 0.00 | | 5.5-25.2 | | 5.5-25.2 | | NA | |
| Temporal Thickness | L | 0.08 | 0.93 | 0.97 | | 7.44 | 0.01 | 0.01 | | 1.86 | 0.18 | 0.21 | | 5.5-25.2 | | NA | | NA | |
| Temporal Thickness | R | -0.58 | 0.56 | 0.62 | | 9.26 | 0.00 | 0.01 | | 5.04 | 0.03 | 0.04 | | 5.5-25.2 | | 5.5-25.2 | | NA | |
| Parietal Thickness | L | -1.29 | 0.20 | 0.23 | | 27.56 | 0.00 | 0.00 | | 23.93 | 0.00 | 0.00 | | 5.5-25.2 | | 5.5-25.2 | | NA | |
| Parietal Thickness | R | -1.64 | 0.10 | 0.13 | | 21.18 | 0.00 | 0.00 | | 19.00 | 0.00 | 0.00 | | 5.5-25.2 | | 5.5-25.2 | | NA | |
| Occipital Thickness | L | 0.31 | 0.76 | 0.80 | | 8.49 | 0.00 | 0.00 | | 7.81 | 0.00 | 0.00 | | 5.5-20.7 | | 5.5-15.9 | | NA | |
| Occipital Thickness | R | 0.04 | 0.96 | 0.97 | | 8.17 | 0.00 | 0.00 | | 8.40 | 0.00 | 0.00 | | 5.5-18.1 | | 5.5-19.3 | | NA | |
|  |  |  |  |  | |  |  |  | |  |  |  | |  | |  | |  | |
| Total Surface Area |  | 2.75 | 0.01 | 0.01 | | 11.14 | 0.00 | 0.00 | | 7.52 | 0.00 | 0.00 | | 5.5-25.2 | | 5.5-10.1 ; 12.6-19.5 | | 5.5-7.3 ; 11.5-14.7 | |
| Frontal Area | L | 2.83 | 0.01 | 0.01 | | 18.71 | 0.00 | 0.00 | | 6.50 | 0.00 | 0.00 | | 5.5-25.2 | | 5.5-10.7 ; 13.3-19.6 | | 5.5-9.1 ; 13.1-15.3 ; 23.9-25.2 | |
| Frontal Area | R | 2.42 | 0.02 | 0.03 | | 5.78 | 0.02 | 0.03 | | 5.29 | 0.00 | 0.00 | | 5.5-25.2 | | 5.5-10.3 ; 13.2-19.4 | | 5.5-7.1 ; 11.9-14.9 | |
| Temporal Area | L | 4.23 | 0.00 | 0.00 | | 4.04 | 0.05 | 0.07 | | 3.25 | 0.02 | 0.04 | | NA | | 5.5-9.6 ; 14.7-19.3 | | 5.5-6.9 ; 13.3-14.7 | |
| Temporal Area | R | 4.17 | 0.00 | 0.00 | | 4.51 | 0.04 | 0.06 | | 5.22 | 0.00 | 0.00 | | NA | | 5.5-9.8 ; 12.8-19.2 | | 5.5-6.1 ; 11.5-14.5 | |
| Parietal Area | L | 1.74 | 0.09 | 0.11 | | 9.92 | 0.00 | 0.01 | | 6.63 | 0.00 | 0.00 | | 5.5-25.2 | | 5.5-8.1 ; 12.5-19.7 | | 5.5-5.5 ; 11.7-14.7 | |
| Parietal Area | R | 1.86 | 0.07 | 0.09 | | 2.68 | 0.07 | 0.09 | | 7.00 | 0.00 | 0.00 | | NA | | 5.5-8 ; 12.2-19.6 | | 10.5-14.7 ; 19.7-20.9 | |
| Occipital Area | L | -0.03 | 0.97 | 0.97 | | 5.33 | 0.03 | 0.04 | | 1.67 | 0.13 | 0.16 | | 11.2-25.2 | | NA | | 23.7-25.2 | |
| Occipital Area | R | 1.91 | 0.06 | 0.08 | | 1.85 | 0.18 | 0.21 | | 2.56 | 0.04 | 0.06 | | NA | | NA | | NA | |

# e-Table 20a. Neurodevelopmental CT and SA trajectories in 22qDup-ASD vs. 22qDup-no ASD

|  |  | Parametric Effects of Group  (22qDup-No ASD as Reference) | | | Non-parametric (smooth) Effects of Age | | | | | | Age range(s) when significant change is taking place | | Age range(s) when smoothed effects of age differed between groups |
| --- | --- | --- | --- | --- | --- | --- | --- | --- | --- | --- | --- | --- | --- |
|  |  | **22qDup-ASD Group** | | | **22qDup-No ASD Group** | | | **22qDup-ASD Group** | | | **22qDup-No ASD Group** | **22qDup-ASD Group** | **22qDup-ASD vs 22qDup-No ASD** |
| **Brain Measure** | **Hemisphere** | **T** | **p** | **q** | **F** | **p** | **q** | **F** | **p** | **q** |  |  |  |
| Overall Mean Thickness |  | 1.76 | 0.09 | 0.15 | 12.70 | 0.00 | 0.01 | 27.03 | 0.00 | 0.00 | 6.7-24.8 | 6.7-24.8 | NA |
| Frontal Thickness | L | 0.87 | 0.39 | 0.42 | 14.03 | 0.00 | 0.00 | 20.09 | 0.00 | 0.00 | 6.7-24.8 | 6.7-24.8 | NA |
| Frontal Thickness | R | 2.29 | 0.03 | 0.08 | 7.27 | 0.01 | 0.03 | 18.09 | 0.00 | 0.00 | 6.7-24.8 | 6.7-24.8 | NA |
| Temporal Thickness | L | 1.08 | 0.29 | 0.35 | 4.77 | 0.04 | 0.09 | 7.48 | 0.01 | 0.03 | NA | 6.7-24.8 | NA |
| Temporal Thickness | R | 0.96 | 0.34 | 0.39 | 7.51 | 0.01 | 0.03 | 6.15 | 0.02 | 0.05 | 6.7-24.8 | NA | NA |
| Parietal Thickness | L | 2.01 | 0.05 | 0.12 | 7.18 | 0.00 | 0.02 | 20.97 | 0.00 | 0.00 | 13.7-20.2 | 6.7-24.8 | NA |
| Parietal Thickness | R | 1.44 | 0.16 | 0.22 | 6.93 | 0.01 | 0.04 | 17.84 | 0.00 | 0.00 | 6.7-24.8 | 6.7-24.8 | NA |
| Occipital Thickness | L | 1.88 | 0.07 | 0.14 | 8.18 | 0.00 | 0.02 | 21.09 | 0.00 | 0.00 | 13.2-21.3 | 6.7-24.8 | 6.7-9.6; 14.4-15.7 |
| Occipital Thickness | R | 0.63 | 0.53 | 0.55 | 17.69 | 0.00 | 0.00 | 21.42 | 0.00 | 0.00 | 6.7-24.8 | 6.7-15.6 | 6.7-9.8; 12.4-17.1 |
|  |  |  |  |  |  |  |  |  |  |  |  |  |  |
| Total Surface Area |  | 1.83 | 0.08 | 0.15 | 1.98 | 0.17 | 0.23 | 2.57 | 0.09 | 0.16 | NA | NA | NA |
| Frontal Area | L | 1.65 | 0.11 | 0.17 | 1.16 | 0.29 | 0.35 | 1.97 | 0.17 | 0.23 | NA | NA | NA |
| Frontal Area | R | 1.63 | 0.11 | 0.17 | 1.11 | 0.30 | 0.35 | 2.25 | 0.14 | 0.21 | NA | NA | NA |
| Temporal Area | L | 1.66 | 0.11 | 0.17 | 2.05 | 0.16 | 0.22 | 2.94 | 0.05 | 0.12 | NA | NA | NA |
| Temporal Area | R | 1.93 | 0.06 | 0.13 | 2.08 | 0.16 | 0.22 | 1.12 | 0.37 | 0.40 | NA | NA | NA |
| Parietal Area | L | 3.16 | 0.00 | 0.02 | 1.58 | 0.22 | 0.27 | 2.23 | 0.19 | 0.25 | NA | NA | NA |
| Parietal Area | R | 1.79 | 0.08 | 0.15 | 3.04 | 0.09 | 0.16 | 1.24 | 0.34 | 0.39 | NA | NA | NA |
| Occipital Area | L | -2.37 | 0.02 | 0.07 | 0.02 | 0.89 | 0.89 | 0.66 | 0.47 | 0.50 | NA | NA | NA |
| Occipital Area | R | -2.26 | 0.03 | 0.08 | 0.13 | 0.72 | 0.74 | 2.31 | 0.08 | 0.15 | NA | NA | NA |

# e-Table 20b. Neurodevelopmental CT trajectories in 22qDup-ASD vs. 22qDup-no ASD (without ICV adjustment)

|  |  | Parametric Effects of Group  (22qDup-No ASD as Reference) | | | Non-parametric (smooth) Effects of Age | | | | | | Age range(s) when significant change is taking place | | Age range(s) when smoothed effects of age differed between groups |
| --- | --- | --- | --- | --- | --- | --- | --- | --- | --- | --- | --- | --- | --- |
|  |  | **22qDup-ASD Group** | | | **22qDup-No ASD Group** | | | **22qDup-ASD Group** | | | **22qDup-No ASD Group** | **22qDup-ASD Group** | **22qDup-ASD vs 22qDup-No ASD** |
| **brain** | **hemisphere** | **T** | **p** | **q** | **F** | **p** | **q** | **F** | **p** | **q** |  |  |  |
| Mean Thickness |  | 1.74 | 0.09 | 0.15 | 7.53 | 0.01 | 0.03 | 22.00 | 0.00 | 0.00 | 6.7-24.8 | 6.7-24.8 | NA |
| Frontal Thickness | L | 0.50 | 0.62 | 0.64 | 7.13 | 0.01 | 0.04 | 13.17 | 0.00 | 0.00 | 6.7-24.8 | 6.7-24.8 | NA |
| Frontal Thickness | R | 1.75 | 0.09 | 0.15 | 4.65 | 0.04 | 0.08 | 11.80 | 0.00 | 0.00 | NA | 6.7-24.8 | NA |
| Temporal Thickness | L | 0.44 | 0.66 | 0.68 | 3.57 | 0.07 | 0.14 | 5.16 | 0.03 | 0.08 | NA | NA | NA |
| Temporal Thickness | R | 0.70 | 0.49 | 0.53 | 5.75 | 0.02 | 0.06 | 4.46 | 0.05 | 0.09 | NA | NA | NA |
| Parietal Thickness | L | 2.44 | 0.02 | 0.06 | 5.37 | 0.03 | 0.07 | 18.91 | 0.00 | 0.00 | NA | 6.7-24.8 | NA |
| Parietal Thickness | R | 1.48 | 0.15 | 0.22 | 6.58 | 0.01 | 0.04 | 18.93 | 0.00 | 0.00 | 6.7-24.8 | 6.7-24.8 | NA |
| Occipital Thickness | L | 2.99 | 0.01 | 0.02 | 4.50 | 0.04 | 0.08 | 25.73 | 0.00 | 0.00 | NA | 6.7-24.8 | NA |
| Occipital Thickness | R | 1.36 | 0.19 | 0.26 | 9.68 | 0.00 | 0.02 | 17.49 | 0.00 | 0.00 | 6.7-24.8 | 6.7-19.3 | 12.7-14.6 |

# e-Table 21. Individual ROI Table for 22qDup-ASD vs. 22qDup-no ASD

|  |  | Parametric Effects of Group (22q Dup-No ASD as Reference) | | | Non-parametric (smooth) Effects of Age | | | | | | Age range(s) when significant change is taking place | | Age range(s) when smoothed effects of age differed between groups |
| --- | --- | --- | --- | --- | --- | --- | --- | --- | --- | --- | --- | --- | --- |
|  |  | **22qDup-ASD Group** | | | **22qDup-No ASD Group** | | | **22qDup-ASD Group** | | | **22qDup-No ASD Group** | **22qDup-ASD Group** | **22qDup-ASD vs 22qDup-No ASD** |
| **Brain Region** | **hemisphere** | **t** | **p** | **q** | **F** | **p** | **q** | **F** | **p** | **q** |  |  |  |
| Cuneus Thickness | L | 3.01 | 0.00 | 0.01 | 2.32 | 0.14 | 0.17 | 13.63 | 0.00 | 0.00 | NA | 6.7-17.2 | 6.7-10.5 ; 13.1-21 |
| Lateral Occipital Thickness | L | 0.50 | 0.62 | 0.68 | 5.18 | 0.03 | 0.05 | 24.61 | 0.00 | 0.00 | 6.7-24.8 | 6.7-24.8 | NA |
| Lingual Thickness | R | 1.41 | 0.17 | 0.19 | 4.30 | 0.02 | 0.03 | 11.49 | 0.00 | 0.00 | 16.4-19.9 | 6.7-15.2 | 6.7-9.6 ; 12.5-16.2 |
| Pericalcarine Thickness | R | 0.17 | 0.87 | 0.87 | 2.63 | 0.06 | 0.09 | 7.29 | 0.00 | 0.00 | NA | 6.7-9.9 | 6.7-8 |
| Cuneus Thickness | R | 1.83 | 0.08 | 0.11 | 8.46 | 0.01 | 0.01 | 14.47 | 0.00 | 0.00 | 6.7-24.8 | 6.7-15.6 | 6.7-9.8 ; 12.7-16.9 |
| Lateral Occipital Thickness | R | -1.59 | 0.12 | 0.16 | 17.88 | 0.00 | 0.00 | 27.33 | 0.00 | 0.00 | 6.7-24.8 | 6.7-24.8 | NA |
| Lingual Thickness | R | 1.41 | 0.17 | 0.19 | 4.30 | 0.02 | 0.03 | 11.49 | 0.00 | 0.00 | 16.4-19.9 | 6.7-15.2 | 6.7-9.6 ; 12.5-16.2 |
| Pericalcarine Thickness | R | 0.17 | 0.87 | 0.87 | 2.63 | 0.06 | 0.09 | 7.29 | 0.00 | 0.00 | NA | 6.7-9.9 | 6.7-8 |
