# Supplementary material for: Longitudinal trajectories of cortical development in 22q11.2 copy number variants and typically developing controls
Source: Mol Psychiatry. 2022 Jul 27;27(10):4181–90. doi: 10.1038/s41380-022-01681-w (PMC9718681; doi:10.1038/s41380-022-01681-w)

e-Figure 1. Flow chart of analysis pipeline

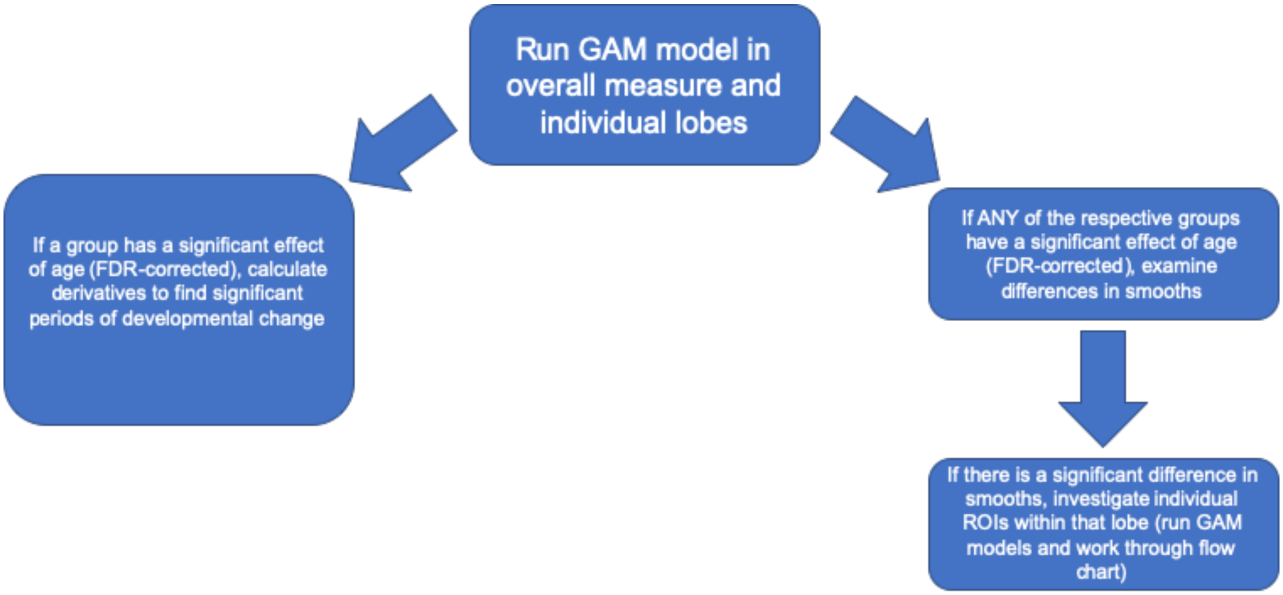

eFigure 2. Partial residual plots of neurodevelopmental cortical thickness trajectories of cortical lobes in 22qDel-ASD vs. 22qDel-no ASD. The partial residual plots reflect the relationship between age and the respective neuroimaging measures, given the other covariates in the model. Shaded regions are +/- standard errors. The bars underneath the age plots reflect the derivative of the slope, i.e., the rate of change taking place at a particular age. Darker blue indicates that there is a stronger decrease in CT taking place at that particular age, while brighter red indicates a stronger increase in CT. Control age effects and derivatives are shown for visualization purposes only.

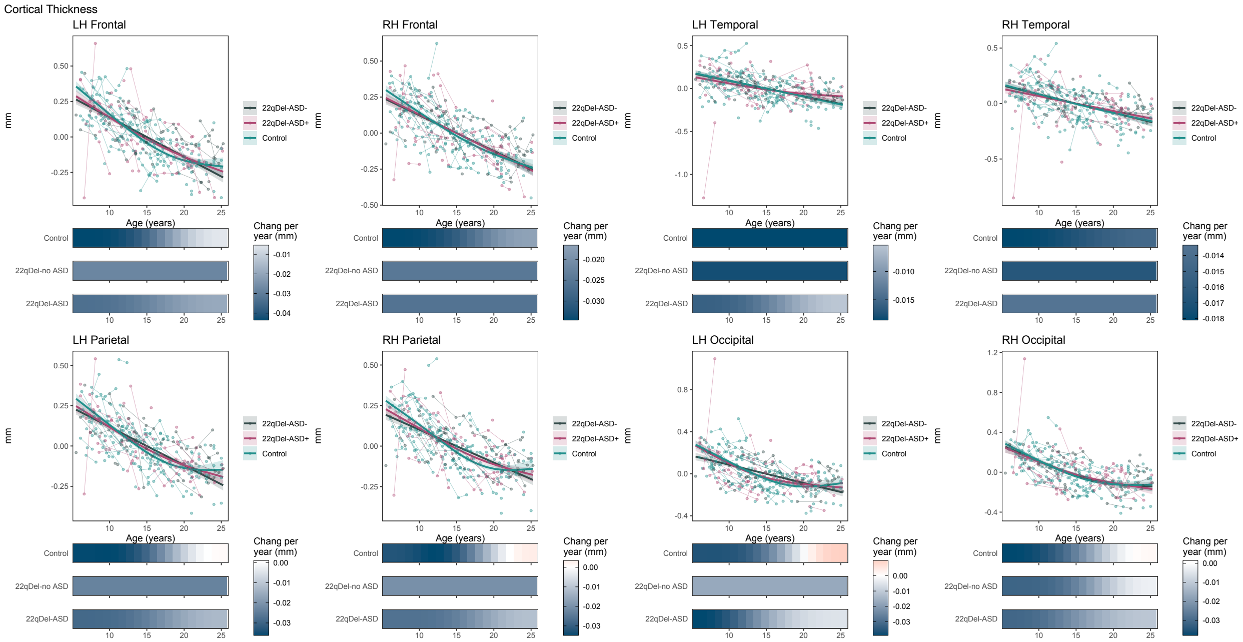

eFigure 3. Partial residual plots of neurodevelopmental surface area trajectories of cortical lobes in 22qDel-ASD vs. 22qDel-no ASD. The partial residual plots reflect the relationship between age and the respective neuroimaging measures, given the other covariates in the model. Shaded regions are +/- standard errors. The bars underneath the age plots reflect the derivative of the slope, i.e., the rate of change taking place at a particular age. Darker blue indicates that there is a stronger decrease in SA taking place at that particular age, while brighter red indicates a stronger increase in SA. Control age effects and derivatives are shown for visualization purposes only.

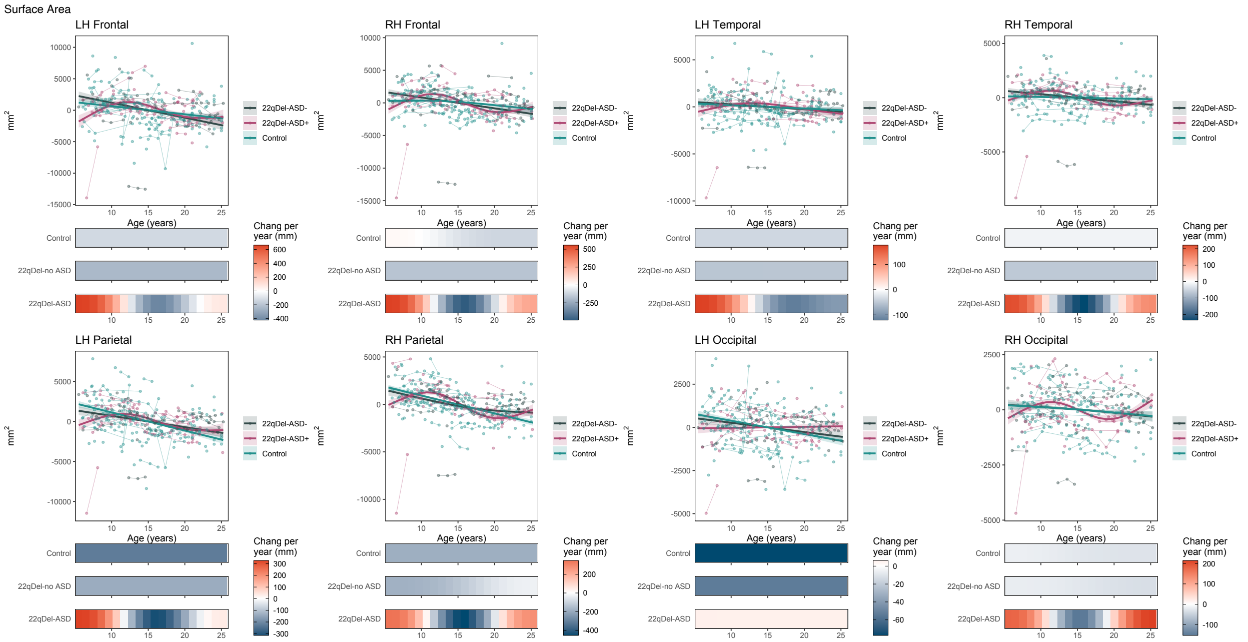

eFigure 4. Partial residual plots of neurodevelopmental cortical thickness trajectories of cortical lobes in 22qDup-ASD vs. 22qDup-no ASD. The partial residual plots reflect the relationship between age and the respective neuroimaging measures, given the other covariates in the model. Shaded regions are +/- standard errors. The bars underneath the age plots reflect the derivative of the slope, i.e., the rate of change taking place at a particular age. Darker blue indicates that there is a stronger decrease in CT taking place at that particular age, while brighter red indicates a stronger increase in CT. Control age effects and derivatives are shown for visualization purposes only.

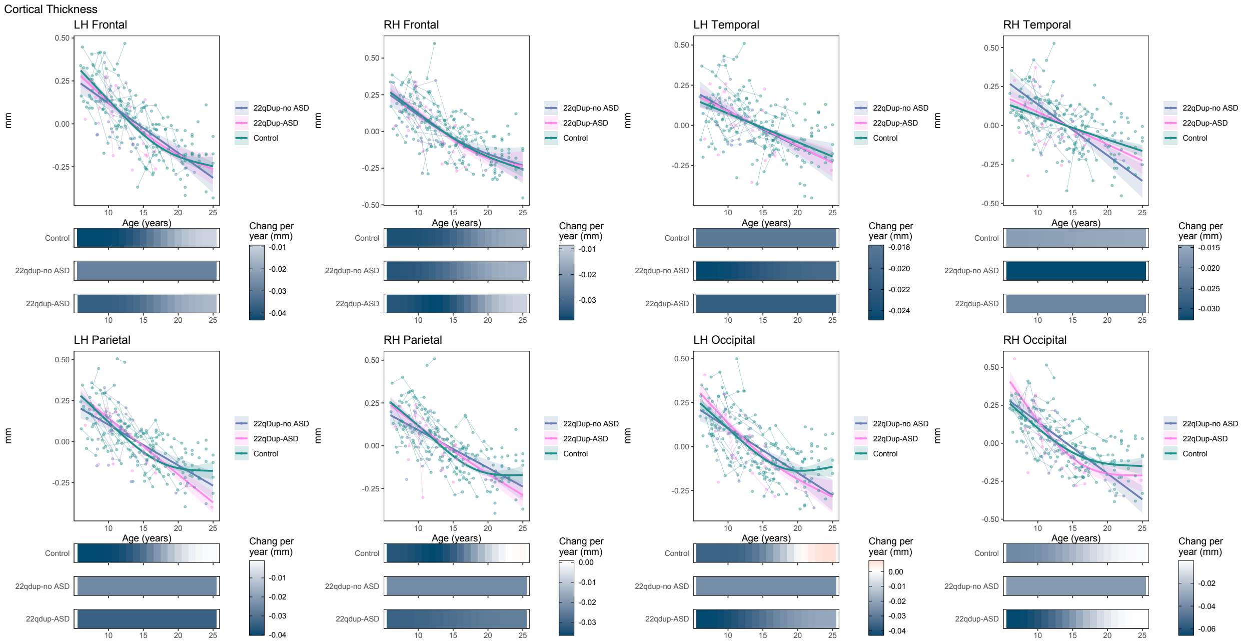

eFigure 5. Partial residual plots of neurodevelopmental surface area trajectories of cortical lobes in 22qDupASD vs. 22qDup-no ASD. The partial residual plots reflect the relationship between age and the respective neuroimaging measures, given the other covariates in the model. Shaded regions are +/- standard errors. The bars underneath the age plots reflect the derivative of the slope, i.e., the rate of change taking place at a particular age. Darker blue indicates that there is a stronger decrease in SA taking place at that particular age, while brighter red indicates a stronger increase in SA.Control age effects and derivatives are shown for visualization purposes only.

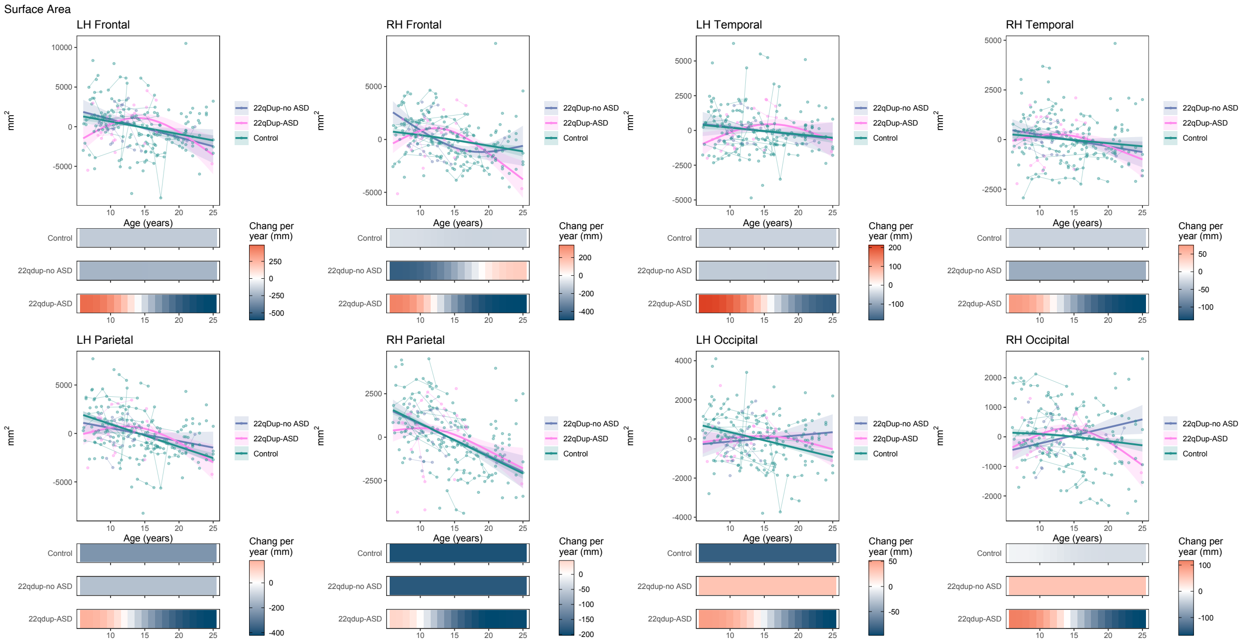

Supplement: Supplementary file 3 — Supplementary Figures [file 41380_2022_1681_MOESM3_ESM.pdf]
